# FeBr_3_-catalysed synthesis of 3-aroylimidazo[1,2-*a*]pyridine and 3,3′-(arylmethylene)bis(2-phenylimidazo[1,2-*a*]pyridines) derivatives from 2-arylimidazo[1,2-*a*]pyridines and aromatic aldehydes: an investigation about mechanistic insights†

**DOI:** 10.1039/d4ra05198j

**Published:** 2024-09-18

**Authors:** Tran Quang Hung, Ban Van Phuc, Mai Phuong Nguyen, Tuan Linh Tran, Dang Van Do, Ha Thanh Nguyen, Van Tuyen Nguyen, Hien Nguyen, Tuan Thanh Dang

**Affiliations:** a Institute of Chemistry, Vietnam Academy of Science and Technology Vietnam tqhung@ich.vast.vn; b Faculty of Chemistry, Hanoi University of Science, Vietnam National University (VNU) Vietnam dangthanhtuan@hus.edu.vn; c Faculty of Chemistry, Hanoi National University of Education (HNUE) Vietnam; d Graduate University of Science and Technology, Vietnam Academy of Science and Technology Vietnam

## Abstract

In a new approach, a series of 3-aroylimidazo[1,2-*a*]pyridine derivatives were prepared in high yields. This approach revealed the direct Fe-catalyzed functionalization of imidazo[1,2-*a*]pyridine derivatives with aryl aldehydes *via* an aerobic oxidative cross-dehydrogenative coupling process. This transformation occurred in the presence of air, and FeBr_3_ served as a homogeneous Lewis catalyst. O_2_ was found to be the principal oxidant responsible for the method's success. Interestingly, when these reactions were carried out under an argon atmosphere, 3,3′-(arylmethylene)bis(2-phenylimidazo[1,2-*a*]pyridines) derivatives were prepared in good yields.

## Abstract 2

FeBr_3_-catalysed synthesis of either 3-aroylimidazo[1,2-*a*]pyridines or 3,3′-(arylmethylene)bis(2-phenylimidazo[1,2-*a*]pyridines) derivatives from the same starting materials such as 2-arylimidazo[1,2-*a*]pyridines and aromatic aldehydes were reported.

## Graphical abstract

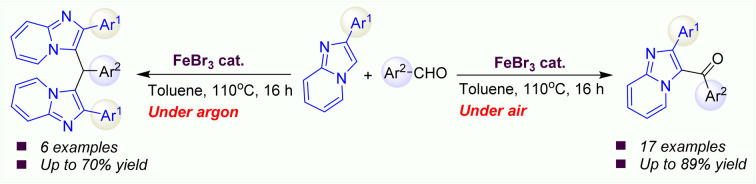

## Introduction

**Fig. 1:**
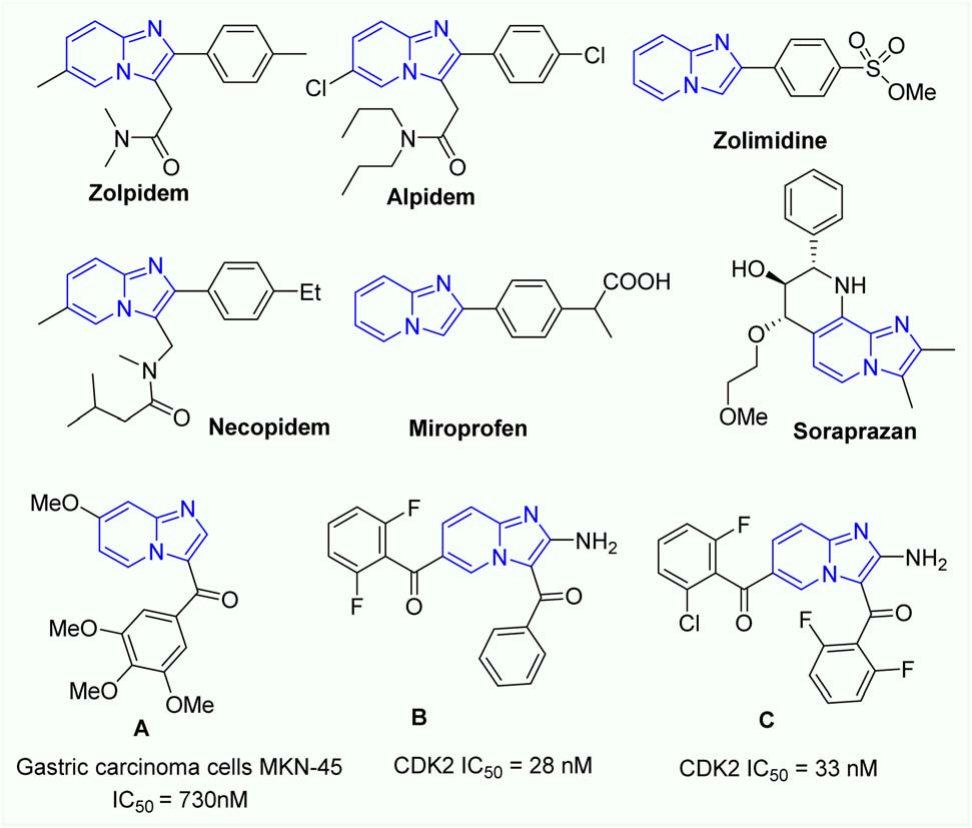
Imidazo[1,2-*a*]pyridine derivatives with various biological activities.

In the field of drug discovery, fused N-heterocycles have long been recognized as essential targets.^1–4^ Specifically, imidazo[1,2-*a*]pyridine derivatives are crucial building blocks in several important drugs and bioactive molecules due to their wide bioactivities.^5^ Several commercial pharmaceutics contain an imidazo[1,2-*a*]pyridine core, including anxiolytics Alpidem, Necopidem, Soraprazan, Saripidem and Zolimidine, as well as Zolpidem, which is used to treat brain abnormalities (Fig. 1).^5^ Furthermore, the antibacterial, anticancer, anti-inflammatory, antiprotozoal, antiviral, antiparasitic, analgesic, and antipyretic activities of a number of new compounds with an imidazo[1,2-*a*]pyridine core have been convincingly demonstrated.^6^ In addition to pharmacological research, imidazo[1,2-*a*]pyridine derivatives have also shown highly fluorescent characteristics with high quantum yields.^7^ Therefore, their synthetic approaches have gained substantial attention in recent years.^5,8^

Recently, 3-aroylimidazo[1,2-*a*]pyridine derivatives have found promising applications in the development of pharmaceutical chemistry research such as anticancer and antimitotic agents (Fig. 1, compounds A, B, C).^9^ In fact, various approaches to prepare 3-aroylimidazo[1,2-*a*]pyridine derivatives have been reported.^9^ The first effort for direct coupling reaction of 2-aminopyridines with chalcones by Cu-catalysed aerobic oxidation was disclosed by Monir and coworkers.^10^ In 2014 Kaswan *et al.* reported a similar research on the Cu-catalysed synthesis of 3-aroylimidazo[1,2-*a*]pyridines from 2-aminopyridines with chalcones under air.^11^ Then, Nguyen *et al.* demonstrated a protocol for the aerobic coupling of 2-aminopyridines with chalcones using CuFe_2_O_4_ nanoparticles catalyst to give 3-aroylimidazo[1,2-*a*]pyridines in good yields.^12^ In this procedure, both iodine and oxygen must be used as oxidants for the success of this transformation. In 2015, Kaswan *et al.* also reported a method to prepare 3-aroylimidazo[1,2-*a*]pyridines by one-pot, three-component reaction of 2-aminopyridines, acetophenones, and benzaldehyde derivatives in the presence of CuCl_2_ catalyst under air.^13^ One year later, Xing and coworkers described a practical synthesis of 3-aroylimidazo[1,2-*a*]pyridines by Iodine-promoted oxidative coupling reaction of 2-aminopyridines and chalcones.^14^ From 2015, several reports in the synthesis of 3-aroylimidazo[1,2-*a*]pyridines based on the tandem Cu(i)-catalysed oxidative cyclization reactions of 2-aminopyridines and 1-phenyl-3-(aryl)prop-2-yn-1-ones *via N*-(2-pyridinyl) enaminone intermediates were also disclosed.^15^

**Scheme 1:**
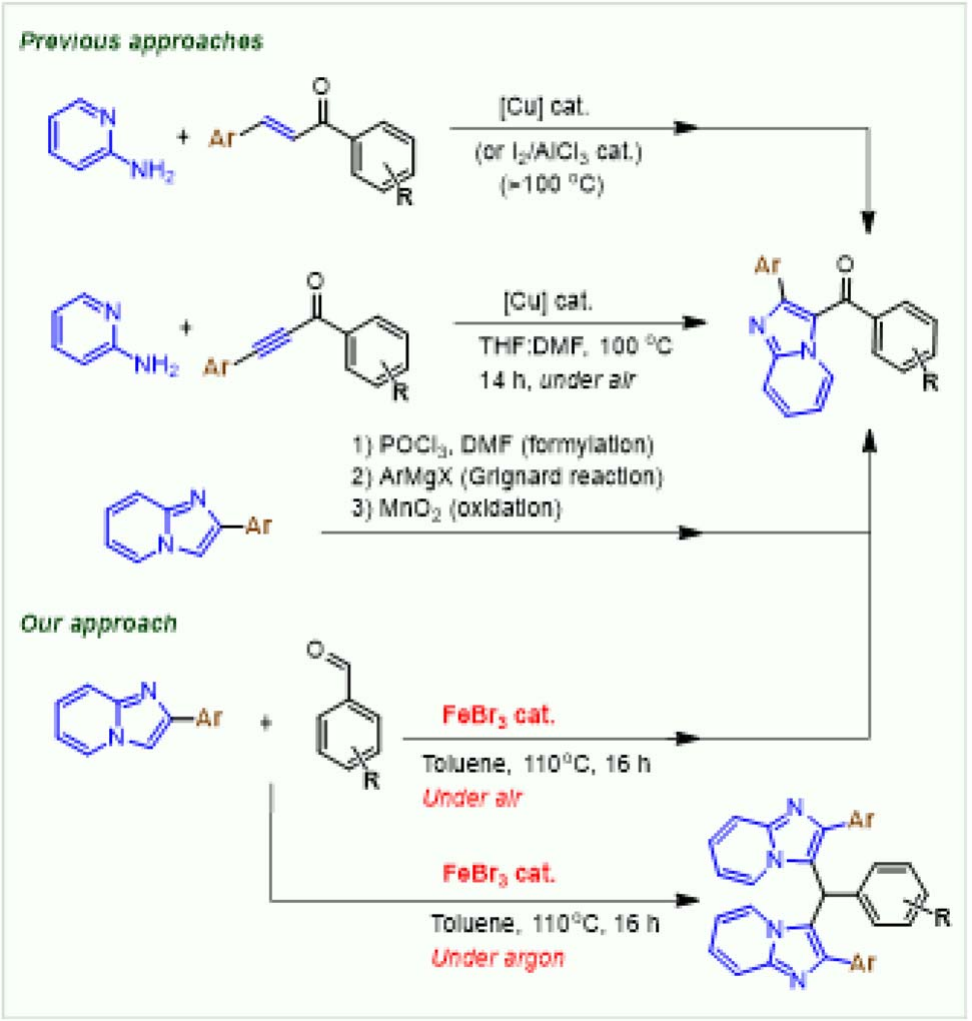
Several approaches to prepare 3-aroylimidazo[1,2-*a*]pyridines and 3,3′-(arylmethylene)bis(2-phenylimidazo[1,2-*a*]pyridines).

Recently, direct functionalization of imidazo[1,2-*a*]pyridines have been identified as an efficient approach to prepare bioactive imidazo[1,2-*a*]pyridine derivatives.^16^ In fact, the functionalization of imidazo[1,2-*a*]pyridines at the C3 position could not be directly made *via* the classical Friedel–Crafts acylation using aroyl chlorides and Lewis acid catalysts.^9^ Formal synthesis of 3-aroylimidazo[1,2-*a*]pyridine required a three-step procedure involving: (i) formylation of imidazo[1,2-*a*]pyridines, (ii) reaction of imidazo[1,2-*a*]pyridine-3-carbaldehydes with Grignard reagent to form secondary alcohols and (iii) oxidation of secondary alcohols to give 3-aroylimidazo[1,2-*a*]pyridines.^17^ In continuation of our efforts on the synthesis of imidazoheterocycles as well as indole-fused N-heterocycles,^18^ herein, we wish to report two practical procedures: (i) a direct FeBr_3_-catalysed functionalization of 2-arylimidazo[1,2-*a*]pyridine derivatives using aromatic aldehydes *via* aerobic oxidative cross-dehydrogenative coupling reaction; (ii) the FeBr_3_-catalysed alkylation of 2-arylimidazo[1,2-*a*]pyridine derivatives using aromatic aldehydes to give 3,3′-(arylmethylene)bis(2-phenylimidazo[1,2-*a*]pyridines) under argon atmosphere (Scheme 1).

## Results and discussion

The first step of this research was initiated by the preparation of 2-arylimidazo[1,2-*a*]pyridines as the key starting materials following a well-established method.^19^ In order to optimise this transformation, we choose the reaction between compound 1a and picolinaldehyde 2a as the representative reaction, as demonstrated in Table 1. With the purpose of regioselective functionalization of imidazo[1,2-*a*]pyridines at the C3 position, our attention was solely directed towards the investigation of various Lewis acid catalysts with the aim of identifying the optimal conditions for this transformation. Initially, the typical Lewis acids for Friedel–Crafts acylation was employed, resulting in a promising 60% yield of the desired product in the presence of FeCl_3_ catalyst (entries 1–4). The employment of several commonly used Fe(iii) salts as catalysts was examined which showed that FeBr_3_ could be the most suitable catalyst for this transformation (entries 5–9). Then, Fe(ii) salts were also examined as Lewis acid catalysts. However, we only obtained the desired product 3a in low yields (entries 10–12). Finally, further optimisations using different solvents, reaction time and temperatures did not give us better results (entries 13–19). In order to understand the real role of FeBr_3_ catalyst and the oxidant in the present study, two control experiments were carried out in the absence of catalyst and oxygen. In the absence of FeBr_3_ catalyst, only 9% of product 3a was obtained (entry 20). Notably, only 12% yield of product 3a was isolated from reaction mixture when this reaction was performed under argon atmosphere (entry 21). Hence, oxygen in air should be involved in the catalytic cycle as the oxidant.

**Table 1:** Optimization for the synthesis of 3-aroylimidazo[1,2-*a*]pyridine^a^

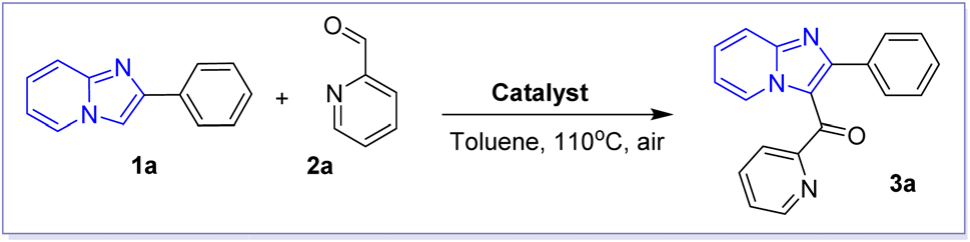					
Entry	Catalyst	Solvent	Time (h)	Temp. (°C)	Yield^b^ (%)
1	AlCl_3_	Toluene	16	110	—
2	ZrCl_4_	Toluene	16	110	—
3	CuCl_2_	Toluene	16	110	23
4	FeCl_3_	Toluene	16	110	60
5	FeBr_3_	Toluene	16	110	70
6	Fe_2_(SO_4_)_3_·9H_2_O	Toluene	16	110	—
7	Fe(NO_3_)_3_·9H_2_O	Toluene	16	110	—
8	Fe(acac)_3_	Toluene	16	110	—
9	Fe(OTf)_3_	Toluene	16	110	65
10	Fe(OAc)_2_·4H_2_O	Toluene	16	110	20
11	FeCl_2_	Toluene	16	110	15
12	FeSO_4_·7H_2_O	Toluene	16	110	2
13	FeBr_3_	DMF	16	110	10
14	FeBr_3_	1,4-Dioxane	16	110	5
15	FeBr_3_	Xylene	16	110	30
16	FeBr_3_	Toluene	16	120	65
17	FeBr_3_	Toluene	16	100	56
18	FeBr_3_	Toluene	24	110	72
19	FeBr_3_	Toluene	8	110	45
20	—	Toluene	16	110	9
21	FeBr_3_	Toluene	16	110	12^c^
22	FeBr_3_	Toluene	16	110	73^d^

a Condition: 1a (0.3 mmol), 2a (1.5 equiv.), [Fe] catalyst (20 mol%), 110 °C, 24 h.

b Yield of isolated products are given.

c Reaction was performed in argon atmosphere.

d Reaction was performed in oxygen atmosphere.

With the optimised condition in hand, we proceeded to investigate the potential application of this reaction of 2-arylimidazo[1,2-*a*]pyridines 1a–d with various aldehydes 2a–g, as described in Table 2. In general, the desired products were successfully prepared, resulting in isolated yields of up to 89%. Typically, the functionalization of 2-arylimidazo[1,2-*a*]pyridines with picolinaldehyde and isopicolinaldehydes frequently afforded to corresponding products 3a–h and 3o–r in high yields, reaching up to 89%. Interestingly, benzaldehyde and its derivatives could be successfully employed in the reaction with 2-phenylimidazo[1,2-*a*]pyridine 1a which only afforded to the corresponding products 3k–n in moderate yields.

**Table 2:** FeBr_3_-catalysed synthesis of 3-aroylimidazo[1,2-*a*]pyridine 3a–r^a^

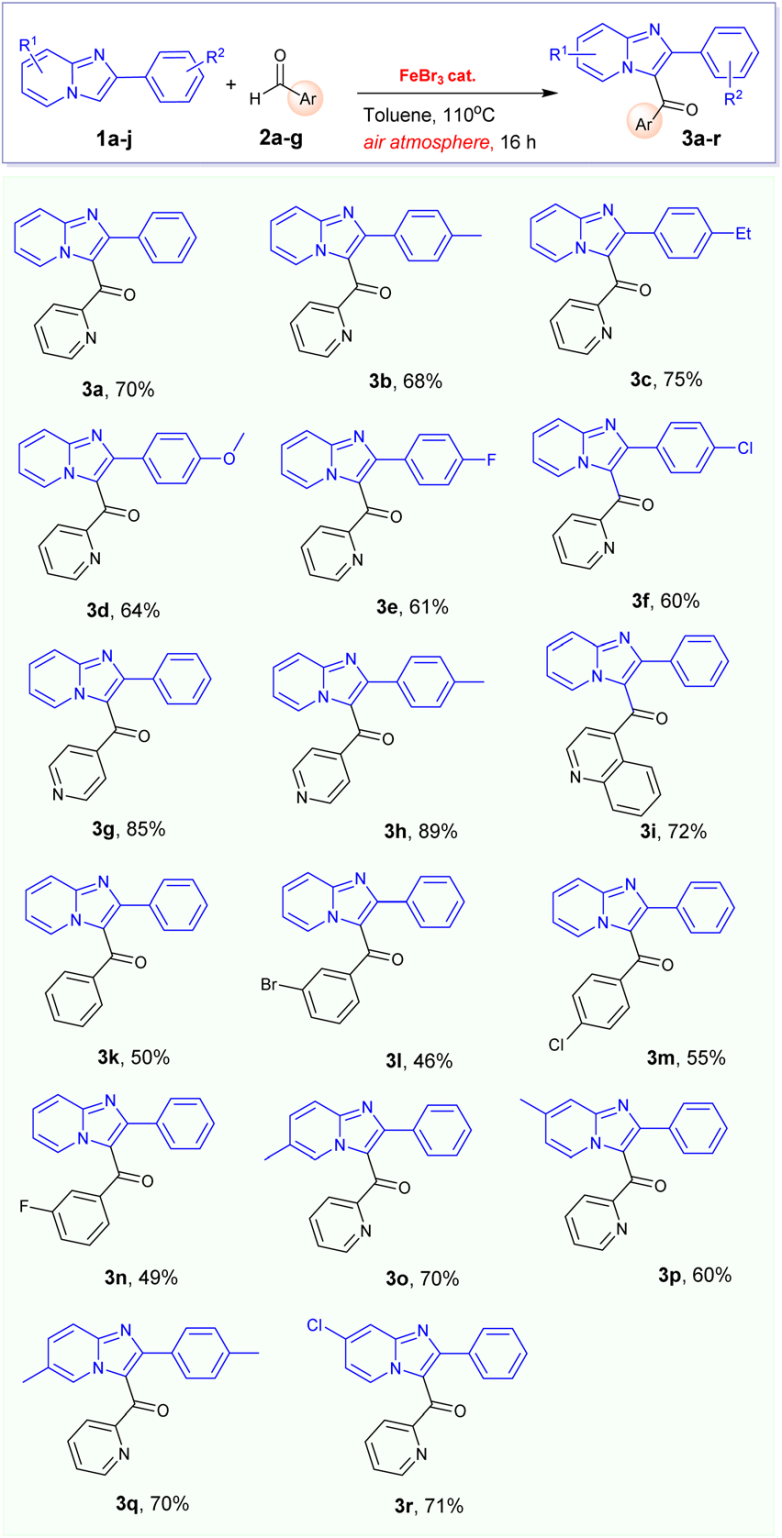

a Condition: 1a–d (0.5 mmol), 2a–g (1.5 equiv.), FeBr_3_ catalyst (20 mol%), 110 °C, 16 h; yields of isolated products are given.

Especially, the oxidative coupling reaction of phenylimidazo[1,2-*a*]pyridine 1a with 4-methylbenzaldehyde did not give the desired 3-aroylimidazo[1,2-*a*]pyridine under optimized condition. It is interesting to report that a new 3,3′-(*p*-tolylmethylene)bis(2-phenylimidazo[1,2-*a*]pyridine) 4b (Table 3) was formed in this reaction as the main product in 55% yield. This observation led us to conclude that the electrophilic activity of the aldehyde substrates plays a major role in the formation of products. In previous literature, only one example for the FeCl_3_-catalysed formation of product 4b was described in 2016 by Hajra's group.^16^ However, the authors did not clarify the reaction mechanism and the real role of oxygen. We consequently came to the conclusion that oxygen in air might be crucial to this transformation. In our effort to prepare the desired 3-aroylimidazo[1,2-*a*]pyridine product from 4-methylbenzaldehyde, this reaction was carried out under oxygen atmosphere. However, we also failed to obtain this product in reasonable yield. Remarkably, 3,3′-(*p*-tolylmethylene)bis(2-phenylimidazo[1,2-*a*]pyridine) product 4b can be prepared in improved yield (65%) when this reaction was carried out under argon atmosphere in the presence of FeBr_3_ catalyst. Consequently, we are interested in extending the scope of this transformation with other benzaldehyde derivatives. A series of 3,3′-(arylmethylene)bis(2-phenylimidazo[1,2-*a*]pyridines) 4a–e were prepared in good yields from the FeBr_3_-catalysed alkylation of phenylimidazo[1,2-*a*]pyridine 1a with benzaldehyde derivatives (Table 3).

**Table 3:** FeBr_3_-catalysed synthesis of 3,3′-(arylmethylene)bis(2-phenylimidazo[1,2-*a*]pyridines) 4a–e^a^

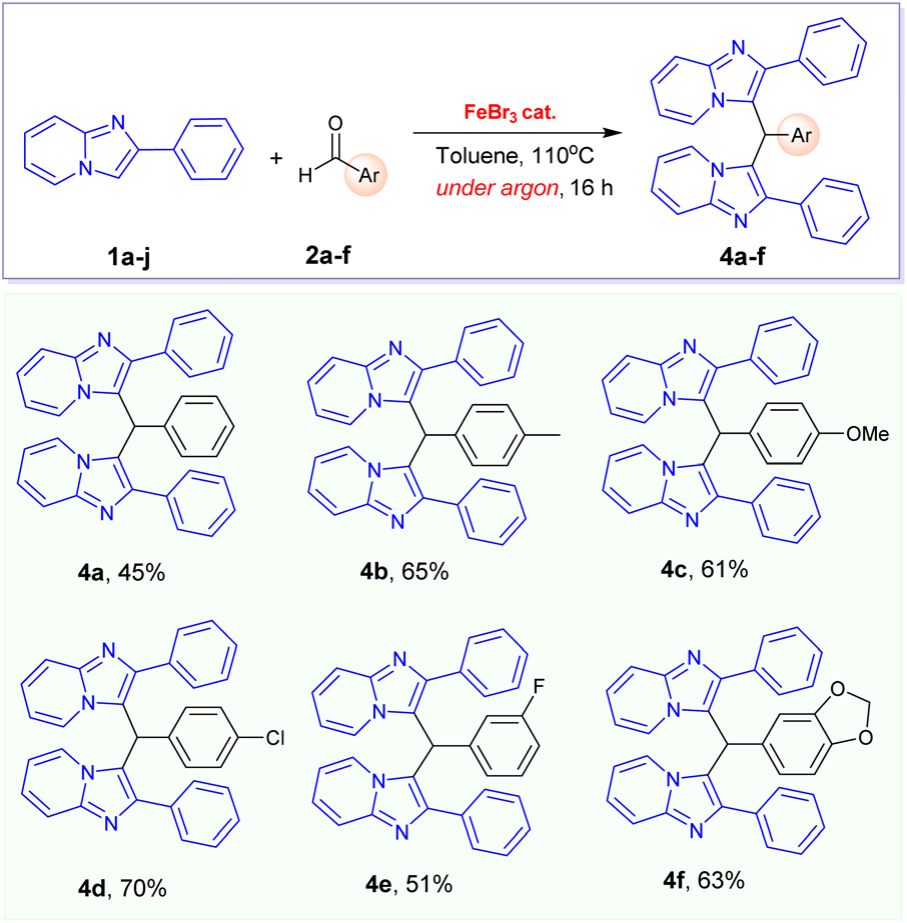

a Condition: 1a (0.515 mmol), 2a–g (1.0 equiv.), FeBr_3_ catalyst (20 mol%), 110 °C, under argon, 16 h; yields of isolated products are given.

Surprisingly, FeBr_3_-catalysed alkylation of phenylimidazo[1,2-*a*]pyridine 1a with hexanal did not give either 1-(2-phenylimidazo[1,2-*a*]pyridin-3-yl)hexan-1-one or 3,3′-(hexane-1,1-diyl)bis(2-phenylimidazo[1,2-*a*]pyridine) products under air or argon atmosphere conditions. Interestingly, only (*E*)-3-(hex-1-en-1-yl)-2-phenylimidazo[1,2-*a*]pyridine product 5a was obtained in 75% isolated yield (Scheme 5).

**Scheme 2:**
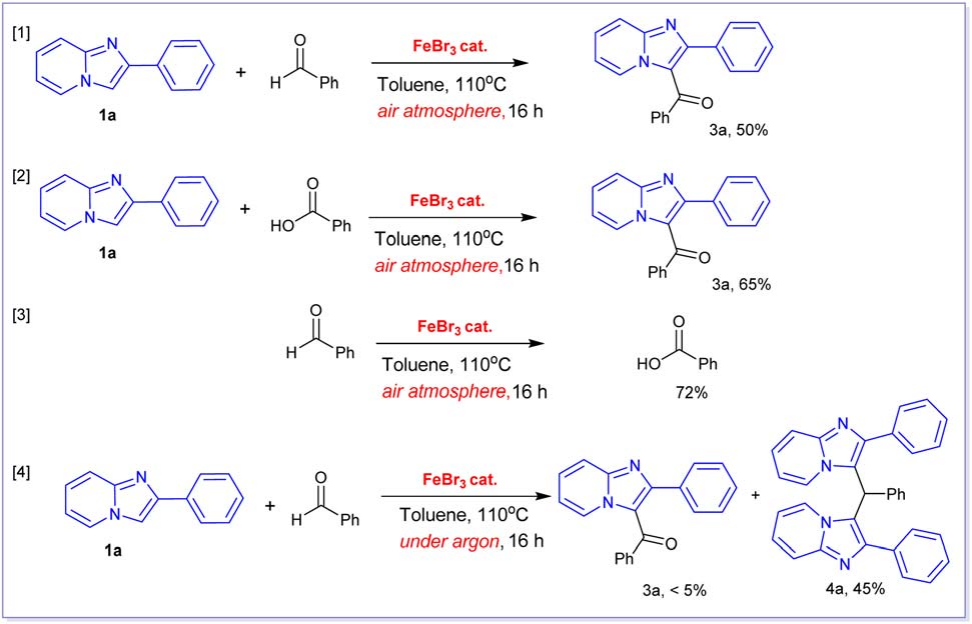
Control experiments.

We conducted several control experiments to better understand the mechanism underlying this transformation (Scheme 2). First, we discovered that the FeBr_3_-catalysed aerobic oxidative coupling reactions of 2-phenylimidazo[1,2-*a*]pyridine 1a with benzaldehyde and benzoic acid yielded the same product 3a in 50% and 65%, respectively (reactions [1], [2], Scheme 2). Based on these findings, we hypothesized that this transformation may happen in two steps. The first step would be the oxidation of benzaldehyde to produce benzoic acid in the presence of oxygen in air, followed by a second Friedel–Crafts-type alkylation reaction of 1a with *in situ*-formed benzoic acid to produce 3a. In order to confirm this hypothesis, the oxidation of benzaldehyde under optimized condition was performed which gave benzoic acid in 72% yield. This oxidation reaction was well established in literature.^20^ A very similar Fe(iii)-catalysed transformation of benzaldehyde to form benzoic acid using oxygen as oxidant was also reported by Wang and coworkers.^20^ Notably, the FeBr_3_-catalysed alkylation reaction of 2-phenylimidazo[1,2-*a*]pyridine 1a with benzaldehyde did not result in the formation of product 3a when this reaction was carried out under argon atmosphere (reaction [4]). Indeed, we obtained 3,3′-(phenylmethylene)bis(2-phenylimidazo[1,2-*a*]pyridine) product 4a in 45% isolated yield when was carried out under argon atmosphere in the employment of FeBr_3_ catalyst.

**Scheme 3:**
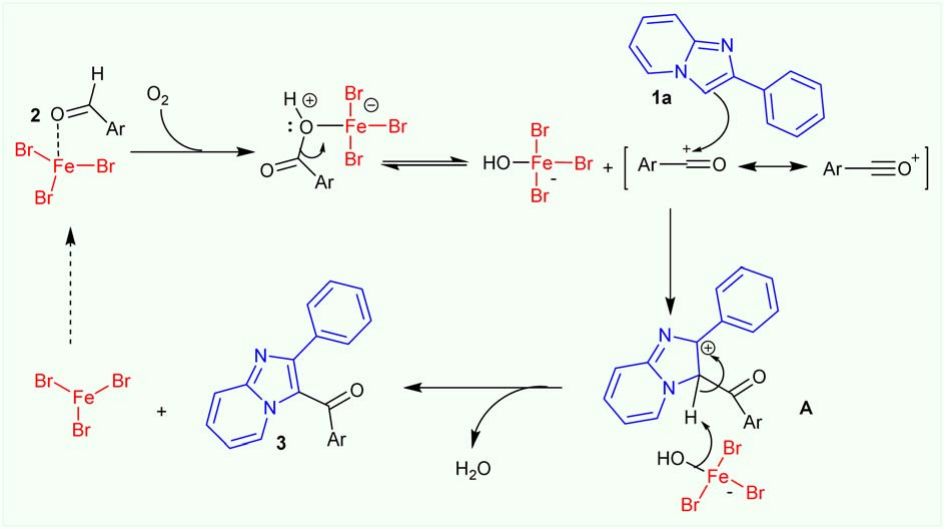
Plausible mechanism for the FeBr_3_-catalysed formation of 3-aroylimidazo[1,2-*a*]pyridines 3 in the presence of oxygen.

Based on the observed results in control experiments, a plausible mechanism for the FeBr_3_-catalysed synthesis of 3-aroylimidazo[1,2-*a*]pyridines from 2-arylimidazo[1,2-*a*]pyridine and aromatic aldehydes is proposed (Scheme 3). Firstly, the FeBr_3_-activated benzaldehyde may well react with oxygen in air to form a benzoic acid intermediate which subsequently react with 2-phenylimidazo[1,2-*a*]pyridine 1a*via* a Friedel–Crafts-type acylation reaction in the presence of FeBr_3_ catalyst. The *in situ*-formed intermediate A would be converted to 3-aroylimidazo[1,2-*a*]pyridine product 3 and regenerate the FeBr_3_ catalyst for the next catalytic cycle.

**Scheme 4:**
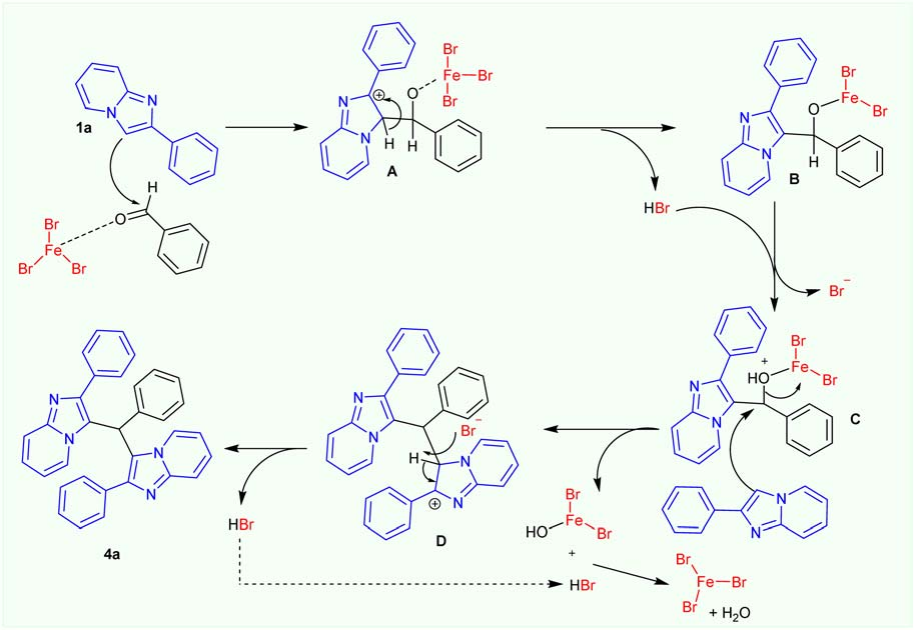
Possible mechanism for the FeBr_3_-catalysed formation of 3,3′-(arylmethylene)bis(2-phenylimidazo[1,2-*a*]pyridines) products under argon atmosphere.

Scheme 4 illustrates a possible process for the FeBr_3_ catalysed synthesis of 3,3′-(phenylmethylene)bis(2-phenylimidazo[1,2-*a*]pyridines) product 4. First, the nucleophilic attack of 2-phenylimidazo[1,2-*a*]pyridine 1a on FeBr_3_-activated benzaldehyde yielded the intermediate A, which was subsequently converted to intermediates B and C. Then, a second nucleophilic addition of 2-phenylimidazo[1,2-*a*]pyridine 1a to the intermediate C occurred to afford to the intermediate D. In order to produce 3,3′-(phenylmethylene)bis(2-phenylimidazo[1,2-*a*]pyridines) product 4a, the intermediate D may finally be deprotonated by a bromide anion. This will also remove a Fe(OH)Br_2_ molecule, which will then react with the *in situ*-formed HBr to regenerate FeBr_3_ catalyst for the subsequent catalytic cycle.

**Scheme 5:**
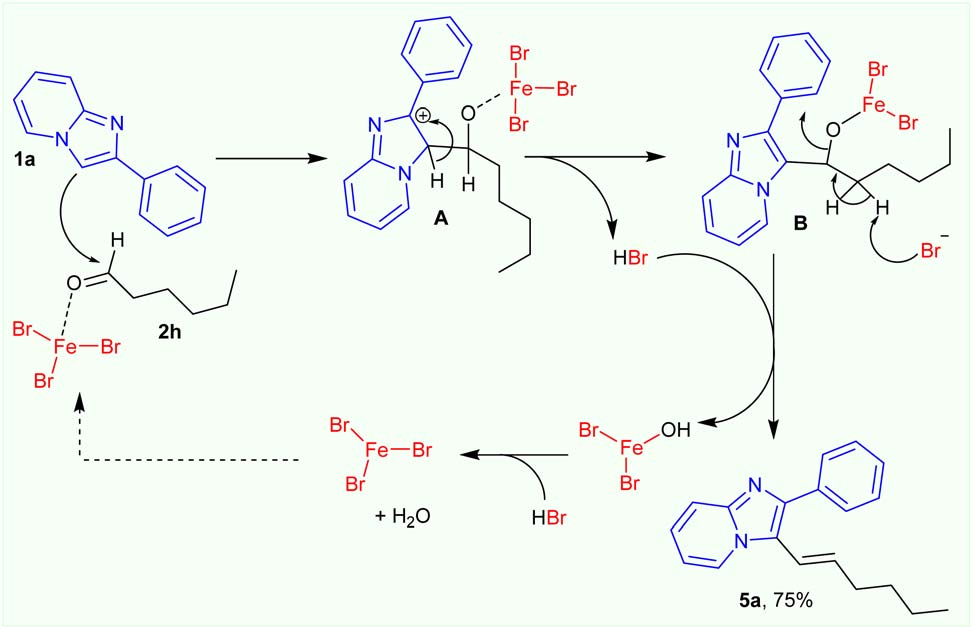
Plausible mechanism for the FeBr_3_-catalysed formation of (*E*)-3-(hex-1-en-1-yl)-2-phenylimidazo[1,2-*a*]pyridine product 5a.

As described above, FeBr_3_-catalysed alkylation of 2-phenylimidazo[1,2-*a*]pyridine 1a with hexanal resulted in the formation of only (*E*)-3-(hex-1-en-1-yl)-2-phenylimidazo[1,2-*a*]pyridine product 5a in 75% isolated yield. A plausible mechanism for the FeBr_3_-catalysed synthesis of (*E*)-3-(hex-1-en-1-yl)-2-phenylimidazo[1,2-*a*]pyridine product 5a from 2-phenylimidazo[1,2-*a*]pyridine and hexanal was proposed (Scheme 5). Firstly, 2-phenylimidazo[1,2-*a*]pyridine 1a react with FeBr_3_-activated hexanal to form similar proposed intermediates A, B. However, the *in situ*-formed bromide anion took β-proton from intermediate B to produce the alkene product 5a in 75% isolated yield.

## Conclusions

In conclusion, we are reporting a practical and convenient method for the synthesis of either 3-aroylimidazo[1,2-*a*]pyridines or 3,3′-(arylmethylene)bis(2-phenylimidazo[1,2-*a*]pyridines) derivatives from the same starting materials such as 2-arylimidazo[1,2-*a*]pyridines and aromatic aldehydes. To the best of our knowledge, this direct FeBr_3_-catalysed functionalization of 2-arylimidazo[1,2-*a*]pyridine derivatives using aryl aldehydes *via* aerobic oxidative cross-dehydrogenative coupling strategy has not been reported in the literature before. In addition, when this reaction was carried out under argon atmosphere, 3,3′-(arylmethylene)bis(2-phenylimidazo[1,2-*a*]pyridines) derivatives were chemoselectively formed in good yields. Plausible mechanisms for the selective formation of 3-aroylimidazo[1,2-*a*]pyridines or 3,3′-(arylmethylene)bis(2-phenylimidazo[1,2-*a*]pyridines) involving FeBr_3_ as a homogeneous catalyst have been proposed relying on observed experimental results. Remarkably, the role of oxygen was figured out to be the key oxidant for the chemoselective formation of 3-aroylimidazo[1,2-*a*]pyridines. The findings reported herein could be highly beneficial for the development of practical synthetic applications in materials science and medicinal chemistry. Currently, several biological activities investigations on 3-aroylimidazo[1,2-*a*]pyridine and 3,3′-(arylmethylene)bis(2-phenylimidazo[1,2-*a*]pyridines) derivatives are currently carried out in our laboratory.

## Experimental

### General procedure A for synthesis of compound 1a

In a round-bottom flask, 2-bromo-1-phenylethan-1-one (0.318 g, 1.6 mmol), 2-aminopyridine (0.181 g, 1.920 mmol), and sodium bicarbonate (0.134 g, 1.6 mmol) were dissolved in ethanol (3 mL). The reaction mixture was stirred at 70 °C for 6 hours. After cooling to room temperature, the solvent was removed under reduced pressure. The residue was extracted with ethyl acetate and water, and the organic layer was dried over anhydrous sodium sulfate. Evaporation of the solvent afforded the crude product, which was purified by column chromatography on silica gel using a hexane/ethyl acetate (3 : 1) solvent system to afford 2-phenylimidazo[1,2-*a*]pyridine 1a as a white solid (0.280 g, 90%).

^1^H NMR (600 MHz, CDCl_3_) *δ* 8.07 (dt, *J* = 6.7, 1.2 Hz, 1H), 7.97–7.93 (m, 2H), 7.82 (d, *J* = 0.8 Hz, 1H), 7.62 (dq, *J* = 9.0, 1.0 Hz, 1H), 7.45–7.40 (m, 2H), 7.34–7.30 (m, 1H), 7.14 (ddd, *J* = 9.1, 6.7, 1.3 Hz, 1H), 6.74 (td, *J* = 6.7, 1.2 Hz, 1H). ^13^C NMR (151 MHz, CDCl_3_) *δ* 145.8, 145.7, 133.8, 128.7, 127.9, 126.1, 125.6, 124.6, 117.6, 112.4, 108.1.

### General procedure B for synthesis of compound 3a

In a reaction tube, 2-phenylimidazo[1,2-*a*]pyridine 1a (100 mg, 0.515 mmol), pyridine-2-carbaldehyde 2a (83 mg, 0.772 mmol), and iron(iii) bromide (FeBr_3_) (30.4 mg, 0.103 mmol) were dissolved in toluene (0.6 mL). The reaction mixture was stirred at 110 °C for 16 hours. After this reaction completed, the reaction mixture was extracted with water and ethyl acetate. The organic layer was dried over anhydrous sodium sulfate, and then the solvent was removed under reduced pressure. The crude residue was purified by column chromatography on silica gel using a hexane/ethyl acetate (2 : 1) solvent system to afford (2-phenylimidazo[1,2-*a*]pyridin-3-yl)(pyridin-2-yl)methanone 3a as a white solid (108 mg, 70%).

^1^H NMR (600 MHz, CDCl_3_) *δ* 9.65 (dt, *J* = 6.9, 1.2 Hz, 1H), 8.09 (ddd, *J* = 4.8, 1.7, 0.9 Hz, 1H), 7.82 (dt, *J* = 8.9, 1.2 Hz, 1H), 7.69 (dt, *J* = 7.8, 1.1 Hz, 1H), 7.60 (td, *J* = 7.7, 1.7 Hz, 1H), 7.55 (ddd, *J* = 8.9, 6.9, 1.3 Hz, 1H), 7.32–7.27 (m, 2H), 7.16–7.04 (m, 5H). ^13^C NMR (151 MHz, CDCl_3_) *δ* 185.5, 156.8, 155.6, 148.4, 147.6, 136.2, 134.7, 129.7, 129.6, 128.5, 127.9, 127.7, 125.4, 123.8, 119.9, 117.5, 114.9.

### General procedure C for synthesis of compound 4a

In a reaction tube, 2-phenylimidazo[1,2-*a*]pyridine 1a (100 mg, 0.515 mmol), pyridine-2-carbaldehyde 2a (83 mg, 0.772 mmol), and iron(iii) bromide (FeBr_3_) (30.4 mg, 0.103 mmol) were dissolved in toluene (0.6 mL). Then, the reaction tube was backfilled with argon gas 3 times. The reaction mixture was stirred at 110 °C for 16 hours. After this reaction completed, the reaction mixture was extracted with water and ethyl acetate. The organic layer was dried over anhydrous sodium sulfate, and then the solvent was removed under reduced pressure. The crude residue was purified by column chromatography on silica gel using a hexane/ethyl acetate (2 : 1) solvent system to obtain 3,3′-(phenylmethylene)bis(2-phenylimidazo[1,2-*a*]pyridine) 4a as a brown solid (56 mg, 45%).

^1^H NMR (600 MHz, CDCl_3_) *δ* 7.60 (dt, *J* = 9.0, 1.1 Hz, 2H), 7.35–7.28 (m, 4H), 7.23 (dt, *J* = 6.9, 1.2 Hz, 2H), 7.21–7.15 (m, 3H), 7.15–7.05 (m, 7H), 6.86–6.76 (m, 2H), 6.53 (s, 1H), 6.41 (td, *J* = 6.8, 1.2 Hz, 2H). ^13^C NMR (151 MHz, CDCl_3_) *δ* 145.3, 144.7, 135.7, 133.8, 129.2, 128.7, 128.0, 127.9, 127.7, 127.7, 124.5, 124.1, 118.0, 117.3, 112.2, 38.5.

## Data availability

The datasets supporting this article have been uploaded as part of the ESI.† Data which are reported in this manuscript are available from the authors upon reasonable request.

## Conflicts of interest

There are no conflicts to declare.

## Supplementary Material

RA-014-D4RA05198J-s001
