# Supplementary material for: FeBr3-catalysed synthesis of 3-aroylimidazo[1,2-a]pyridine and 3,3′-(arylmethylene)bis(2-phenylimidazo[1,2-a]pyridines) derivatives from 2-arylimidazo[1,2-a]pyridines and aromatic aldehydes: an investigation about mechanistic insights
Source: RSC Adv. 2024 Sep 18;14(40):29535–41. doi: 10.1039/d4ra05198j (PMC11409445; doi:10.1039/d4ra05198j)
Supplement: RA-014-D4RA05198J-s001 [file RA-014-D4RA05198J-s001.pdf]

Supplementary Information (SI) for RSC Advances.
This journal is © The Royal Society of Chemistry 2024

Supporting Information

Synthesis of 3-aroylimidazo[1,2-*a*]pyridine derivatives by $FeBr_3$-catalysed C-H functionalization of 2-arylimidazo[1,2-*a*]pyridines under air

Tran Quang Hung,*[a,d] Ban Van Phuc,[a,§] Mai Phuong Nguyen,[b,§] Tuan Linh Tran,[b,§] Dang Van Do,[b] Ha Thanh Nguyen,[a,d] Van Tuyen Nguyen,[a,d] Hien Nguyen,[c] Tuan Thanh Dang*[b]

[a)] *Institute of Chemistry, Vietnam Academy of Science and Technology, Viet Nam.*
[b)] *Faculty of Chemistry, Hanoi University of Science, Vietnam National University (VNU), Viet Nam.*
[c)] *Faculty of Chemistry, Hanoi National University of Education (HNUE), Viet Nam.*
[d)] *Graduate University of Science and Technology, Vietnam Academy of Science and Technology, Viet Nam.*

CONTENT

1. **Experimental Section**
2. **Copies of NMR spectra**

1. Experimental Section

General procedure for preparation of 2-bromo-1-phenylethan-1-one and derivatives

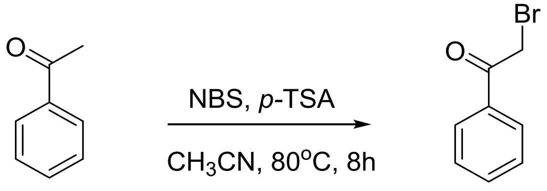

In a round-bottom flask, acetophenone (1 g, 8.32 mmol), *N*-bromosuccinimide (NBS) (1.481 g, 8.32 mmol), and *p*-toluenesulfonic acid (1.583 g, 8.32 mmol) were dissolved in acetonitrile (15 mL). The reaction mixture was stirred at 80°C for 8 hours. After cooling to room temperature, the volatiles were removed under reduced pressure. The residue was extracted with ethyl acetate and water, and the organic layer was dried over anhydrous sodium sulfate. Evaporation of the solvent gave **2-bromo-1-phenylethan-1-one** as a brown solid (1.640 g, 99%) [1]. The product is used for the next step without further purification.

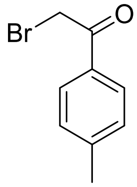

2-bromo-1-(p-tolyl)ethan-1-one prepared following general procedure A using 4-methyl acetophenone (1.116 g, 8.32 mmol), to yield 2-bromo-1-(p-tolyl)ethan-1-one (1.720 g, 97 %) as a brown solid. The product is used for the next step without further purification. [2]

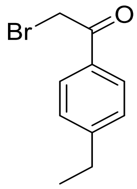

2-bromo-1-(4-ethylphenyl)ethan-1-one prepared following general procedure A using 4-ethyl acetophenone (1.233 g, 8.32 mmol), to yield 2-bromo-1-(4-ethylphenyl)ethan-1-one (1.833 g, 97 %) as a brown solid. The product is used for the next step without further purification.[3]

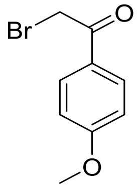

2-bromo-1-(4-methoxyphenyl)ethan-1-one prepared following general procedure A using 4-methoxy acetophenone (1.249 g, 8.32 mmol), to

yield 2-bromo-1-(4-methoxyphenyl)ethan-1-one (1.868 g, 98 %) as a brown solid. The product is used for the next step without further purification. [1]

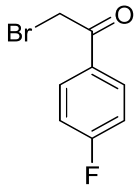

2-bromo-1-(4-fluorophenyl)ethan-1-one prepared following general procedure A using 4-fluoro acetophenone (1.149 g, 8.32 mmol), to yield 2-bromo-1-(4-fluorophenyl)ethan-1-one (1.788 g, 99 %) as a brown solid. The product is used for the next step without further purification.[4]

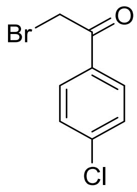

2-bromo-1-(4-chlorophenyl)ethan-1-one prepared following general procedure A using 4-chloro acetophenone (1.286 g, 8.32 mmol), to yield 2-bromo-1-(4-chlorophenyl)ethan-1-one (1.923 g, 99 %) as a brown solid. The product is used for the next step without further purification. [1]

General procedure for preparation of 2-phenylimidazo[1,2-a]pyridine 1a and derivatives

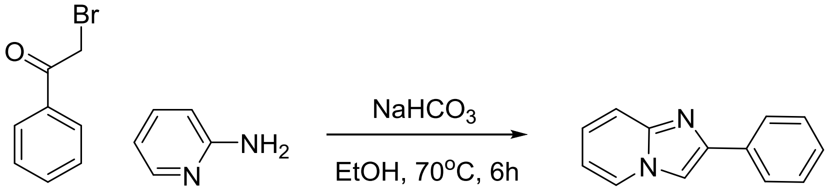

1a

In a round-bottom flask, 2-bromo-1-phenylethan-1-one (0.318 g, 1.6 mmol), 2-aminopyridine (0.181 g, 1.920 mmol), and sodium bicarbonate (0.134 g, 1.6 mmol) were dissolved in ethanol (3 mL). The reaction mixture was stirred at 70°C for 6 hours. After cooling to room temperature, the solvent was removed under reduced pressure. The residue was extracted with ethyl acetate and water, and the organic layer was dried over anhydrous sodium sulfate. Evaporation of the solvent afforded the crude product, which was purified by column chromatography on silica gel using a hexane/ethyl acetate (3:1) solvent system to afford 2-phenylimidazo[1,2-a]pyridine **1a** as a white solid (0.280 g, 90%). ^{1}H NMR (600 MHz, $CDCl_3$) δ 8.07 (dt, *J* = 6.7, 1.2 Hz, 1H), 7.97 – 7.93 (m, 2H), 7.82 (d, *J* = 0.8 Hz, 1H), 7.62 (dq, *J*

= 9.0, 1.0 Hz, 1H), 7.45 – 7.40 (m, 2H), 7.34 – 7.30 (m, 1H), 7.14 (ddd, *J* = 9.1, 6.7, 1.3 Hz, 1H), 6.74 (td, *J* = 6.7, 1.2 Hz, 1H). ^{13}C NMR (151 MHz, $CDCl_3$) δ 145.8, 145.6, 133.7, 128.7, 127.9, 126.0, 125.5, 124.6, 117.5, 112.4, 108.1. [5]

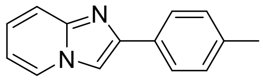

2-(p-tolyl)imidazo[1,2-a]pyridine **1b** prepared following general procedure B using 2-bromo-1-(p-tolyl)ethan-1-one (0.341 g, 1.6 mmol) and 2-aminopyridine (0.181 g, 1.920 mmol). The product was purified by column chromatography (silica gel, Hexane/ethylacetate 4:1) to yield **1b** (0.303 g, 91 %) as a white solid. ^{1}H NMR (600 MHz, $CDCl^3$) δ 8.07 – 8.04 (m, 1H), 7.86 – 7.82 (m, 2H), 7.79 – 7.77 (m, 1H), 7.63 – 7.59 (m, 1H), 7.24 (d, *J* = 7.8 Hz, 2H), 7.13 (dddd, *J* = 11.0, 8.4, 2.7, 1.2 Hz, 1H), 6.72 (tt, *J* = 5.2, 2.2 Hz, 1H), 2.38 (s, 3H). ^{13}C NMR (151 MHz, $CDCl_3$) δ 145.9, 145.6, 137.8, 130.9, 129.4, 125.9, 125.5, 124.4, 117.4, 112.2, 107.7, 21.2.[6]

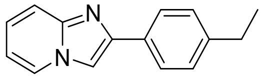

2-(4-ethylphenyl)imidazo[1,2-a]pyridine **1c** prepared following general procedure B using 2-bromo-1-(4-ethylphenyl)ethan-1-one (0.363 g, 1.6 mmol) and 2-aminopyridine (0.181 g, 1.920 mmol). The product was purified by column chromatography (silica gel, Hexane/ethylacetate 5:1) to yield **1c** (0.320 g, 90 %) as a white solid. ^{1}H NMR (600 MHz, $CDCl_3$) δ 8.07 (dt, *J* = 6.7, 1.2 Hz, 1H), 7.88 – 7.86 (m, 2H), 7.79 (d, *J* = 0.8 Hz, 1H), 7.61 (dq, *J* = 9.2, 1.0 Hz, 1H), 7.28 – 7.25 (m, 2H), 7.13 (ddd, *J* = 9.1, 6.7, 1.3 Hz, 1H), 6.73 (td, *J* = 6.7, 1.2 Hz, 1H), 2.68 (q, *J* = 7.6 Hz, 2H), 1.27 (t, *J* = 7.6 Hz, 3H). ^{13}C NMR (151 MHz, $CDCl^3$) δ 145.9, 145.6, 144.1, 131.1, 128.2, 126.0, 125.5, 124.4, 117.4, 112.2, 107.7, 28.6, 15.4.[7]

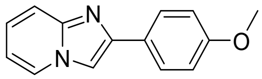

2-(4-methoxyphenyl)imidazo[1,2-a]pyridine **1d** prepared following general procedure B using 2-bromo-1-(4-methoxyphenyl)ethan-1-one (0.367 g, 1.6 mmol) and 2-aminopyridine (0.181 g, 1.920 mmol). The product was purified by column chromatography (silica gel, Hexane/ethylacetate 3:1) to yield **4d** (0.287 g, 80 %) as a white solid. ^{1}H NMR (600 MHz, $CDCl_3$) δ 8.02 (dt, *J* = 6.8, 1.2 Hz, 1H), 7.88 – 7.85 (m, 2H), 7.70 (d, *J* = 0.8 Hz, 1H), 7.59 (dq, *J* = 9.1, 1.0 Hz, 1H), 7.11 (ddd, *J* = 9.1, 6.7, 1.3 Hz, 1H),

6.97 – 6.93 (m, 2H), 6.69 (td, J = 6.7, 1.2 Hz, 1H), 3.82 (s, 3H). ^{13}C NMR (151 MHz, $CDCl_3$) δ 159.6, 145.6, 127.3, 126.5, 125.4, 124.4, 117.2, 114.1, 113.7, 112.1, 107.2, 55.2.[6]

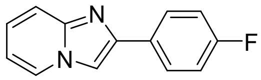

2-(4-fluorophenyl)imidazo[1,2-a]pyridine **1e** prepared following general procedure B using 2-bromo-1-(4-fluorophenyl)ethan-1-one (0.347 g, 1.6 mmol) and 2-aminopyridine (0.181 g, 1.920 mmol). The product was purified by column chromatography (silica gel, Hexane/ethylacetate 4:1) to yield **1e** (0.265 g, 78 %) as a white solid. ^{1}H NMR (600 MHz, $CDCl_3$) δ 8.09 – 8.05 (m, 1H), 7.93 – 7.88 (m, 2H), 7.77 (dd, J = 4.6, 2.4 Hz, 1H), 7.61 (d, J = 9.1 Hz, 1H), 7.18 – 7.08 (m, 3H), 6.77 – 6.72 (m, 1H). ^{13}C NMR (151 MHz, $CDCl_3$) δ 162.7 (d, J = 247.0 Hz), 145.6, 144.9, 130.0 (d, J = 3.2 Hz), 127.7 (d, J = 8.0 Hz), 125.5, 124.7, 117.4, 115.6 (d, J = 21.7 Hz), 112.4, 107.7.[6]

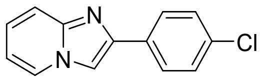

2-(4-chlorophenyl)imidazo[1,2-a]pyridine **1f** prepared following general procedure B using 2-bromo-1-(4-chlorophenyl)ethan-1-one (0.374 g, 1.6 mmol) and 2-aminopyridine (0.181 g, 1.920 mmol). The product was purified by column chromatography (silica gel, Hexane/ethylacetate 4:1) to yield **1f** (0.274 g, 75 %) as a white solid. ^{1}H NMR (600 MHz, $CDCl_3$) δ 8.05 (dt, J = 6.7, 1.2 Hz, 1H), 7.88 – 7.83 (m, 2H), 7.78 (s, 1H), 7.63 – 7.59 (m, 1H), 7.40 – 7.35 (m, 2H), 7.15 (ddd, J = 9.1, 6.7, 1.3 Hz, 1H), 6.75 (td, J = 6.8, 1.2 Hz, 1H).^{13}C NMR (151 MHz, $CDCl_3$) δ 145.7, 144.6, 133.6, 132.3, 128.8, 127.2, 125.6, 124.9, 117.4, 112.5, 108.2.[6]

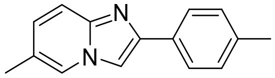

6-methyl-2-(p-tolyl)imidazo[1,2-a]pyridine **1g** prepared following general procedure B using 2-bromo-1-(p-tolyl)ethan-1-one (0.341 g, 1.6 mmol) and 5-methylpyridin-2-amine (0.208 g, 1.920 mmol). The product was purified by column chromatography (silica gel, Hexane/ethylacetate 4:1) to yield **1g** (0.285 g, 80 %) as a white solid. ^{1}H NMR (600 MHz, $CDCl_3$) δ 7.82 (d, J = 8.2 Hz, 3H), 7.68 (s, 1H), 7.50 (d, J = 9.2 Hz, 1H), 7.22 (d, J = 7.8 Hz, 2H), 6.97 (dd, J = 9.3, 1.7 Hz, 1H), 2.37 (s, 3H), 2.29 –

2.26 (m, 3H).^{13}C NMR (151 MHz, $CDCl_3$) δ 145.5, 144.6, 137.5, 131.0, 129.3, 127.6, 125.8, 123.2, 121.8, 116.7, 107.5, 21.2, 18.0.[6]

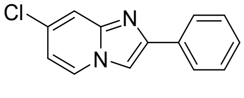

7-chloro-2-phenylimidazo[1,2-a]pyridine **1h** prepared following general procedure B using 2-bromo-1-phenylethan-1-one (0.318 g, 1.6 mmol) and 4-chloropyridin-2-amine (0.247 g, 1.920 mmol). The product was purified by column chromatography (silica gel, Hexane/ethylacetate 4:1) to yield **1h** (0.256 g, 70 %) as a white solid. ^{1}H NMR (600 MHz, $CDCl_3$) δ 7.98 (dd, *J* = 7.1, 0.8 Hz, 1H), 7.92 – 7.89 (m, 2H), 7.77 (d, *J* = 0.8 Hz, 1H), 7.61 (dt, *J* = 1.8, 0.8 Hz, 1H), 7.45 – 7.40 (m, 2H), 7.35 – 7.31 (m, 1H), 6.73 (dd, *J* = 7.2, 2.1 Hz, 1H).^{13}C NMR (151 MHz, $CDCl_3$) δ 146.8, 145.4, 133.2, 131.0, 128.7, 128.2, 126.0, 125.7, 116.3, 114.0, 108.2.[8]

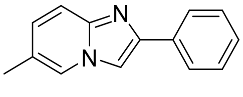

6-methyl-2-phenylimidazo[1,2-a]pyridine **1i** prepared following general procedure B using 2-bromo-1-phenylethan-1-one (0.318 g, 1.6 mmol) and 5-methylpyridin-2-amine (0.208 g, 1.920 mmol). The product was purified by column chromatography (silica gel, Hexane/ethylacetate 4:1) to yield **1i** (0.283 g, 85 %) as a white solid. ^{1}H NMR (600 MHz, $CDCl_3$) δ 7.91 – 7.87 (m, 2H), 7.67 (q, *J* = 1.4 Hz, 1H), 7.59 (s, 1H), 7.46 (d, *J* = 9.2 Hz, 1H), 7.38 (dd, *J* = 8.4, 7.1 Hz, 2H), 7.29 – 7.26 (m, 1H), 6.90 (dd, *J* = 9.2, 1.8 Hz, 1H), 2.17 (d, *J* = 1.5 Hz, 3H). ^{13}C NMR (151 MHz, $CDCl_3$) δ 145.1, 144.5, 133.8, 128.4, 127.5, 127.5, 125.7, 123.1, 121.7, 116.4, 107.7, 17.7.[6]

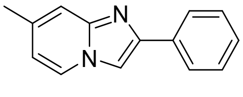

7-methyl-2-phenylimidazo[1,2-a]pyridine **1j** prepared following general procedure B using 2-bromo-1-phenylethan-1-one (0.318 g, 1.6 mmol) and 4-chloropyridin-2-amine (0.208 g, 1.920 mmol). The product was purified by column chromatography (silica gel, Hexane/ethylacetate 4:1) to yield **1j** (0.280 g, 84 %) as a white solid. ^{1}H NMR (600 MHz, $CDCl_3$) δ 7.93 – 7.90 (m, 2H), 7.86 (dd, *J* = 6.9, 1.0 Hz, 1H), 7.67 (d, *J* = 0.8 Hz, 1H), 7.40 (ddt, *J* = 7.5, 6.4, 1.1 Hz, 2H), 7.34 (dq, *J* = 1.9, 1.0 Hz, 1H), 7.29 (ddt, *J* = 8.6, 6.9, 1.3 Hz, 1H), 6.50 (dd, *J* = 6.9, 1.7 Hz, 1H), 2.33 (d, *J* = 1.4 Hz, 3H). ^{13}C NMR (151 MHz,

$CDCl_3$) δ 146.1, 145.4, 135.5, 134.0, 128.6, 127.7, 125.9, 124.7, 115.8, 114.9, 107.5, 21.3.[9]

General procedure B for prepared of (2-phenylimidazo[1,2-a]pyridin-3-yl)(pyridin-2-yl)methanone 3a and derivatives

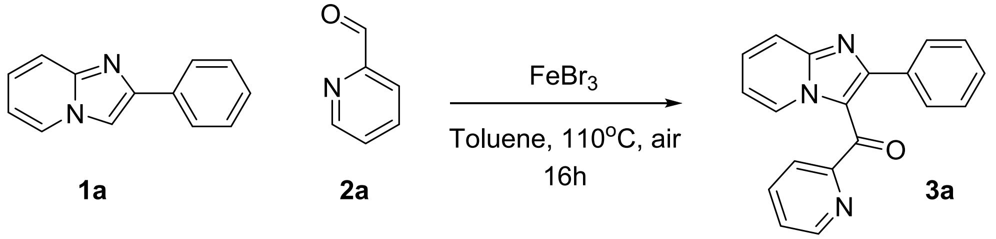

In a reaction tube, 2-phenylimidazo[1,2-a]pyridine **1a** (100 mg, 0.515 mmol), pyridine-2-carbaldehyde **2a** (83 mg, 0.772 mmol), and iron(III) bromide (FeBr3) (30.4 mg, 0.103 mmol) were dissolved in toluene (0.6 mL). The reaction tube was tightly capped and the reaction mixture was stirred at 110°C for 16 hours. The reaction mixture was then extracted with water and ethyl acetate. The organic layer was dried over anhydrous sodium sulfate, and the solvent was removed under reduced pressure. The crude residue was purified by column chromatography on silica gel using a hexane/ethyl acetate (2:1) solvent system to afford (2-phenylimidazo[1,2-a]pyridin-3-yl)(pyridin-2-yl)methanone **3a** as a white solid (108 mg, 70%). ^{1}H NMR (600 MHz, $CDCl_3$) δ 9.65 (dt, J = 6.9, 1.2 Hz, 1H), 8.09 (ddd, J = 4.8, 1.7, 0.9 Hz, 1H), 7.82 (dt, J = 8.9, 1.2 Hz, 1H), 7.69 (dt, J = 7.8, 1.1 Hz, 1H), 7.60 (td, J = 7.7, 1.7 Hz, 1H), 7.55 (ddd, J = 8.9, 6.9, 1.3 Hz, 1H), 7.32 – 7.27 (m, 2H), 7.16 – 7.04 (m, 5H). ^{13}C NMR (151 MHz, $CDCl_3$) δ 185.5, 156.7, 155.6, 148.4, 147.6, 136.1, 134.7, 129.7, 129.6, 128.4, 127.9, 127.6, 125.3, 123.8, 119.9, 117.4, 114.9. [10]

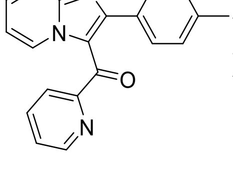

pyridin-2-yl(2-(p-tolyl)imidazo[1,2-a]pyridin-3-yl)methanone **3b** prepared following general procedure C using compound **1b** (108 mg, 0.515 mmol) and pyridine-2-carbaldehyde **2a** (83 mg, 0.772 mmol). The product was purified by column chromatography (silica gel, Hexane/ethylacetate 2:1) to yield **3b** (110 mg, 68 %) as a white solid.

^{1}H NMR (600 MHz, $CDCl_3$) δ 9.63 (dt, *J* = 6.9, 1.2 Hz, 1H), 8.13 (ddd, *J* = 4.8, 1.7, 1.0 Hz, 1H), 7.80 (dt, *J* = 8.9, 1.2 Hz, 1H), 7.67 (dt, *J* = 7.8, 1.1 Hz, 1H), 7.59 (td, *J* = 7.7, 1.7 Hz, 1H), 7.54 (ddd, *J* = 8.9, 6.9, 1.3 Hz, 1H), 7.20 – 7.16 (m, 2H), 7.13 – 7.08 (m, 2H), 6.89 – 6.85 (m, 2H), 2.23 (s, 3H). ^{13}C NMR (151 MHz, $CDCl_3$) δ 185.5, 156.9, 155.7, 148.4, 147.6, 137.8, 136.1, 131.7, 129.6, 129.5, 128.4, 128.3, 125.1, 123.9, 119.8, 117.3, 114.7, 21.1. HRMS (ESI) m/z: $[M+H]^+$ Calcd for $C_{20}H_{15}N_3O$ 314.1287; found: 314.1276.

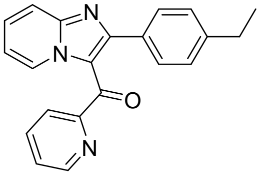

(2-(4-ethylphenyl)imidazo[1,2-a]pyridin-3-yl)(pyridin-2-yl)methanone **3c** prepared following general procedure C using compound **1c** (115 mg, 0.515 mmol) and pyridine-2-carbaldehyde **2a** (83 mg, 0.772 mmol). The product was purified by column chromatography (silica gel, Hexane/ethylacetate 2:1) to yield **3c** (127 mg, 75 %) as a white solid. ^{1}H NMR (600 MHz, $CDCl_3$) δ 9.65 (dt, *J* = 6.9, 1.2 Hz, 1H), 8.11 – 8.10 (m, 1H), 7.79 (dt, *J* = 8.9, 1.2 Hz, 1H), 7.64 (dt, *J* = 7.8, 1.1 Hz, 1H), 7.58 – 7.51 (m, 2H), 7.22 – 7.16 (m, 2H), 7.12 – 7.05 (m, 2H), 6.90 – 6.86 (m, 2H), 2.52 (q, *J* = 7.6 Hz, 2H), 1.13 (t, *J* = 7.7 Hz, 3H). ^{13}C NMR (151 MHz, $CDCl_3$) δ 185.4, 157.0, 155.7, 148.4, 147.6, 144.1, 136.0, 131.9, 129.7, 129.5, 128.4, 127.1, 125.1, 123.8, 117.3, 114.7, 28.5, 15.6. HRMS (ESI) m/z: $[M+H]^+$ Calcd for $C_{21}H_{17}N_3O$ 328.1444; found: 328.1432.

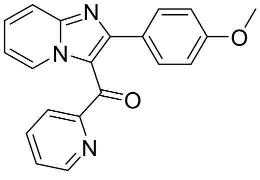

(2-(4-methoxyphenyl)imidazo[1,2-a]pyridin-3-yl)(pyridin-2-yl)methanone **3d** prepared following general procedure C using compound **1d** (116 mg, 0.515 mmol) and pyridine-2-carbaldehyde **2a** (83 mg, 0.772 mmol). The product was purified by column chromatography (silica gel, Hexane/ethylacetate 2:1) to yield **3d** (109 mg, 64 %) as a white solid. ^{1}H NMR (600 MHz, $CDCl_3$) δ 9.64 (dt, *J* = 6.9, 1.2 Hz, 1H), 8.18 (ddd, *J* = 4.8, 1.7, 1.0 Hz, 1H), 7.79 (dt, *J* = 8.9, 1.2 Hz, 1H), 7.67 (dt, *J* = 7.8, 1.1 Hz, 1H), 7.61 (td, *J* = 7.7, 1.7 Hz, 1H), 7.54 (ddd, *J* = 8.9, 6.9, 1.3 Hz, 1H), 7.25 – 7.21 (m, 2H), 7.15 – 7.09 (m, 2H), 6.62 – 6.59 (m, 2H), 3.73 (s, 3H). ^{13}C NMR (151 MHz, $CDCl_3$) δ 185.4, 159.5, 156.5, 155.8, 148.5, 147.7,

136.2, 131.0, 129.5, 128.4, 127.1, 125.2, 123.9, 119.7, 117.3, 114.7, 113.2, 55.2. HRMS (ESI) m/z: $[M+H]^+$ Calcd for $C_{20}H_{15}N_3O_2$ 330.1237; found: 330.1225.

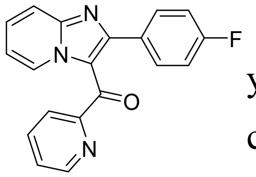

(2-(4-fluorophenyl)imidazo[1,2-a]pyridin-3-yl)(pyridin-2-yl)methanone **3e** prepared following general procedure C using compound **1e** (110 mg, 0.515 mmol) and pyridine-2-carbaldehyde **2a** (83 mg, 0.772 mmol). The product was purified by column chromatography (silica gel, Hexane/ethylacetate 2:1) to yield **3e** (100 mg, 61 %) as a white solid. ^{1}H NMR (600 MHz, $CDCl_3$) δ 9.63 (dt, J = 6.9, 1.2 Hz, 1H), 8.13 (ddd, J = 4.8, 1.7, 0.9 Hz, 1H), 7.80 (dt, J = 8.9, 1.2 Hz, 1H), 7.71 (dt, J = 7.8, 1.1 Hz, 1H), 7.65 (td, J = 7.7, 1.7 Hz, 1H), 7.55 (ddd, J = 8.9, 6.9, 1.3 Hz, 1H), 7.28 (ddd, J = 8.8, 4.8, 2.2 Hz, 2H), 7.16 (ddd, J = 7.6, 4.8, 1.3 Hz, 1H), 7.13 (td, J = 6.9, 1.3 Hz, 1H), 6.82 – 6.74 (m, 2H). ^{13}C NMR (151 MHz, $CDCl_3$) δ 185.3, 162.4 (d, J = 248.4 Hz), 155.5, 148.4, 147.6, 136.3, 131.4 (d, J = 8.4 Hz), 131.0 (d, J = 3.4 Hz), 129.7, 128.4, 125.5, 123.8, 119.9, 117.4, 115.0, 114.7 (d, J = 21.8 Hz). ^{19}F NMR (565 MHz, $CDCl_3$) δ -113.3. HRMS (ESI) m/z: $[M+H]^+$ Calcd for $C_{19}H_{12}FN_3O$ 318.1037; found: 318.1035.

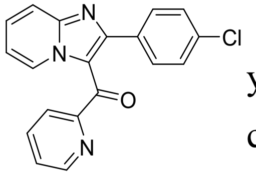

(2-(4-chlorophenyl)imidazo[1,2-a]pyridin-3-yl)(pyridin-2-yl)methanone **3f** prepared following general procedure C using compound **1f** (118 mg, 0.515 mmol) and pyridine-2-carbaldehyde **2a** (83 mg, 0.772 mmol). The product was purified by column chromatography (silica gel, Hexane/ethylacetate 2:1) to yield **3f** (100 mg, 60 %) as a white solid.[10] ^{1}H NMR (600 MHz, $CDCl_3$) δ 9.61 (dt, J = 6.9, 1.2 Hz, 1H), 8.11 (ddd, J = 4.8, 1.7, 1.0 Hz, 1H), 7.81 (dt, J = 9.0, 1.2 Hz, 1H), 7.73 (dt, J = 7.8, 1.1 Hz, 1H), 7.66 (td, J = 7.7, 1.7 Hz, 1H), 7.55 (ddd, J = 9.0, 6.9, 1.3 Hz, 1H), 7.25 – 7.21 (m, 2H), 7.18 (ddd, J = 7.6, 4.8, 1.3 Hz, 1H), 7.13 (td, J = 6.9, 1.3 Hz, 1H), 7.08 – 7.03 (m, 2H). ^{13}C NMR (151 MHz, $CDCl_3$) δ 185.2, 155.4, 155.3, 148.4, 147.6, 136.4, 134.1, 133.4, 130.9, 129.7, 128.4, 127.8, 125.5, 123.9, 119.9, 117.4, 115.0.

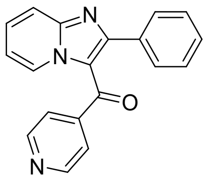

(2-phenylimidazo[1,2-a]pyridin-3-yl)(pyridin-4-yl)methanone **3g** prepared following general procedure C using compound **1a** (100 mg, 0.515 mmol) and isonicotinaldehyde **2b** (83 mg, 0.772 mmol). The product was purified by column chromatography (silica gel, Hexane/ethylacetate 1:1) to yield **3g** (131 mg, 85 %) as a white solid. ^{1}H NMR (600 MHz, $CDCl_3$) δ 9.66 (dt, *J* = 6.9, 1.2 Hz, 1H), 8.40 – 8.34 (m, 2H), 7.83 (dt, *J* = 8.9, 1.2 Hz, 1H), 7.59 (ddd, *J* = 8.9, 6.9, 1.3 Hz, 1H), 7.29 – 7.23 (m, 4H), 7.21 – 7.14 (m, 2H), 7.12 – 7.08 (m, 2H). ^{13}C NMR (151 MHz, $CDCl_3$) δ 185.0, 156.4, 149.5, 147.8, 145.6, 133.5, 130.2, 130.1, 129.0, 128.5, 127.9, 122.6, 119.7, 117.5, 115.2. HRMS (ESI) m/z: $[M+H]^+$ Calcd for $C_{19}H_{13}N_3O$ 300.1131; found: 300.1123.

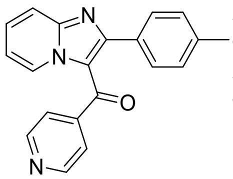

pyridin-4-yl(2-(p-tolyl)imidazo[1,2-a]pyridin-3-yl)methanone **3h** prepared following general procedure C using compound **1b** (108 mg, 0.515 mmol) and isonicotinaldehyde **2b** (83 mg, 0.772 mmol). The product was purified by column chromatography (silica gel, Hexane/ethylacetate 1:1) to yield **3h** (144 mg, 89 %) as a white solid. ^{1}H NMR (600 MHz, $CDCl_3$) δ 9.64 (dt, *J* = 6.9, 1.2 Hz, 1H), 8.39 – 8.36 (m, 2H), 7.81 (dt, *J* = 8.9, 1.2 Hz, 1H), 7.58 (ddd, *J* = 8.9, 6.9, 1.3 Hz, 1H), 7.26 – 7.24 (m, 2H), 7.16 – 7.12 (m, 3H), 6.91 – 6.89 (m, 2H), 2.24 (s, 3H). ^{13}C NMR (151 MHz, $CDCl_3$) δ 185.1, 156.6, 149.5, 147.8, 145.8, 139.1, 130.6, 130.1, 130.0, 128.6, 128.5, 122.7, 119.6, 117.5, 115.1, 21.2. HRMS (ESI) m/z: $[M+H]^+$ Calcd for $C_{20}H_{15}N_3O$ 314.1287; found: 314.1279.

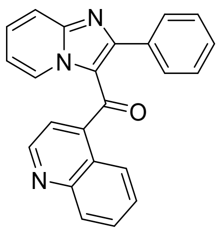

(2-phenylimidazo[1,2-a]pyridin-3-yl)(quinolin-4-yl)methanone **3i** prepared following general procedure C using compound **1a** (100 mg, 0.515 mmol) and quinoline-4-carbaldehyde **2c** (113 mg, 0.772 mmol). The product was purified by column chromatography (silica gel, Hexane/ethylacetate 1:1) to yield **3i** (126 mg, 72 %) as a white solid. ^{1}H NMR (600 MHz, $CDCl_3$) δ 9.98 (dt, *J* = 6.9, 1.2 Hz, 1H), 8.48 (d, *J* = 4.3 Hz, 1H), 8.06 (ddd, *J* = 8.4, 1.4, 0.7 Hz, 1H), 8.03 – 8.00 (m, 1H), 7.86 (dt, *J* =

8.9, 1.2 Hz, 1H), 7.71 (ddd, J = 8.4, 6.8, 1.4 Hz, 1H), 7.67 – 7.63 (m, 1H), 7.56 (ddd, J = 8.2, 6.8, 1.3 Hz, 1H), 7.24 (td, J = 6.9, 1.3 Hz, 1H), 7.02 (d, J = 4.3 Hz, 1H), 6.99 – 6.93 (m, 3H), 6.74 (ddt, J = 8.8, 7.7, 1.2 Hz, 2H). ^{13}C NMR (151 MHz, $CDCl_3$) δ 185.0, 157.9, 148.9, 148.1, 147.9, 144.3, 133.4, 130.6, 129.7, 129.5, 129.2, 129.0, 128.7, 127.5, 127.1, 125.3, 124.7, 121.0, 120.2, 117.6, 115.6. HRMS (ESI) m/z: $[M+H]^+$ Calcd for $C_{23}H_{15}N_3O$ 350.1287; found: 350.1272.

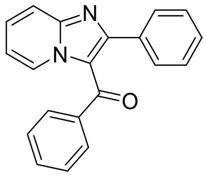

phenyl(2-phenylimidazo[1,2-a]pyridin-3-yl)methanone **3k** prepared following general procedure C using compound **1a** (100 mg, 0.515 mmol) and benzaldehyde **2d** (82 mg, 0.772 mmol). The product was purified by column chromatography (silica gel, Hexane/ethylacetate 3:1) to yield **3k** (77 mg, 50 %) as a brown solid. ^{1}H NMR (600 MHz, $CDCl_3$) δ 9.54 (dt, J = 7.0, 1.2 Hz, 1H), 7.87 – 7.82 (m, 1H), 7.53 – 7.49 (m, 3H), 7.44 (dddd, J = 8.2, 6.5, 1.8, 0.8 Hz, 1H), 7.35 – 7.31 (m, 2H), 7.27 – 7.23 (m, 1H), 7.15 – 7.05 (m, 5H). ^{13}C NMR (151 MHz, $CDCl_3$) δ 187.4, 154.8, 147.3, 138.6, 133.8, 131.7, 130.2, 130.0, 129.5, 129.2, 128.3, 128.2, 127.7, 120.0, 117.4, 114.6. [11]

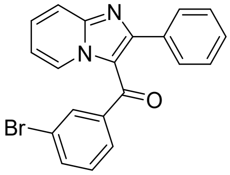

(3-bromophenyl)(2-phenylimidazo[1,2-a]pyridin-3-yl)methanone **3l** prepared following general procedure C using compound **1a** (100 mg, 0.515 mmol) and 3-bromobenzaldehyde **2e** (143 mg, 0.772 mmol). The product was purified by column chromatography (silica gel, Hexane/ethylacetate 3:1) to yield **3l** (89 mg, 46 %) as a brown solid. ^{1}H NMR (600 MHz, $CDCl_3$) δ 9.58 (dt, J = 7.0, 1.2 Hz, 1H), 7.82 (dt, J = 8.9, 1.2 Hz, 1H), 7.60 (t, J = 1.8 Hz, 1H), 7.56 (ddd, J = 8.9, 6.9, 1.3 Hz, 1H), 7.43 (dt, J = 7.8, 1.3 Hz, 1H), 7.36 (ddd, J = 8.0, 2.0, 1.1 Hz, 1H), 7.34 – 7.29 (m, 2H), 7.22 – 7.10 (m, 4H), 6.97 (t, J = 7.8 Hz, 1H). ^{13}C NMR (151 MHz, $CDCl_3$) δ 185.4, 155.6, 147.6, 140.4, 134.4, 133.7, 132.6, 130.0, 129.6, 129.3, 128.6, 128.3, 127.9, 127.8, 121.8, 119.8, 117.5, 114.9. HRMS (ESI) m/z: $[M+H]^+$ Calcd for $C_{20}H_{13}BrN_2O$ 377.0284; found: 377.0274.

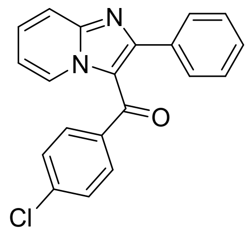

(4-chlorophenyl)(2-phenylimidazo[1,2-a]pyridin-3-yl)methanone **3m** prepared following general procedure C using compound **1a** (100 mg, 0.515 mmol) and 4-chlorobenzaldehyde **2f** (109 mg, 0.772 mmol). The product was purified by column chromatography (silica gel, Hexane/ethylacetate 3:1) to yield **3m** (94 mg, 55 %) as a white solid. ^{1}H NMR (600 MHz, $CDCl_3$) δ 9.53 (dt, *J* = 6.9, 1.2 Hz, 1H), 7.84 (dt, *J* = 8.9, 1.2 Hz, 1H), 7.54 (ddd, *J* = 9.0, 6.9, 1.3 Hz, 1H), 7.45 – 7.40 (m, 2H), 7.33 – 7.29 (m, 2H), 7.23 – 7.17 (m, 1H), 7.16 – 7.08 (m, 3H), 7.08 – 7.03 (m, 2H). ^{13}C NMR (151 MHz, $CDCl_3$) δ 185.8, 154.9, 147.4, 137.9, 137.0, 133.6, 130.8, 130.2, 129.5, 128.5, 128.5, 128.2, 128.0, 127.9, 117.4, 114.8. [12]

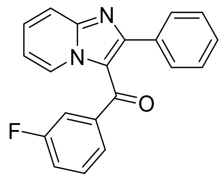

(3-fluorophenyl)(2-phenylimidazo[1,2-a]pyridin-3-yl)methanone **3n** prepared following general procedure C using compound **1a** (100 mg, 0.515 mmol) and 3-fluorobenzaldehyde **2g** (96 mg, 0.772 mmol). The product was purified by column chromatography (silica gel, Hexane/ethylacetate 3:1) to yield **3n** (80 mg, 49 %) as a brown solid. ^{1}H NMR (600 MHz, $CDCl_3$) δ 9.56 (dt, *J* = 7.0, 1.2 Hz, 1H), 7.84 (dt, *J* = 8.9, 1.2 Hz, 1H), 7.56 (ddd, *J* = 8.9, 6.9, 1.3 Hz, 1H), 7.35 – 7.29 (m, 2H), 7.25 – 7.20 (m, 2H), 7.20 – 7.16 (m, 1H), 7.13 (td, *J* = 6.9, 1.2 Hz, 3H), 7.03 (td, *J* = 8.0, 5.5 Hz, 1H), 6.95 (tdd, *J* = 8.3, 2.6, 1.0 Hz, 1H). ^{13}C NMR (151 MHz, $CDCl_3$) δ 185.7 (d, *J* = 2.2 Hz), 162.1 (d, *J* = 247.6 Hz), 155.4, 147.5, 140.7 (d, *J* = 6.5 Hz), 133.7, 130.1, 129.6, 129.3 (d, *J* = 8.0 Hz), 128.5, 128.3, 127.8, 125.3 (d, *J* = 2.9 Hz), 119.8, 118.5 (d, *J* = 21.2 Hz), 117.5, 116.2 (d, *J* = 22.8 Hz), 114.9. ^{19}F NMR (565 MHz, $CDCl_3$) δ113.2. [13]

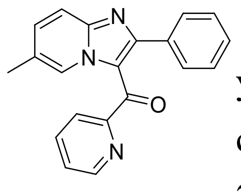

(6-methyl-2-phenylimidazo[1,2-a]pyridin-3-yl)(pyridin-2-yl)methanone **3o** prepared following general procedure C using compound **1g** (108 mg, 0.515 mmol) and pyridine-2-carbaldehyde **2a** (83 mg, 0.772 mmol). The product was purified by column chromatography (silica gel, Hexane/ethylacetate 2:1) to yield **3o** (113 mg, 70 %) as a white solid. ^{1}H NMR (600 MHz, $CDCl_3$) δ 9.47 (dt, *J* = 2.0, 1.1 Hz, 1H), 8.08

(ddd, J = 4.8, 1.7, 1.0 Hz, 1H), 7.70 (dd, J = 9.0, 1.0 Hz, 1H), 7.66 (dt, J = 7.8, 1.1 Hz, 1H), 7.57 (td, J = 7.7, 1.7 Hz, 1H), 7.39 (dd, J = 9.0, 1.8 Hz, 1H), 7.30 – 7.25 (m, 2H), 7.12 – 7.02 (m, 4H), 2.44 (d, J = 1.3 Hz, 3H). ^{13C} NMR (151 MHz, $CDCl_3$) δ 185.3, 156.6, 155.7, 148.3, 146.6, 136.1, 134.8, 132.4, 129.6, 127.7, 127.6, 126.3, 125.2, 124.9, 123.8, 119.7, 116.6, 18.4. HRMS (ESI) m/z: $[M+H]^+$ Calcd for $C_{20}H_{15}N_3O$ 314.1287; found: 314.1290.

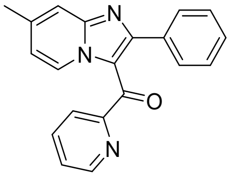

(7-methyl-2-phenylimidazo[1,2-a]pyridin-3-yl)(pyridin-2-yl)methanone **3p** prepared following general procedure C using compound **1h** (108 mg, 0.515 mmol) and pyridine-2-carbaldehyde **2a** (83 mg, 0.772 mmol). The product was purified by column chromatography (silica gel, Hexane/ethylacetate 2:1) to yield **3p** (97 mg, 60 %) as a white solid. ^{1}H NMR (600 MHz, $CDCl_3$) δ 9.54 (dd, J = 7.0, 0.9 Hz, 1H), 8.08 (ddd, J = 4.8, 1.7, 0.9 Hz, 1H), 7.65 (dt, J = 7.8, 1.1 Hz, 1H), 7.61 – 7.53 (m, 2H), 7.31 – 7.23 (m, 2H), 7.13 – 7.00 (m, 4H), 6.96 (dd, J = 7.1, 1.8 Hz, 1H), 2.51 (d, J = 1.2 Hz, 3H). ^{13}C NMR (151 MHz, $CDCl_3$) δ 185.0, 157.1, 155.8, 148.3, 148.1, 141.3, 136.1, 134.9, 129.6, 127.8, 127.7, 127.6, 125.2, 123.8, 119.7, 117.3, 116.1, 21.6. HRMS (ESI) m/z: $[M+H]^+$ Calcd for $C_{20}H_{15}N_3O$ 314.1287; found: 314.1283.

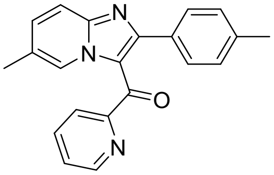

(6-methyl-2-(p-tolyl)imidazo[1,2-a]pyridin-3-yl)(pyridin-2-yl)methanone **3q** prepared following general procedure C using compound **1i** (115 mg, 0.515 mmol) and pyridine-2-carbaldehyde **2a** (83 mg, 0.772 mmol). The product was purified by column chromatography (silica gel, Hexane/ethylacetate 2:1) to yield **3q** (118 mg, 70 %) as a white solid. ^{1}H NMR (600 MHz, $CDCl_3$) δ 9.46 (dt, J = 2.0, 1.1 Hz, 1H), 8.12 (ddd, J = 4.7, 1.7, 0.9 Hz, 1H), 7.69 (dd, J = 9.0, 0.9 Hz, 1H), 7.64 (dt, J = 7.8, 1.1 Hz, 1H), 7.57 (td, J = 7.7, 1.7 Hz, 1H), 7.38 (dd, J = 9.1, 1.8 Hz, 1H), 7.19 – 7.14 (m, 2H), 7.10 (ddd, J = 7.6, 4.8, 1.3 Hz, 1H), 6.88 – 6.83 (m, 2H), 2.45 (d, J = 1.3 Hz, 3H), 2.22 (s, 3H). ^{13}C NMR (151 MHz, $CDCl_3$) δ 185.4, 156.7, 155.9, 148.4, 146.6, 137.6, 136.1, 132.4, 131.8, 129.5, 128.3, 126.3, 125.0, 124.8,

123.8, 119.6, 116.5, 21.1, 18.5. HRMS (ESI) m/z: $[M+H]^+$ Calcd for $C_{21}H_{17}N_3O$ 328.1444; found: 328.14436.

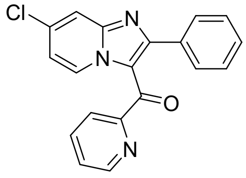

(7-chloro-2-phenylimidazo[1,2-a]pyridin-3-yl)(pyridin-2-yl)methanone **3r** prepared following general procedure C using compound **1j** (118 mg, 0.515 mmol) and pyridine-2-carbaldehyde **2a** (83 mg, 0.772 mmol). The product was purified by column chromatography (silica gel, Hexane/ethylacetate 2:1) to yield **3r** (122 mg, 71 %) as a white solid. ^{1}H NMR (600 MHz, $CDCl_3$) δ 9.55 (dd, *J* = 7.4, 0.8 Hz, 1H), 8.06 (ddd, *J* = 4.8, 1.7, 0.9 Hz, 1H), 7.79 (dd, *J* = 2.2, 0.8 Hz, 1H), 7.70 (dt, *J* = 7.8, 1.1 Hz, 1H), 7.61 (td, *J* = 7.7, 1.7 Hz, 1H), 7.31 – 7.23 (m, 2H), 7.17 – 7.01 (m, 5H). ^{13}C NMR (151 MHz, $CDCl_3$) δ 185.4, 157.3, 155.2, 148.4, 147.5, 136.2, 136.0, 134.3, 129.6, 128.7, 128.1, 127.7, 125.5, 123.8, 119.9, 116.4, 116.2. HRMS (ESI) m/z: $[M+H]^+$ Calcd for $C_{19}H_{12}ClN_3O$ 334.0741; found: 334.0726.

General procedure C for preparation of 3,3'-(phenylmethylene)bis(2-phenylimidazo[1,2-a]pyridine) 4a and derivatives

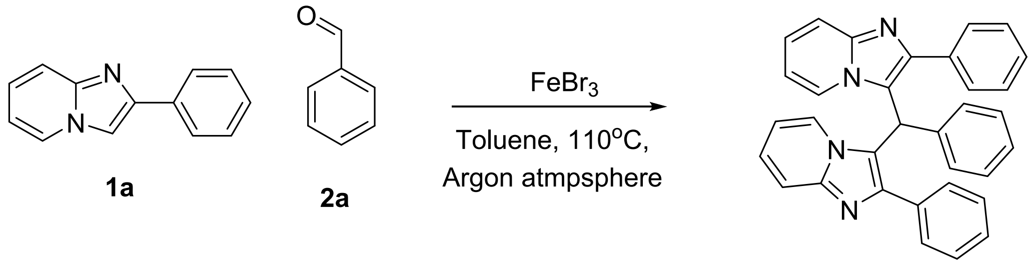

4a

Weigh into a reaction tube, compound **1a** (100 mg, 0.515 mmol), benzaldehyde **2a** (54.6 mg, 0.515 mmol), $FeBr_3$ (30.4 mg, 0.103 mmol) and toluene (0.6 mL). Backfilled the reaction tube with argon gas. Stir the reaction mixture at 110°C for 16 hours. The reaction mixture was extracted with water and ethylacetate. Dry the organic layer over anhydrous sodium sulfate. Evaporate the solution to dryness. Purify the crude residue by column chromatography (silica gel, Hexane/ethylacetate 2:1), obtain 3,3'-(phenylmethylene)bis(2-phenylimidazo[1,2-a]pyridine) **4a** as a brown solid (56 mg, 45%). ^{1}H NMR (600 MHz, $CDCl_3$) δ 7.60

(dt, J = 9.0, 1.1 Hz, 2H), 7.35 – 7.28 (m, 4H), 7.23 (dt, J = 6.9, 1.2 Hz, 2H), 7.21 – 7.15 (m, 3H), 7.15 – 7.05 (m, 7H), 6.86 – 6.76 (m, 2H), 6.53 (s, 1H), 6.41 (td, J = 6.8, 1.2 Hz, 2H). ^{13}C NMR (151 MHz, $CDCl_3$) δ 145.3, 144.7, 135.7, 133.8, 129.2, 128.7, 128.0, 127.9, 127.7, 127.7, 124.5, 124.1, 118.0, 117.3, 112.2, 38.5. [14]

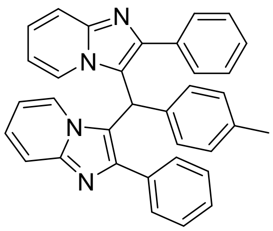

3,3'-(p-tolylmethylene)bis(2-phenylimidazo[1,2-a]pyridine) **4b** prepared following general procedure D using compound **1a** (100 mg, 0.515 mmol) and 4-methylbenzaldehyde **2b** (62 mg, 0.515 mmol). The product was purified by column chromatography (silica gel, Hexane/ethylacetate 2:1) to yield **4b** (81.91 mg, 50 %) as a brown solid. ^{1}H NMR (600 MHz, $CDCl_3$) δ 7.58 (dt, J = 9.0, 1.2 Hz, 2H), 7.36 – 7.28 (m, 4H), 7.24 (dt, J = 7.0, 1.2 Hz, 2H), 7.09 (dddd, J = 10.3, 7.9, 5.3, 1.1 Hz, 8H), 7.02 – 6.92 (m, 2H), 6.75 – 6.63 (m, 2H), 6.50 (s, 1H), 6.40 (td, J = 6.8, 1.2 Hz, 2H), 2.24 (s, 3H). ^{13}C NMR (151 MHz, $CDCl_3$) δ 145.3, 144.7, 137.3, 134.1, 132.6, 129.9, 128.7, 127.9, 127.8, 127.6, 124.3, 124.2, 118.2, 117.3, 112.0, 38.2, 20.9. HRMS (ESI) m/z: $[M+H]^+$ Calcd for $C_{34}H_{26}N_4$ 491.2230; found: 491.2215.

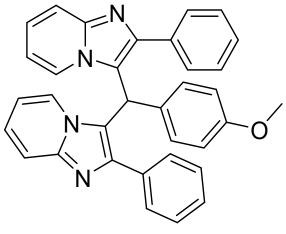

3,3'-((4-methoxyphenyl)methylene)bis(2-phenylimidazo[1,2-a]pyridine) **4c** prepared following general procedure D using compound **1a** (100 mg, 0.515 mmol) and 4-methoxylbenzaldehyde **2c** (70 mg, 0.515 mmol). The product was purified by column chromatography (silica gel, Hexane/ethylacetate 2:1) to yield **4c** (79 mg, 61 %) as a brown solid. ^{1}H NMR (600 MHz, $CDCl_3$) δ 7.58 (dt, J = 9.1, 1.2 Hz, 2H), 7.34 – 7.28 (m, 4H), 7.28 – 7.23 (m, 2H), 7.15 – 7.03 (m, 8H), 6.75 – 6.65 (m, 4H), 6.47 (s, 1H), 6.41 (td, J = 6.9, 1.3 Hz, 2H), 3.71 (s, 3H). ^{13}C NMR (151 MHz, $CDCl_3$) δ 158.8, 145.3, 144.7, 134.1, 129.0, 128.7, 127.9, 127.6, 127.5, 124.3, 124.2, 118.3, 117.3, 114.5, 112.0, 55.2, 37.8. HRMS (ESI) m/z: $[M+H]^+$ Calcd for $C_{34}H_{26}N_4O$ 507.2179; found: 507.2171.

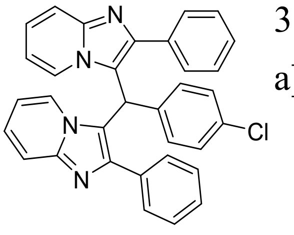

3,3'-((4-chlorophenyl)methylene)bis(2-phenylimidazo[1,2-a]pyridine) **4d** prepared following general procedure D using

compound **1a** (100 mg, 0.515 mmol) and 4-chlorobenzaldehyde **2d** (72 mg, 0.515 mmol). The product was purified by column chromatography (silica gel, Hexane/ethylacetate 2:1) to yield 7d (92 mg, 70 %) as a brown solid. ^{1}H NMR (600 MHz, $CDCl_3$) δ 7.59 (dt, *J* = 9.0, 1.2 Hz, 2H), 7.33 – 7.26 (m, 4H), 7.21 (dt, *J* = 7.0, 1.2 Hz, 2H), 7.17 – 7.06 (m, 10H), 6.78 – 6.70 (m, 2H), 6.48 (s, 1H), 6.44 (td, *J* = 6.9, 1.3 Hz, 2H). ^{13}C NMR (151 MHz, $CDCl_3$) δ 145.6, 144.8, 134.5, 133.9, 133.5, 129.3, 129.3, 128.7, 128.0, 127.7, 124.5, 123.9, 117.5, 117.4, 112.3, 38.1. HRMS (ESI) m/z: $[M+H]^+$ Calcd for $C_{33}H_{23}ClN_4$ 511.1684; found: 511.1676.

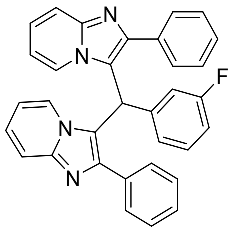

3,3'-((3-fluorophenyl)methylene)bis(2-phenylimidazo[1,2-a]pyridine) **4e** prepared following general procedure D using compound **1a** (100 mg, 0.515 mmol) and 3-fluorobenzaldehyde **2e** (64 mg, 0.515 mmol). The product was purified by column chromatography (silica gel, Hexane/ethylacetate 2:1) to yield **4e** (65 mg, 51 %) as a brown solid. ^{1}H NMR (600 MHz, $CDCl_3$) δ 7.60 (dt, *J* = 9.1, 1.1 Hz, 2H), 7.31 – 7.27 (m, 4H), 7.21 (dt, *J* = 6.9, 1.2 Hz, 2H), 7.18 – 7.05 (m, 9H), 6.90 – 6.83 (m, 1H), 6.59 (ddt, *J* = 7.8, 1.7, 0.9 Hz, 1H), 6.53 – 6.48 (m, 2H), 6.43 (td, *J* = 6.9, 1.3 Hz, 2H). ^{13}C NMR (151 MHz, $CDCl_3$) δ 163.3 (d, *J* = 248.3 Hz), 145.6, 144.8, 138.7 (d, *J* = 6.4 Hz), 133.8, 130.8 (d, *J* = 8.2 Hz), 128.7, 128.0, 127.8, 124.5, 123.8, 123.6 (d, *J* = 2.8 Hz), 117.5, 117.4, 115.0 (d, *J* = 22.5 Hz), 114.8 (d, *J* = 21.1 Hz), 112.3, 38.3. HRMS (ESI) m/z: $[M+H]^+$ Calcd for $C_{33}H_{23}FN_4$ 495.1979; found: 495.1963.

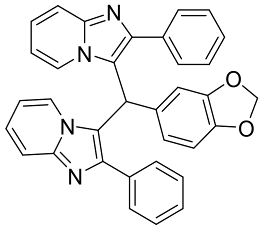

3,3'-(benzo[d][1,3]dioxol-5-ylmethylene)bis(2-phenylimidazo[1,2-a]pyridine) 4f prepared following general procedure D using compound **1a** (100 mg, 0.515 mmol) and benzo[d][1,3]dioxole-5-carbaldehyde **2k** (77 mg, 0.515 mmol). The product was purified by column chromatography (silica gel, Hexane/ethylacetate 2:1) to yield **4f** (84 mg, 63 %) as a brown solid. ^{1}H NMR (600 MHz, $CDCl_3$) δ 7.59 (d, *J* = 9.0 Hz, 2H), 7.28 (d, *J* = 11.1 Hz, 6H), 7.15 – 7.07 (m, 8H), 6.60 (dd, *J* = 8.1, 1.2 Hz, 1H), 6.43 (d, *J* = 20.0 Hz, 3H), 6.30 (d, *J* = 1.8

Hz, 1H), 6.25 (ddd, J = 8.1, 1.9, 0.9 Hz, 1H), 5.86 (d, J = 1.7 Hz, 2H). ^{13}C NMR (151 MHz, $CDCl_3$) δ 148.5, 147.0, 145.4, 144.7, 134.0, 129.5, 128.7, 127.9, 127.6, 124.3, 124.1, 121.0, 118.0, 117.4, 112.1, 108.5, 108.4, 101.3, 38.3. HRMS (ESI) m/z: $[M+H]^+$ Calcd for $C_{34}H_{24}N_4O_2$ 521.1972; found: 521.1952.

Procedure for the preparation of (*E*) 3-(hex-1-en-1-yl)-2-phenylimidazo[1,2-*a*]pyridine 5a

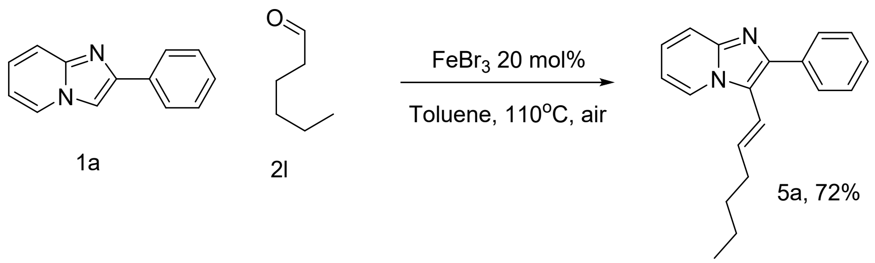

1a 2l 5a, 72%

In a reaction tube, 2-phenylimidazo[1,2-a]pyridine **1a** (100 mg, 0.515 mmol), hexanal **2l** (77 mg, 0.772 mmol), and iron(III) bromide ($FeBr_3$) (30.4 mg, 0.103 mmol) were dissolved in toluene (0.6 mL). The reaction tube was tightly capped and the reaction mixture was stirred at 110°C for 16 hours. The reaction mixture was then extracted with water and ethyl acetate. The organic layer was dried over anhydrous sodium sulfate, and the solvent was removed under reduced pressure. The crude residue was purified by column chromatography on silica gel using a hexane/ethyl acetate (5:1) solvent system to afford 3-(hex-1-en-1-yl)-2-phenylimidazo[1,2-*a*]pyridine **5a** as a white solid (106.3 mg, 72%).^{1}H NMR (600 MHz, $CDCl_3$) δ 8.2 (dt, J = 7.0, 1.2 Hz, 1H), 7.9 – 7.8 (m, 2H), 7.7 (dt, J = 9.0, 1.1 Hz, 1H), 7.4 (dd, J = 8.4, 6.9 Hz, 2H), 7.3 (ddt, J = 8.6, 7.0, 1.3 Hz, 1H), 7.1 (ddd, J = 9.0, 6.7, 1.2 Hz, 1H), 6.8 (td, J = 6.8, 1.3 Hz, 1H), 6.5 (dt, J = 16.3, 1.5 Hz, 1H), 6.2 (dt, J = 16.3, 7.0 Hz, 1H), 2.4 – 2.3 (m, 2H), 1.5 – 1.5 (m, 2H), 1.5 – 1.4 (m, 2H), 1.0 (t, J = 7.3 Hz, 3H). ^{13}C NMR (151 MHz, $CDCl_3$) δ 144.9, 143.0, 135.8, 134.7, 128.7, 128.4, 127.6, 124.3, 123.8, 119.0, 117.6, 117.0, 112.4, 33.4,

31.4, 22.3, 13.9. HRMS (ESI) m/z: $[M+H]^+$ Calcd for $C_{19}H_{20}N_2$ 277.1699; found: 277.1688.

2. Copies of NMR spectra

2-phenylimidazo[1,2-a]pyridine 1a

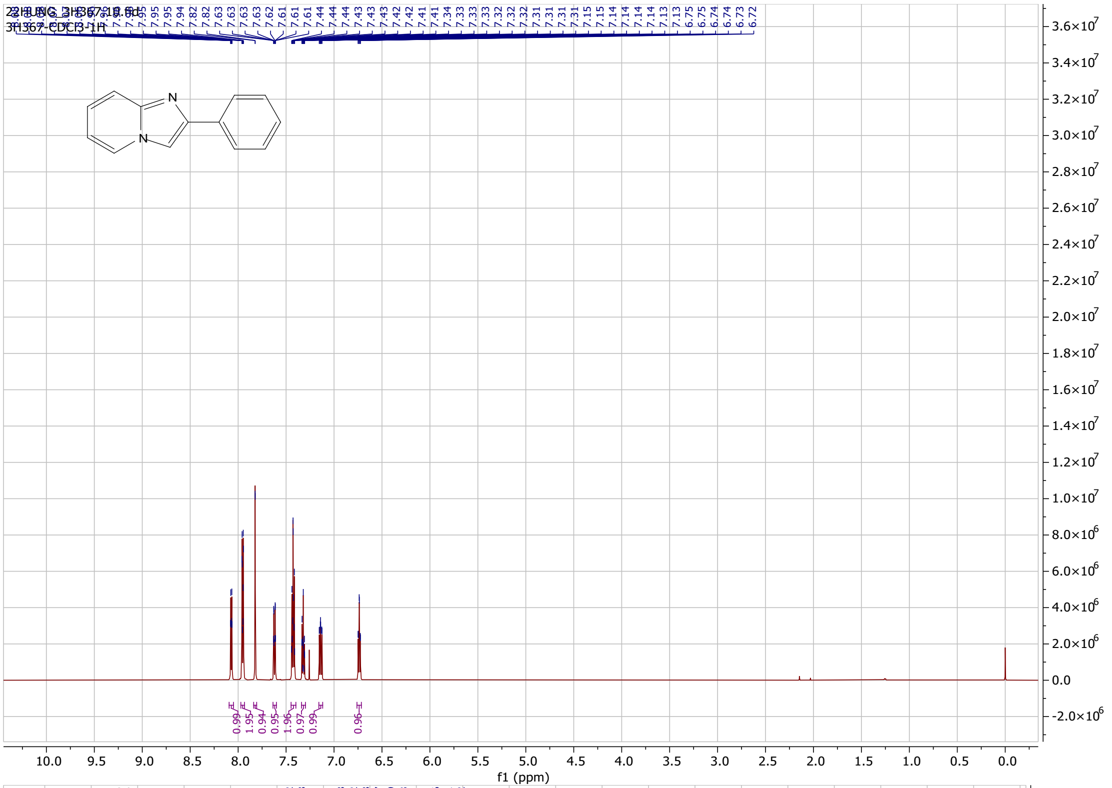

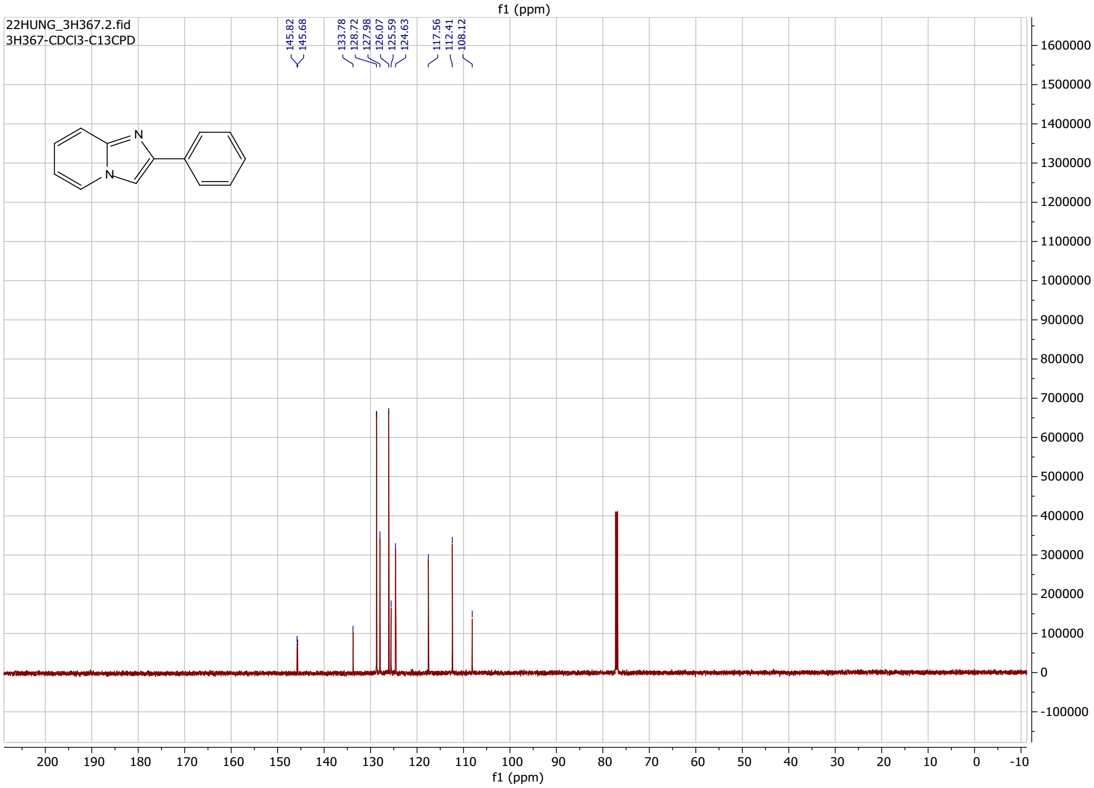

2-(p-tolyl)imidazo[1,2-a]pyridine 1b

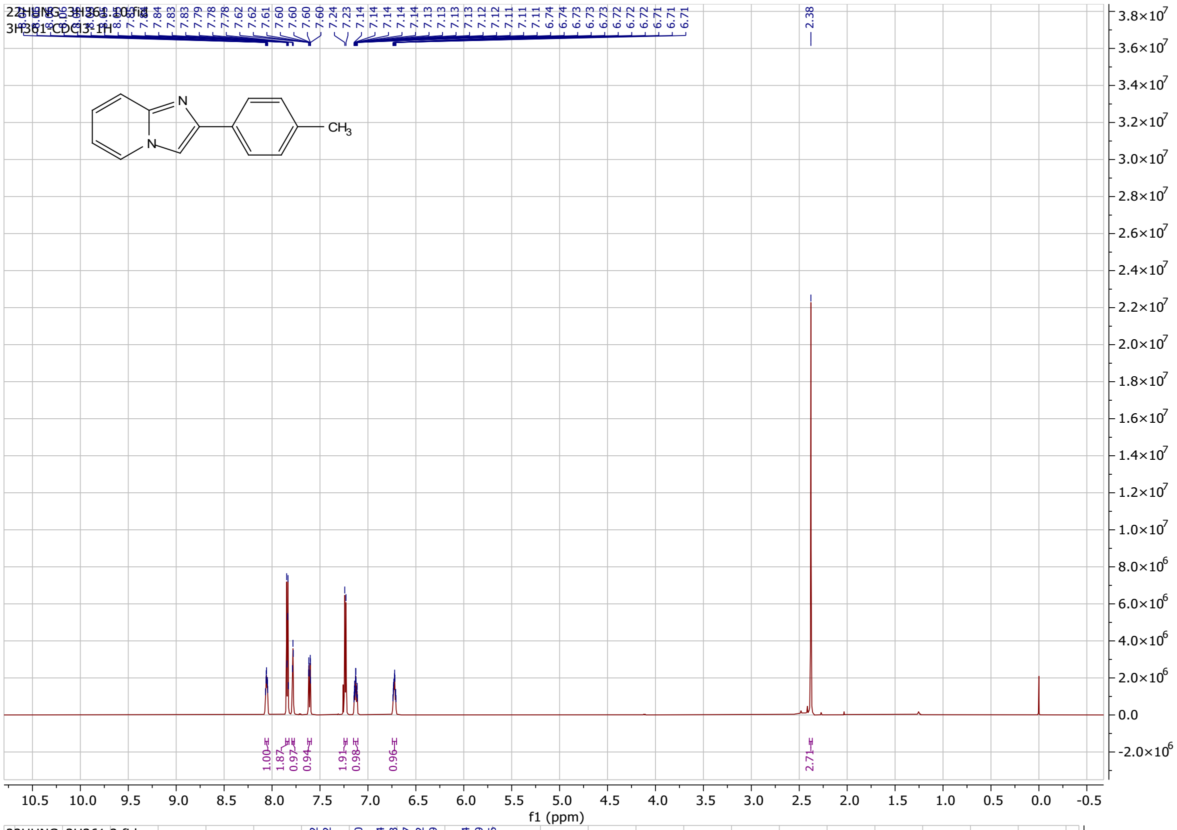

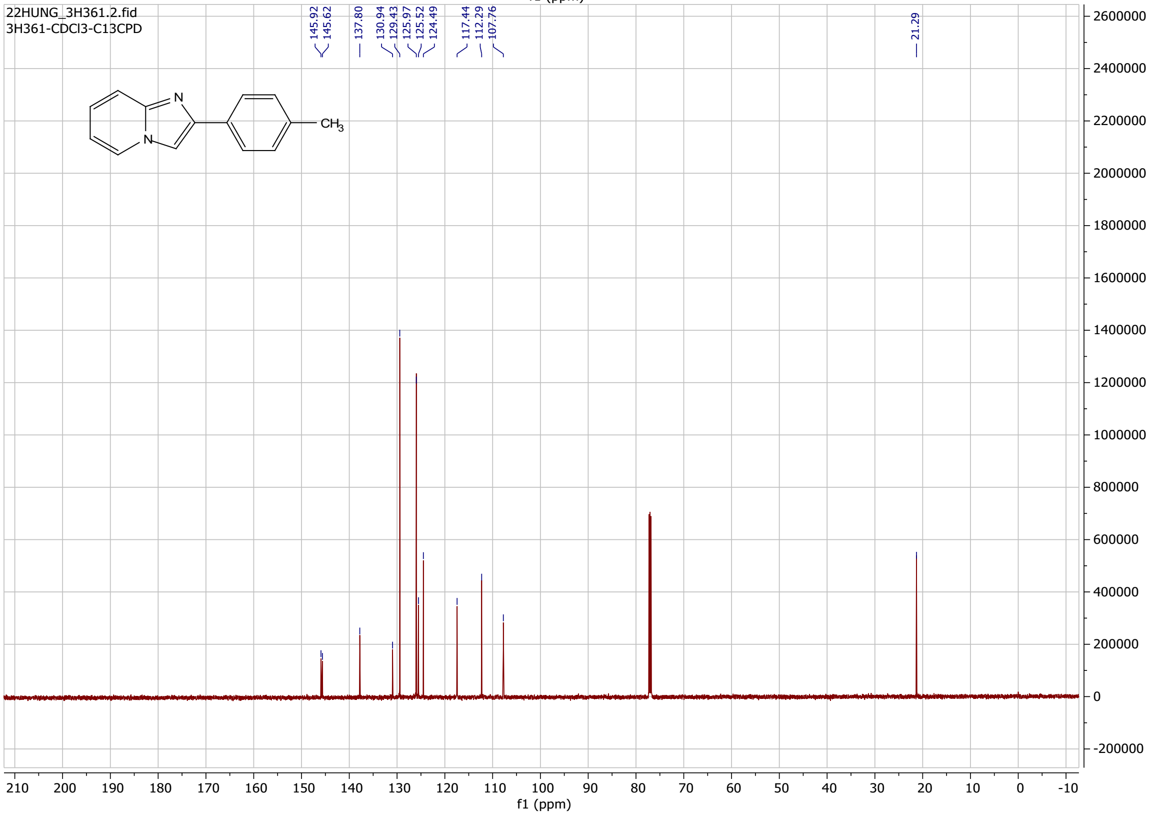

2-(4-ethylphenyl)imidazo[1,2-a]pyridine 1c

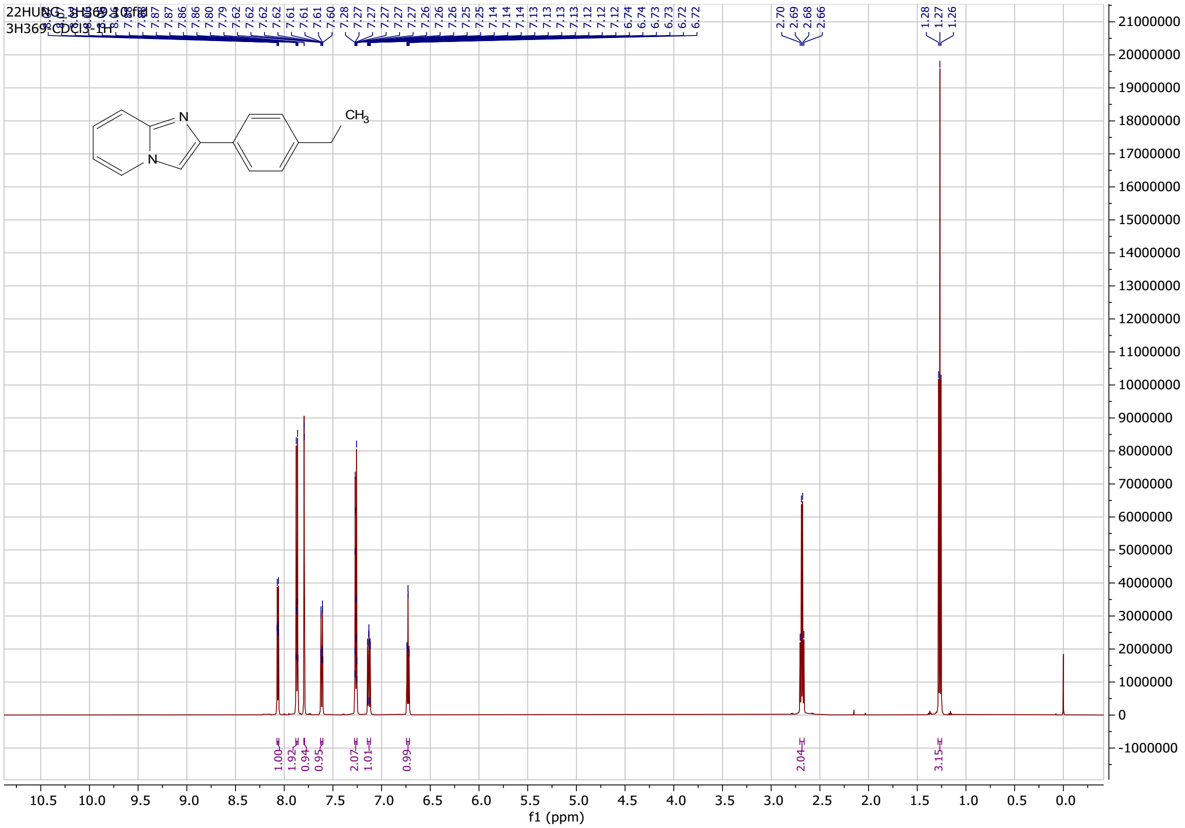

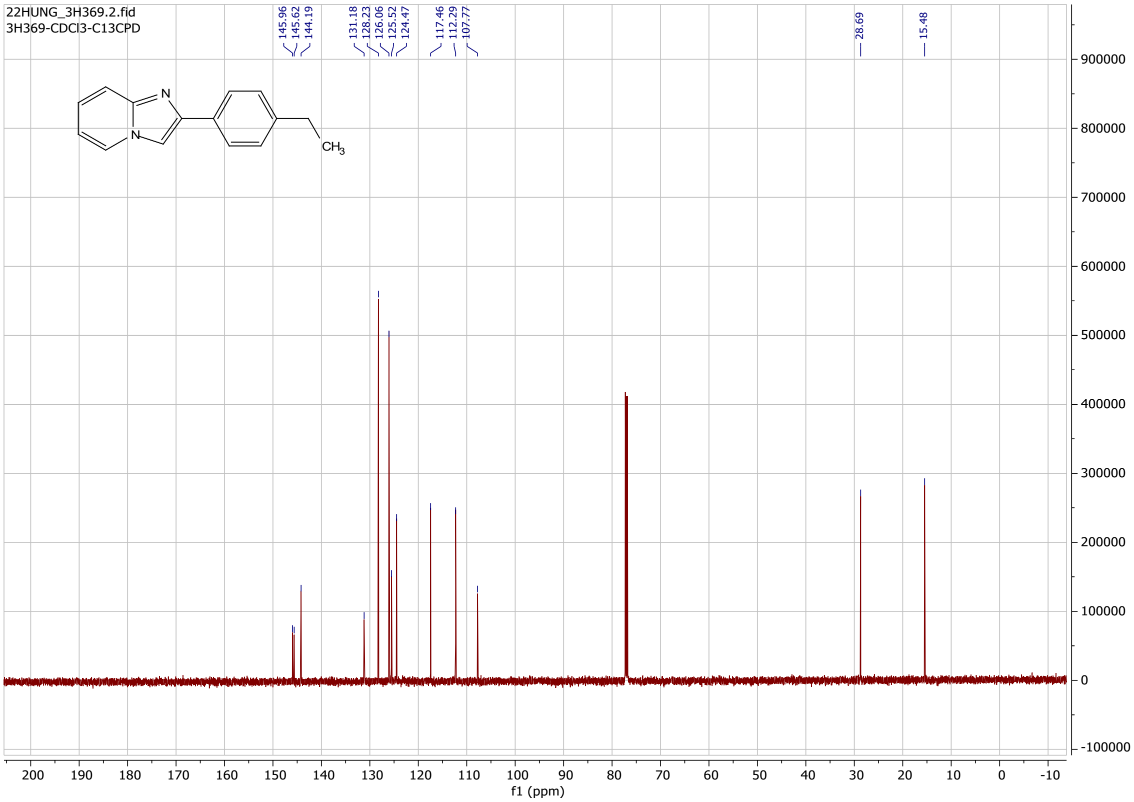

2-(4-methoxyphenyl)imidazo[1,2-a]pyridine 1d

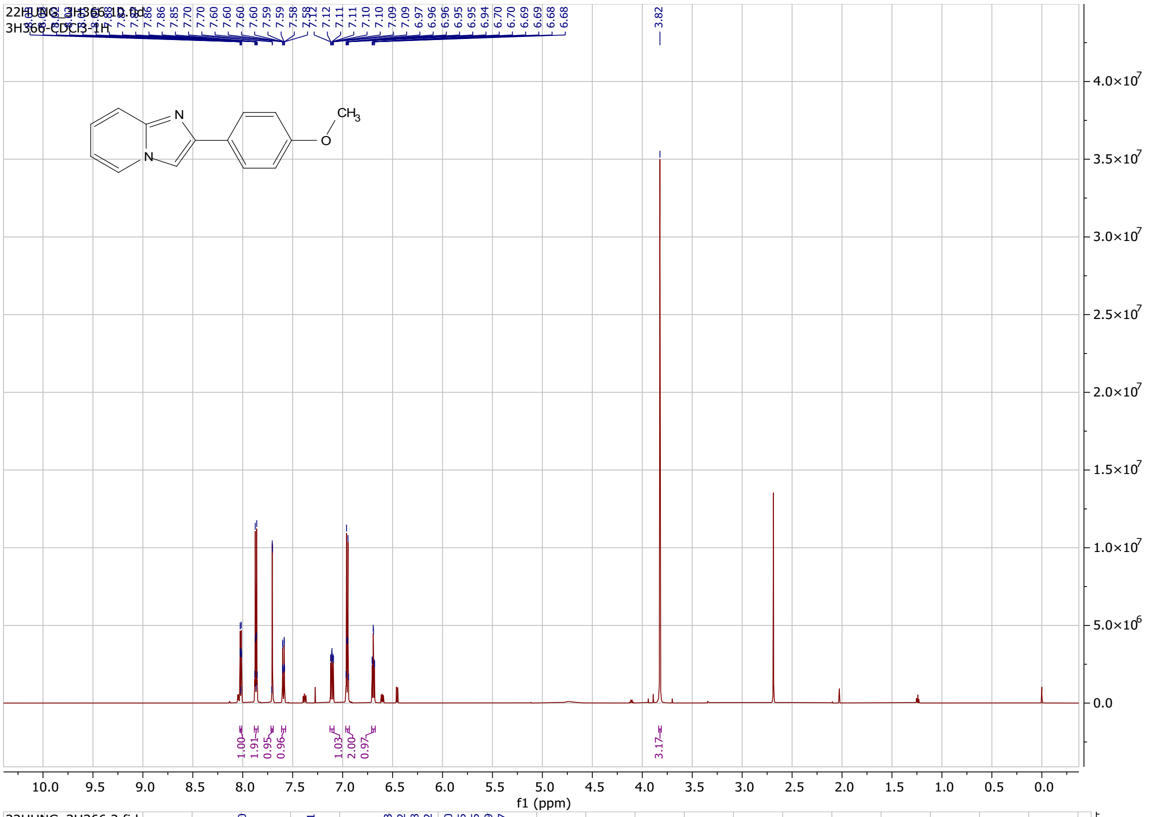

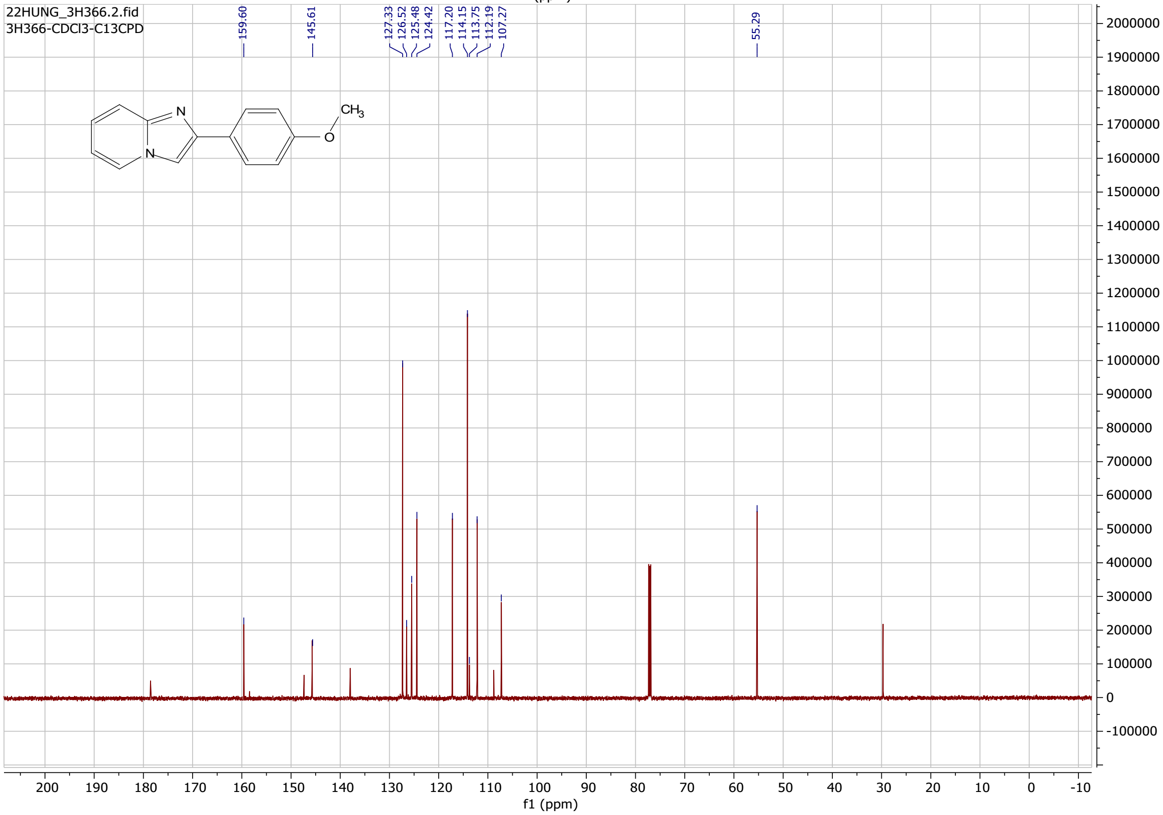

2-(4-fluorophenyl)imidazo[1,2-a]pyridine 1e

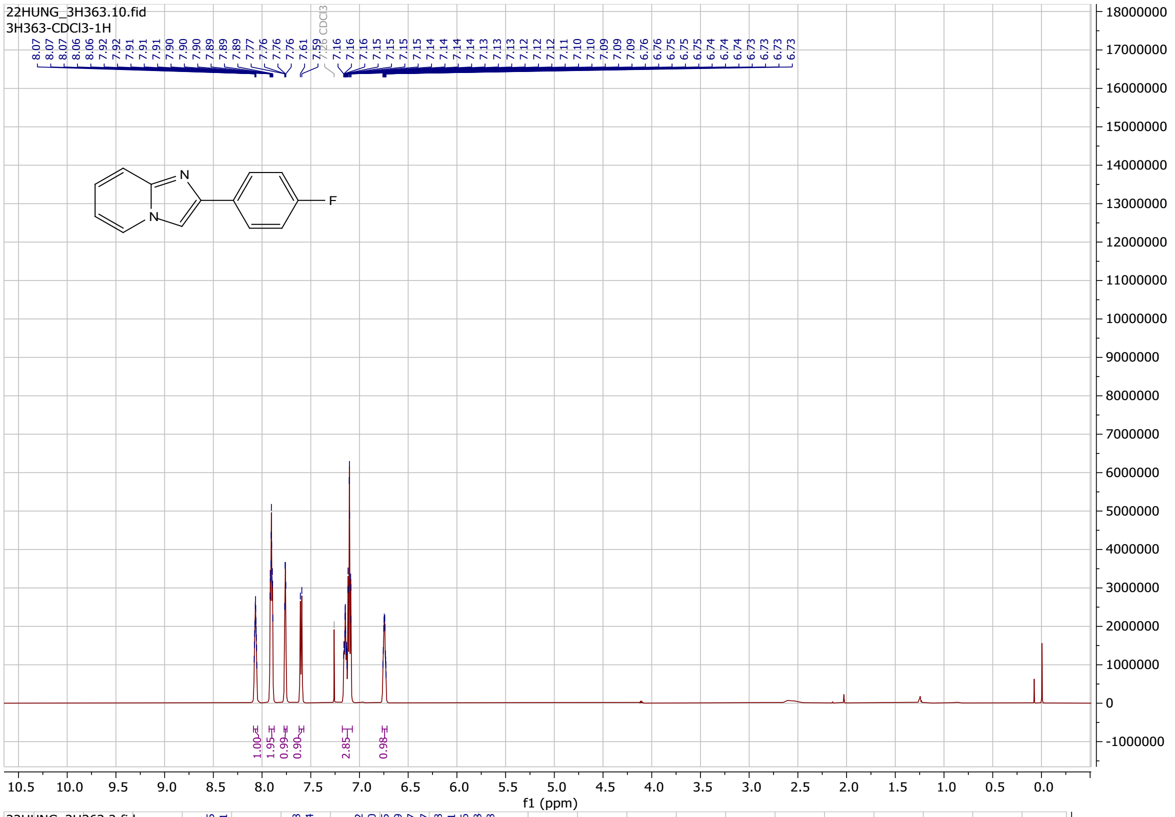

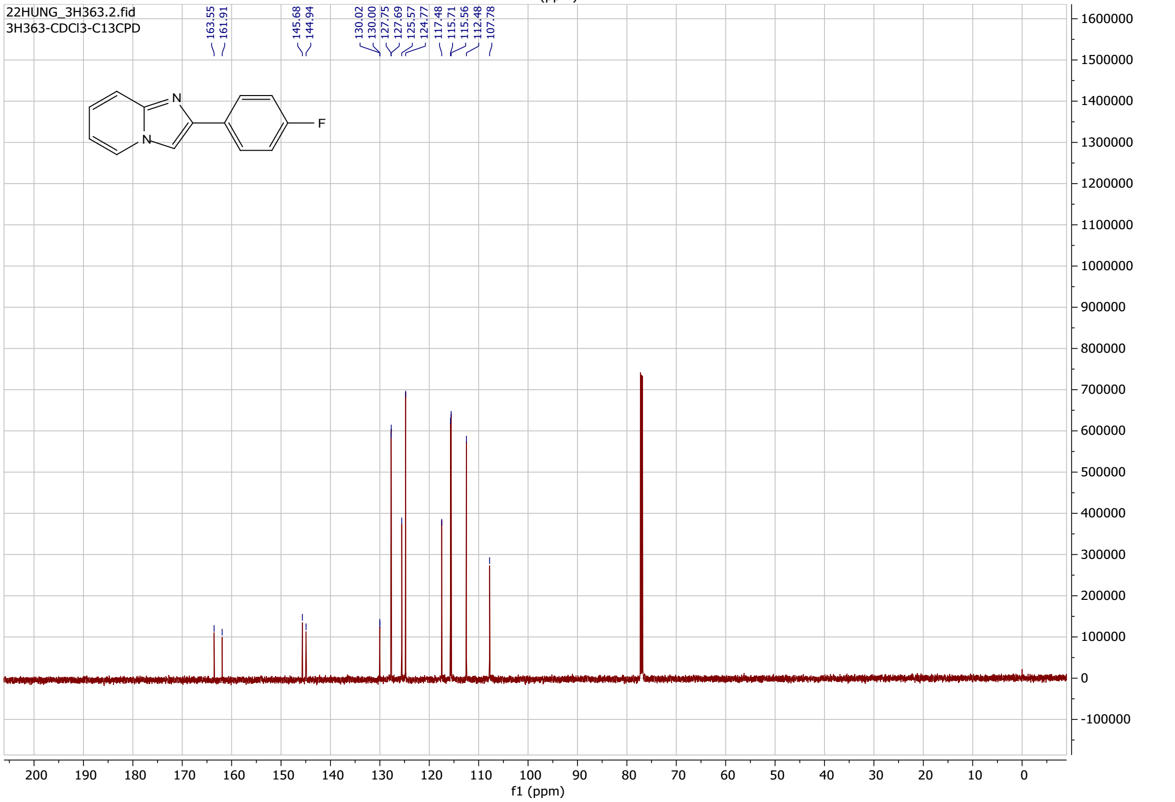

2-(4-chlorophenyl)imidazo[1,2-a]pyridine 1f

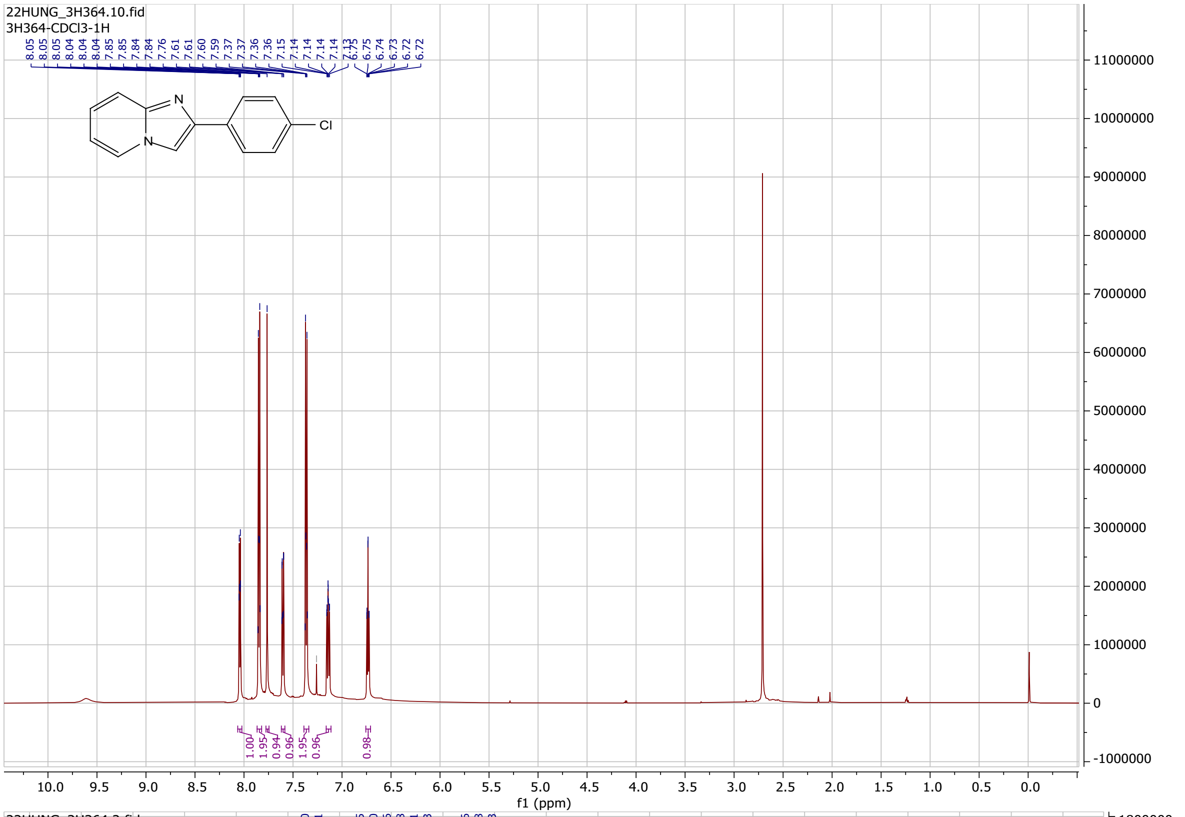

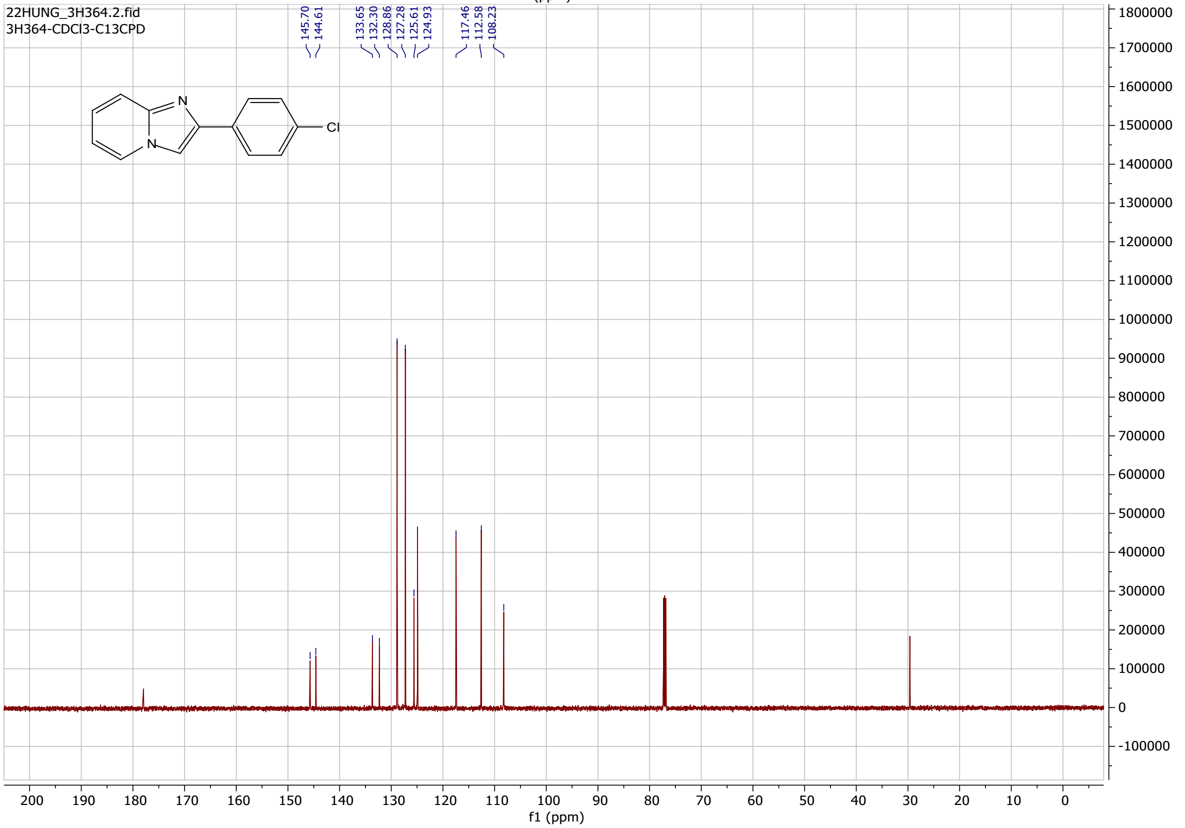

6-methyl-2-phenylimidazo[1,2-a]pyridine 1g

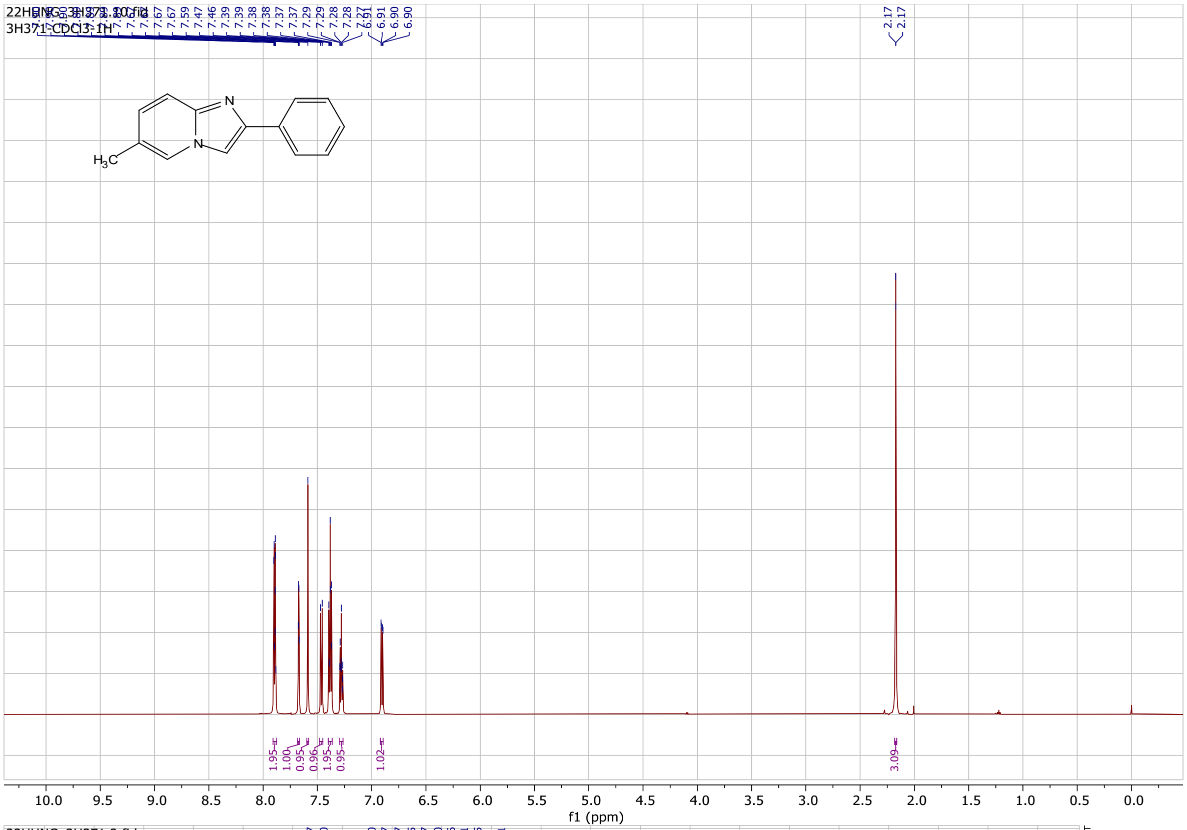

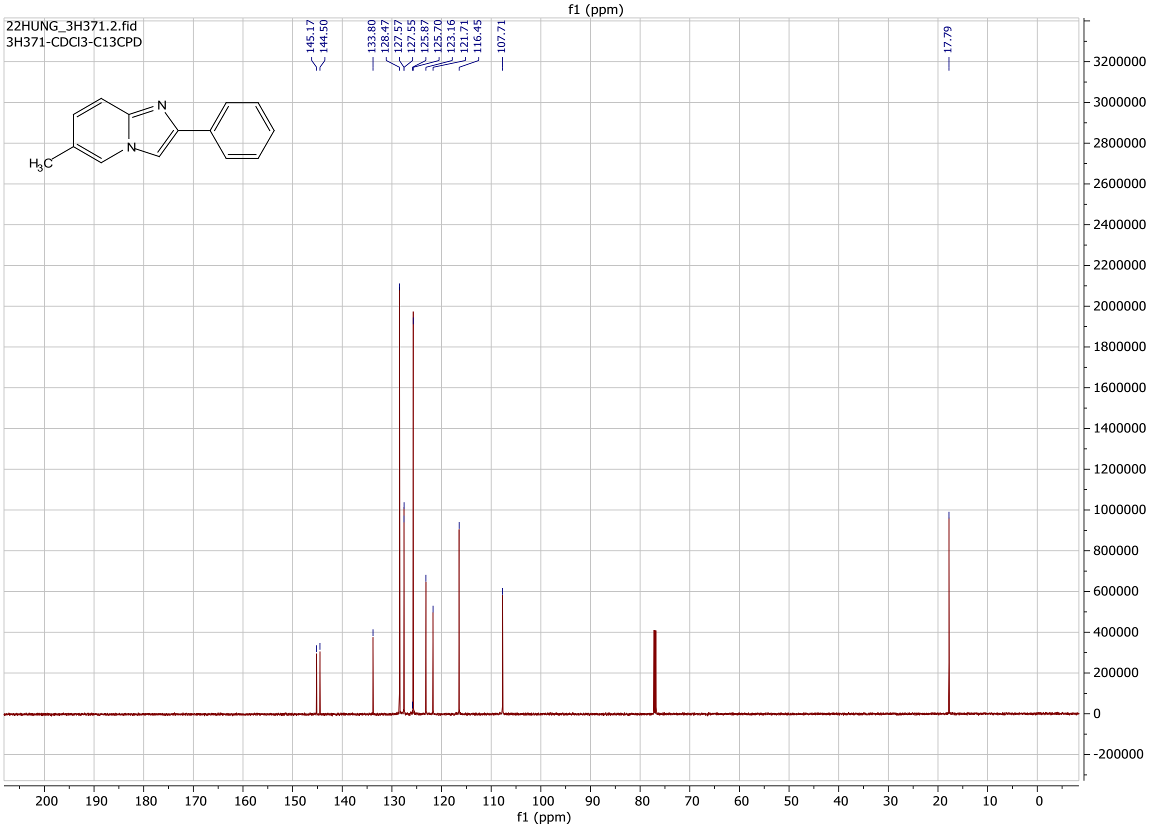

7-methyl-2-phenylimidazo[1,2-a]pyridine 1h

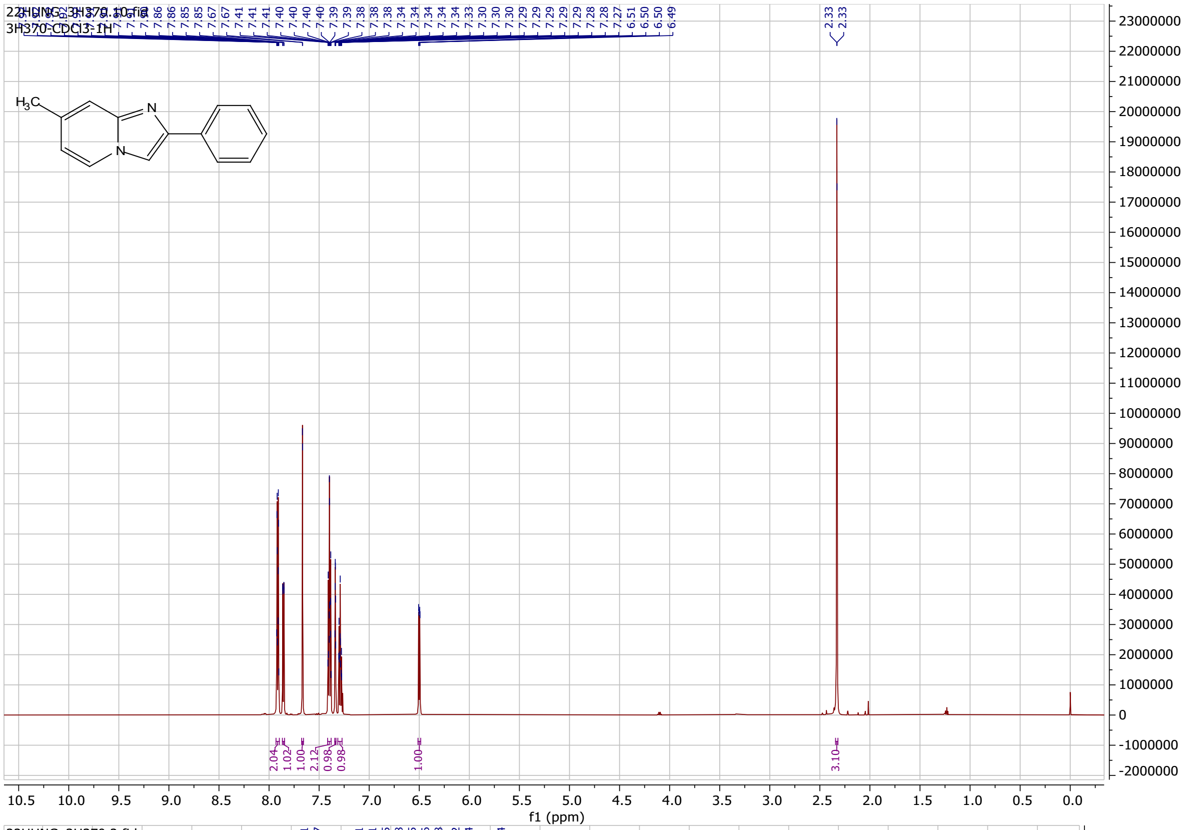

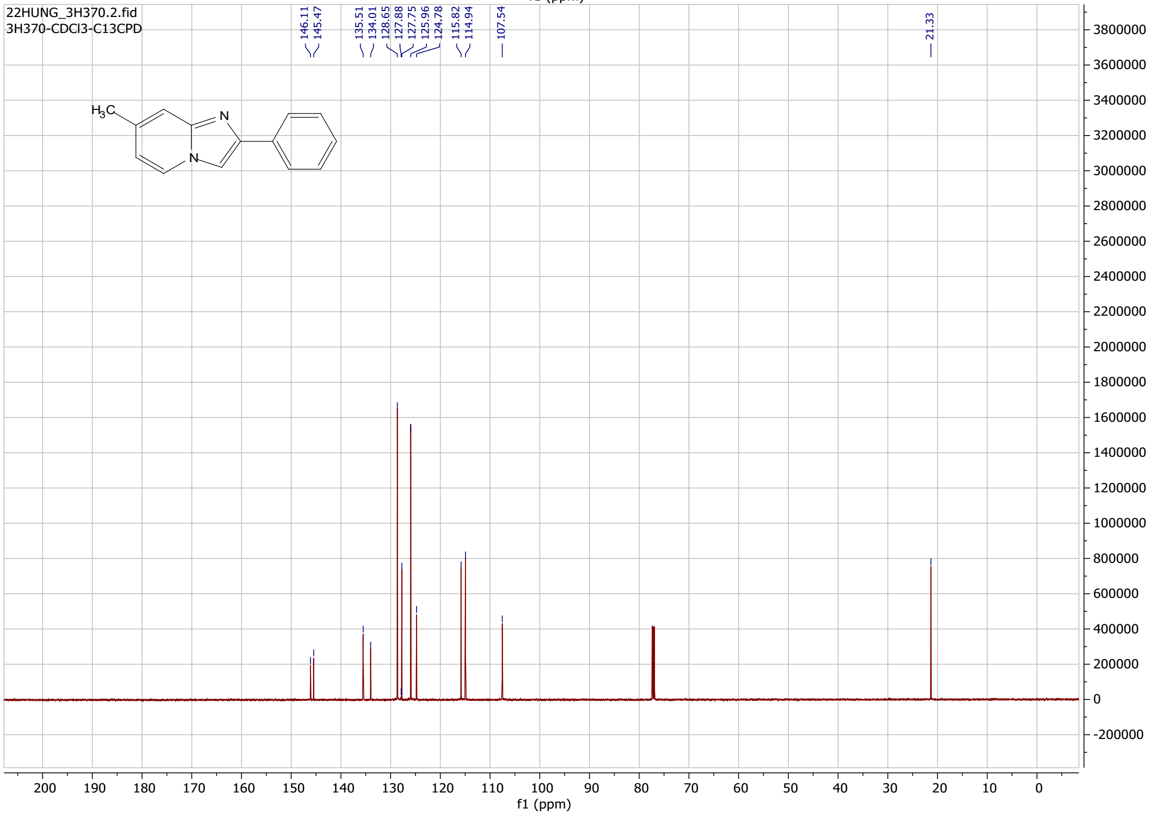

6-methyl-2-(p-tolyl)imidazo[1,2-a]pyridine 1i

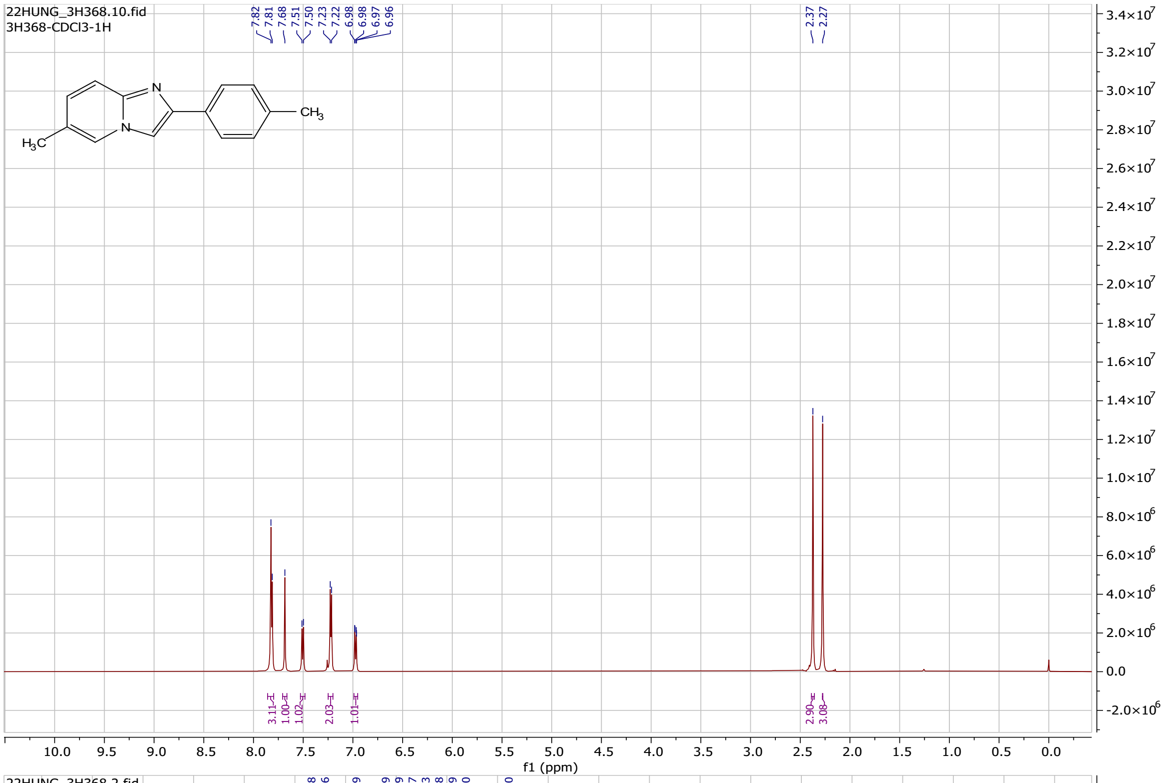

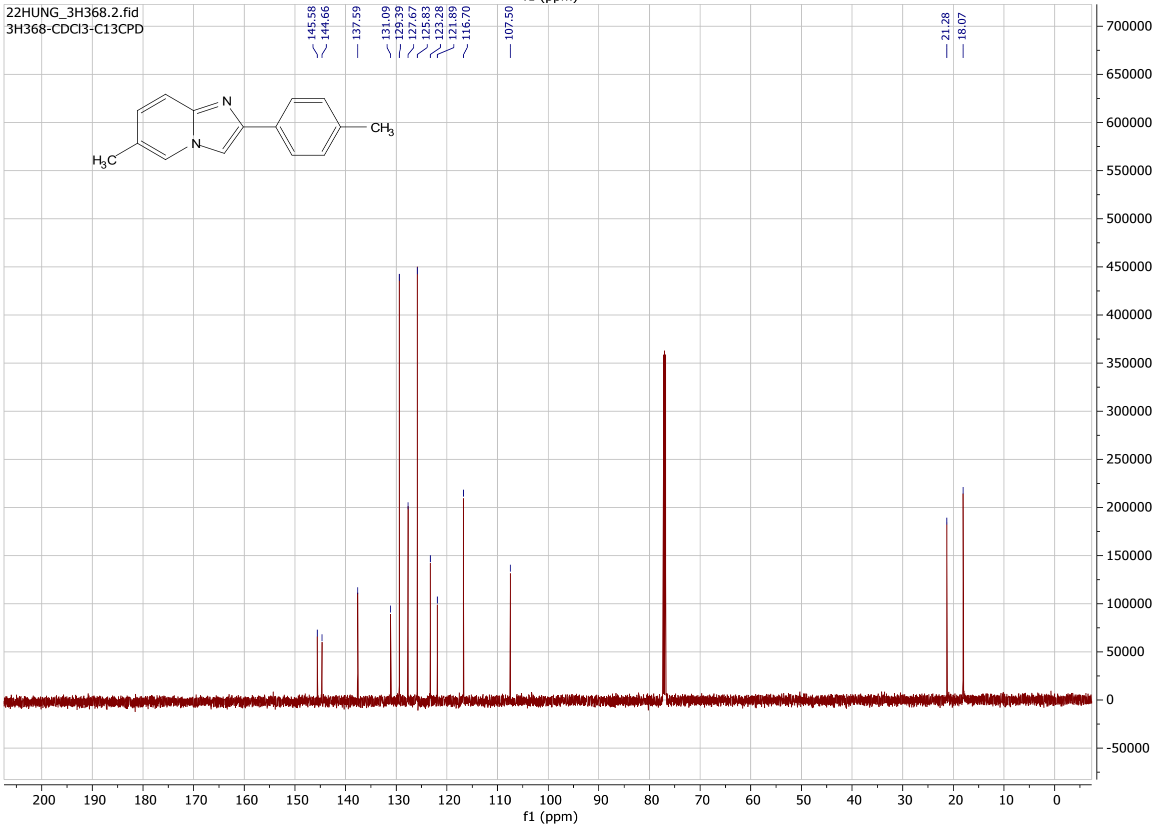

7-chloro-2-phenylimidazo[1,2-a]pyridine 1j

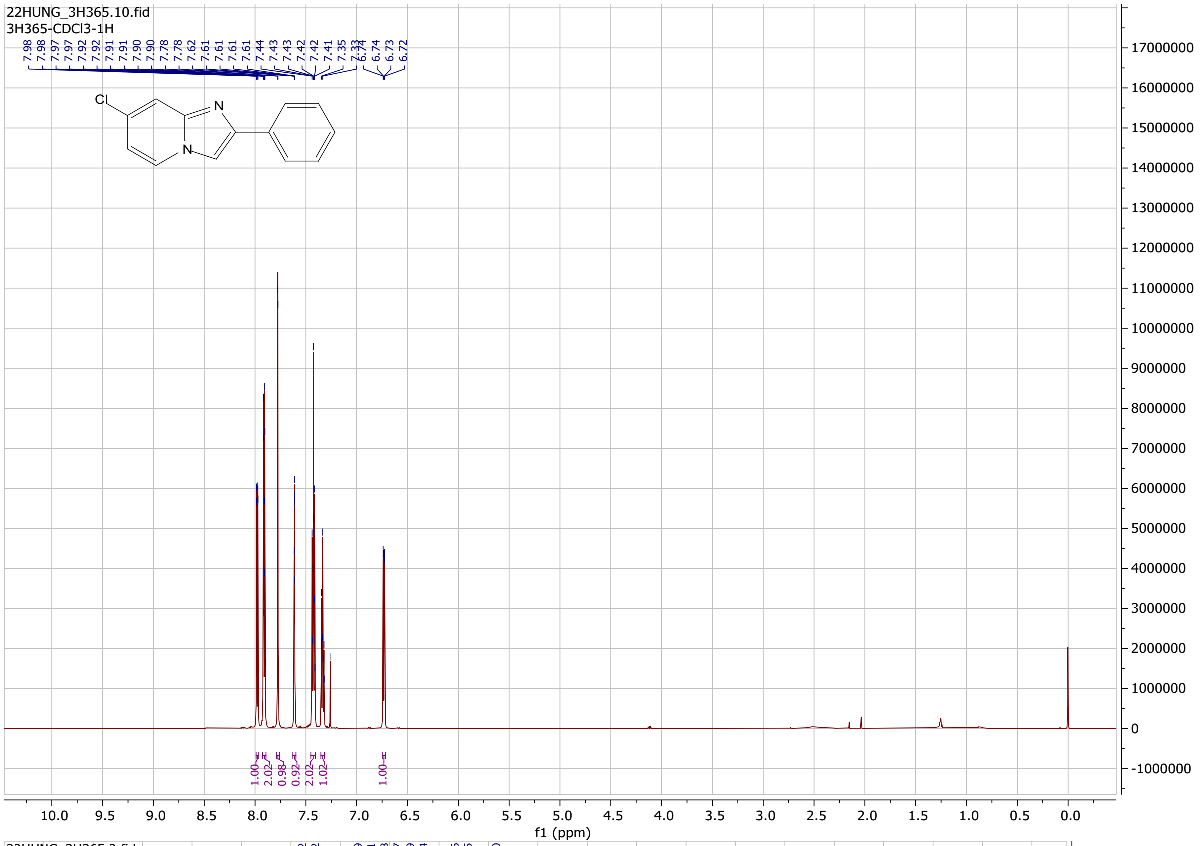

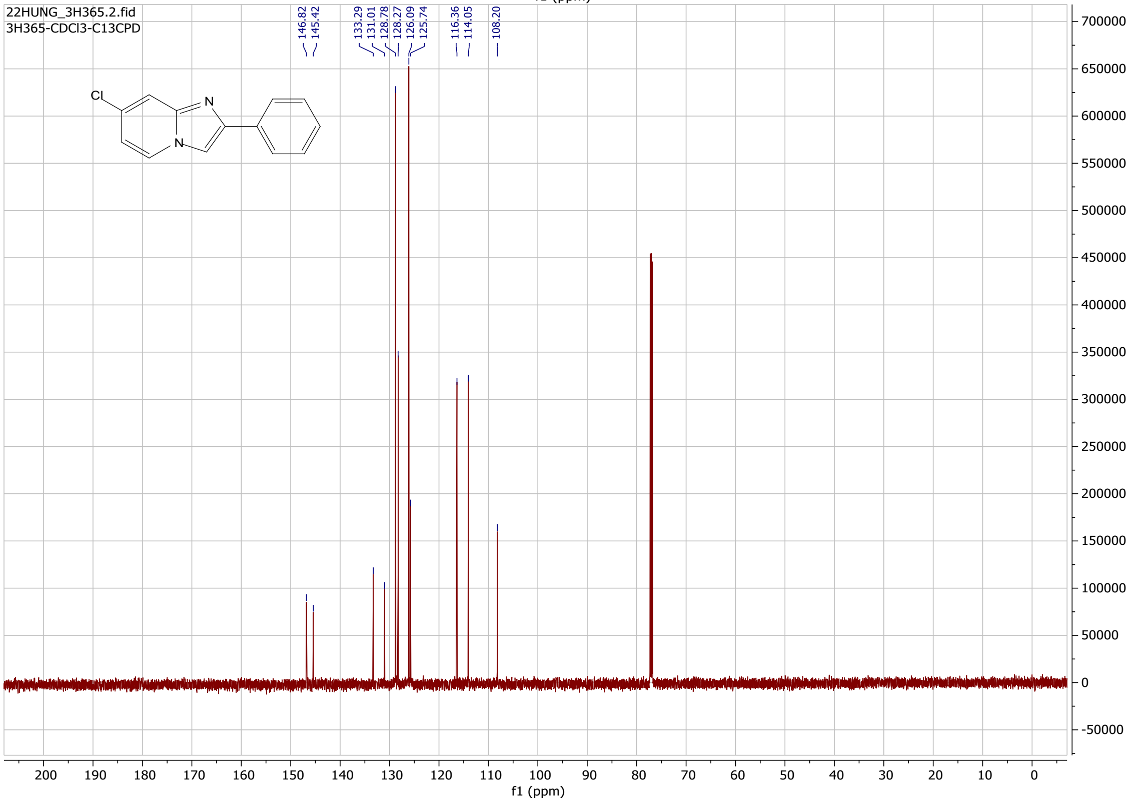

(2-phenylimidazo[1,2-a]pyridin-3-yl)(pyridin-2-yl)methanone 3a

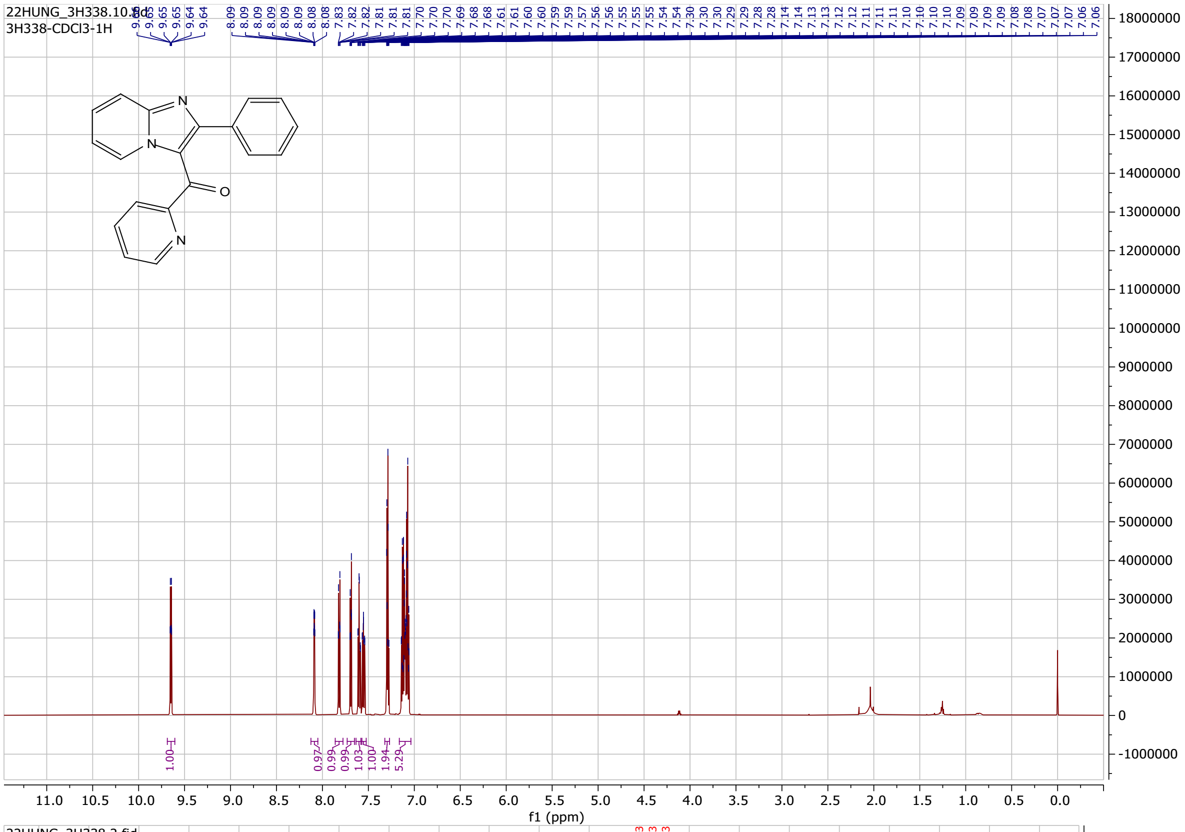

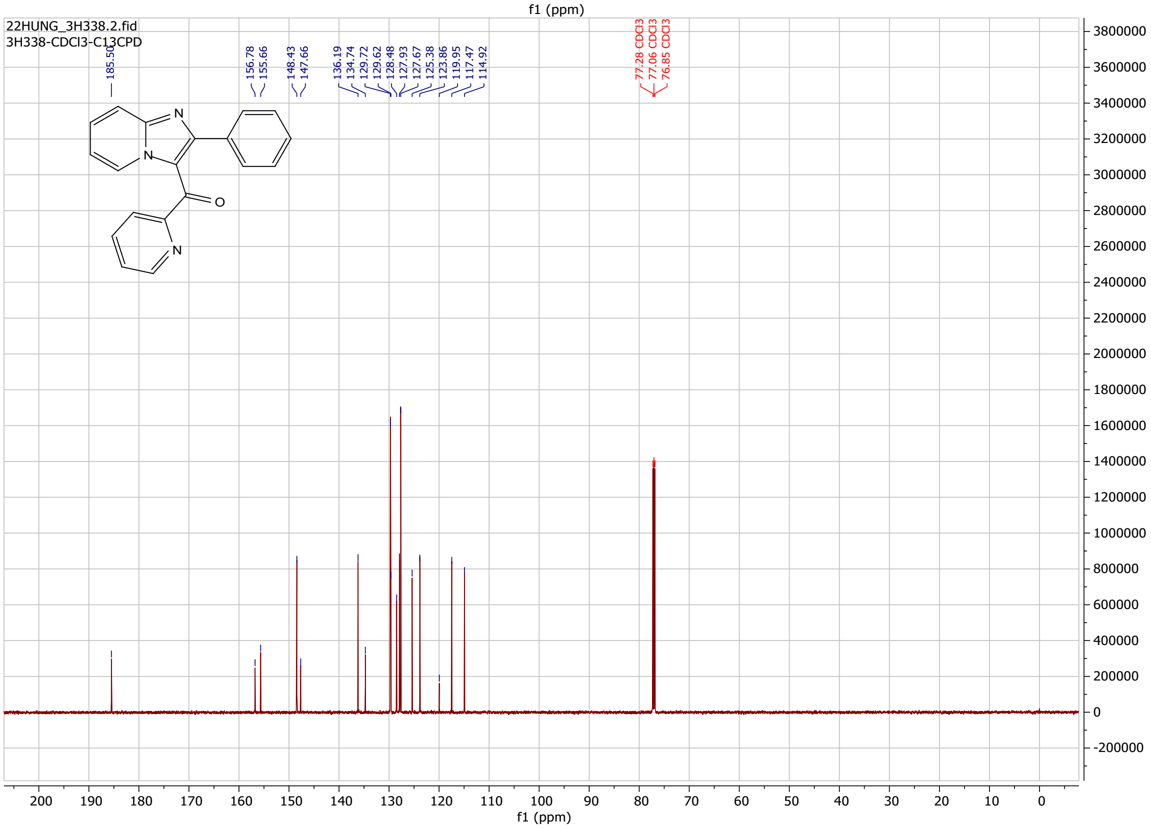

Pyridin-2-yl(2-(p-tolyl)imidazo[1,2-a]pyridin-3-yl)methanone 3b

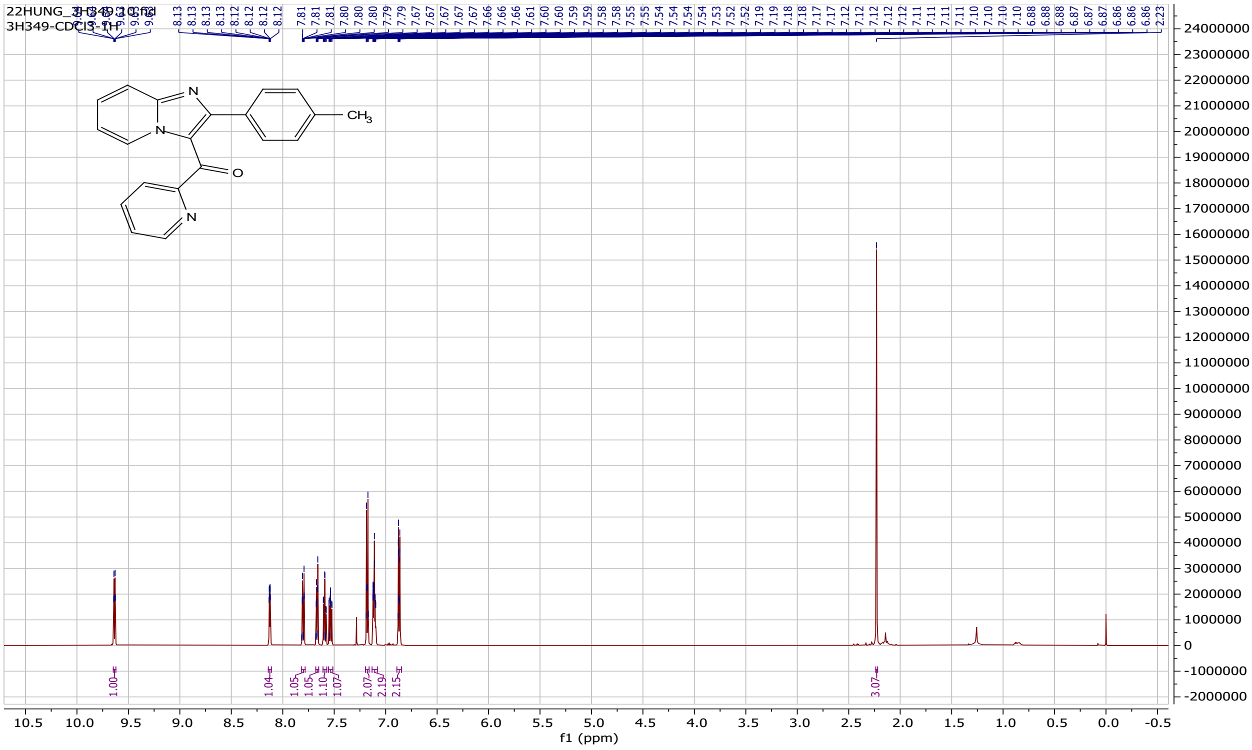
N
CH3
O
f1 (ppm)

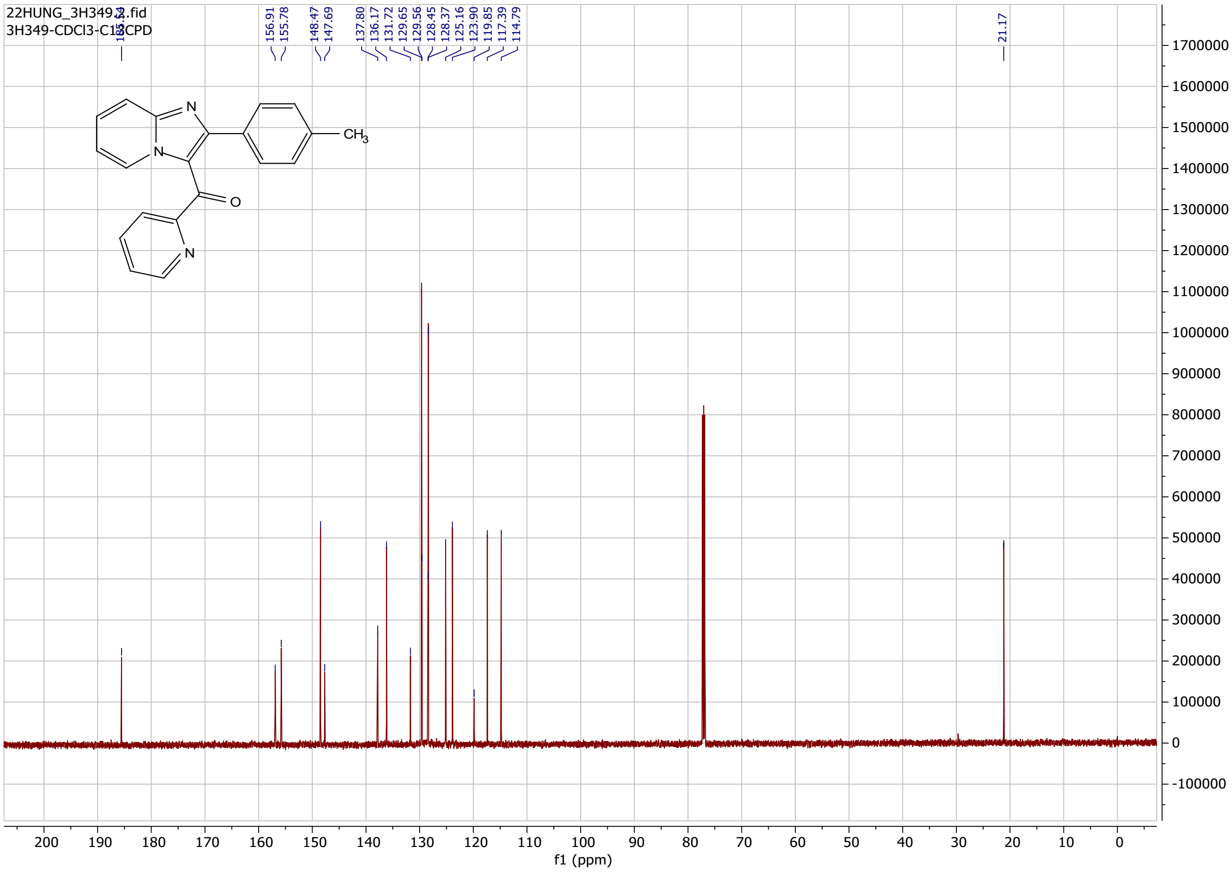
22HUNG_3H349.2.fid
3H349-CDCl3-C13CPD
156.91
155.78
148.47
147.69
137.80
136.17
131.72
129.65
129.56
128.45
128.37
125.16
123.90
119.85
117.39
114.79
21.17
N
CH3
O
f1 (ppm)

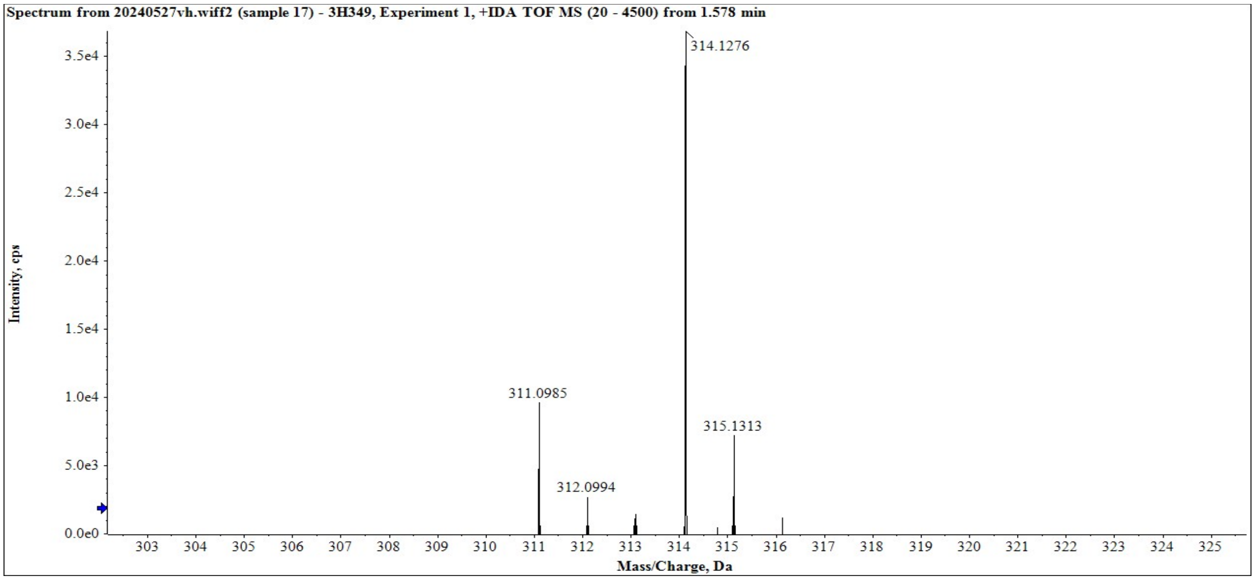

(2-(4-ethylphenyl)imidazo[1,2-a]pyridin-3-yl)(pyridin-2-yl)methanone 3c

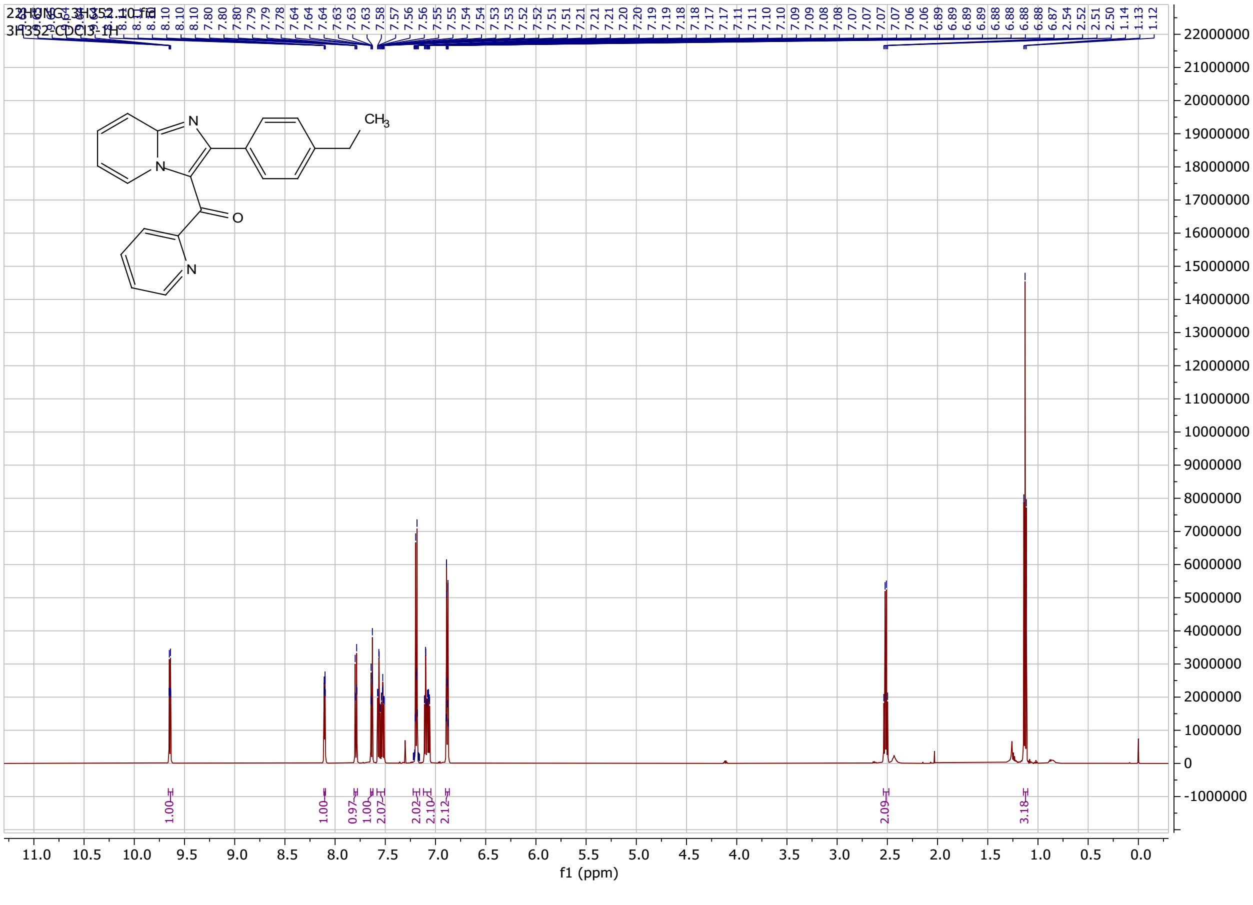

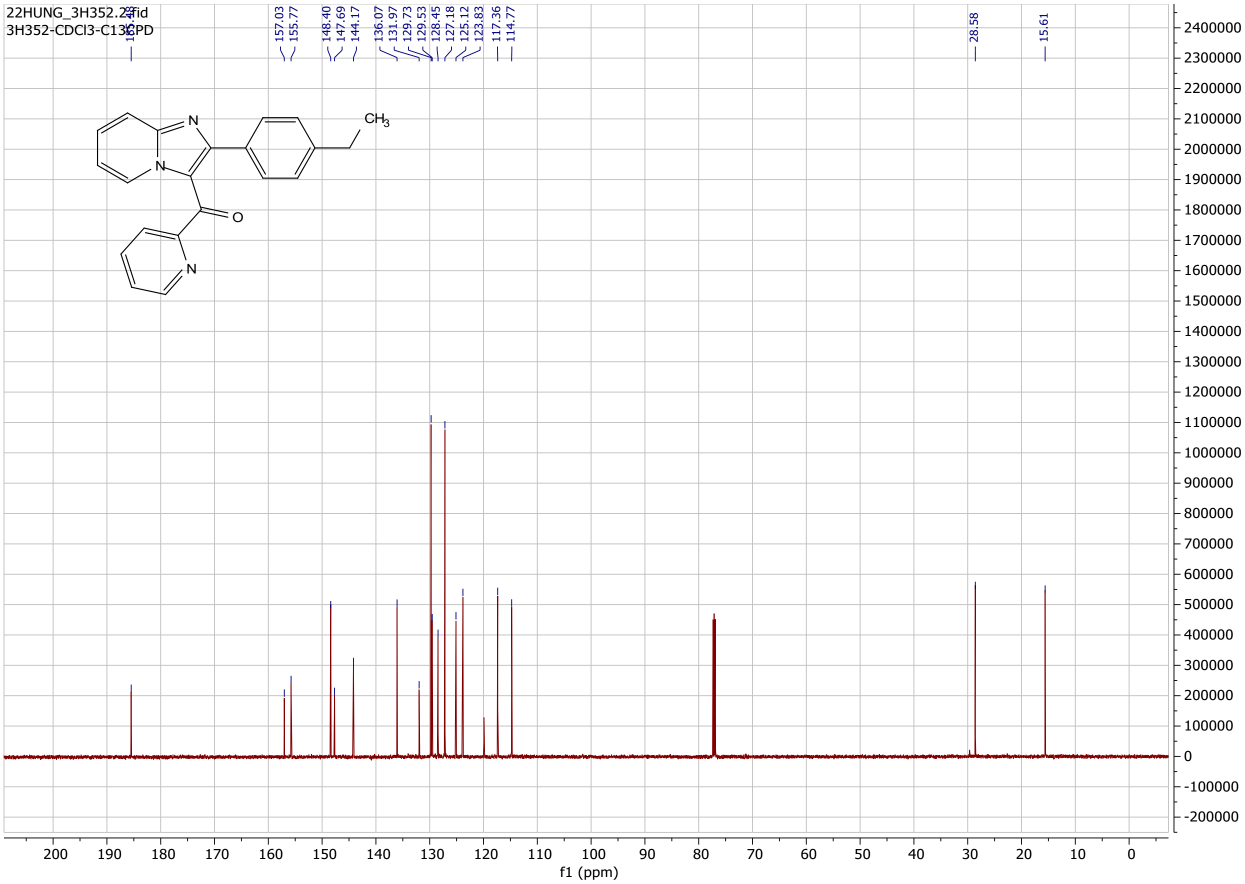
22HUNG_3H352.2.fid
3H352-CDCl3-C13CPD
157.03
155.77
148.40
147.69
144.17
136.07
131.97
129.73
129.53
128.45
127.18
125.12
123.83
117.36
114.77
28.58
15.61
N
N
CH3
O
N
f1 (ppm)

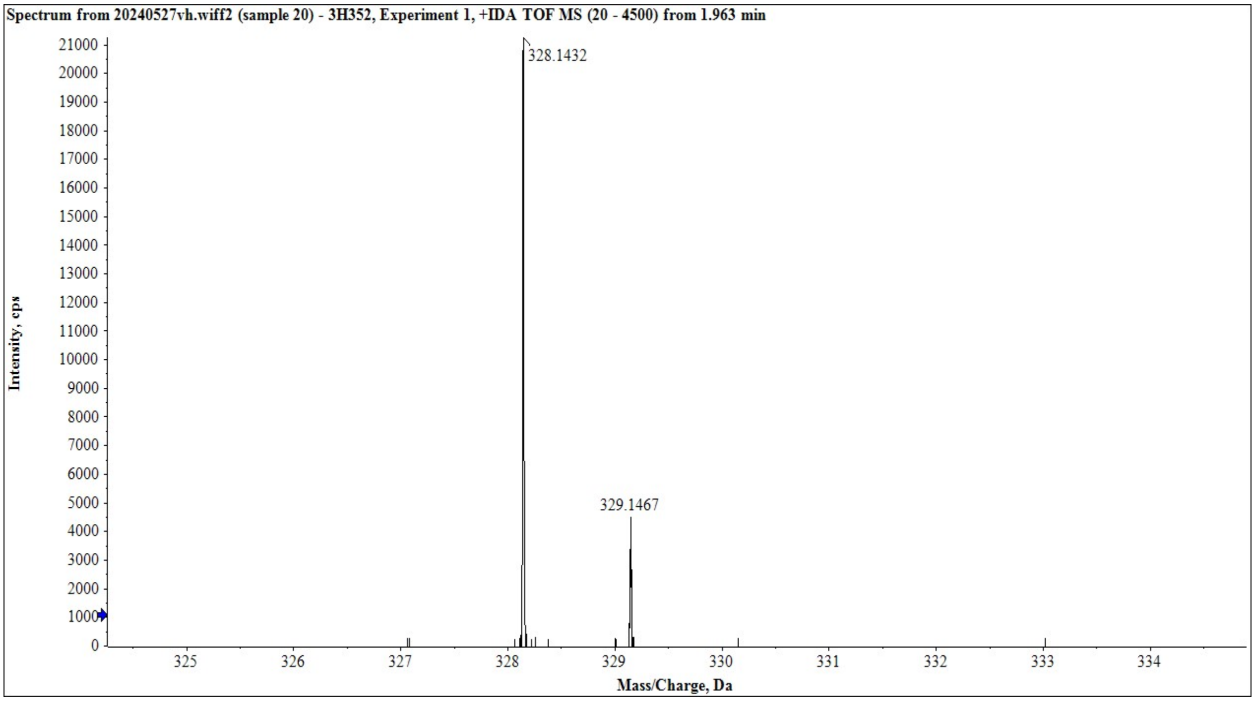
Spectrum from 20240527vh.wiff2 (sample 20) - 3H352, Experiment 1, +IDA TOF MS (20 - 4500) from 1.963 min
328.1432
329.1467
Intensity, cps
Mass/Charge, Da

(2-(4-methoxyphenyl)imidazo[1,2-a]pyridin-3-yl)(pyridin-2-yl)methanone 3d

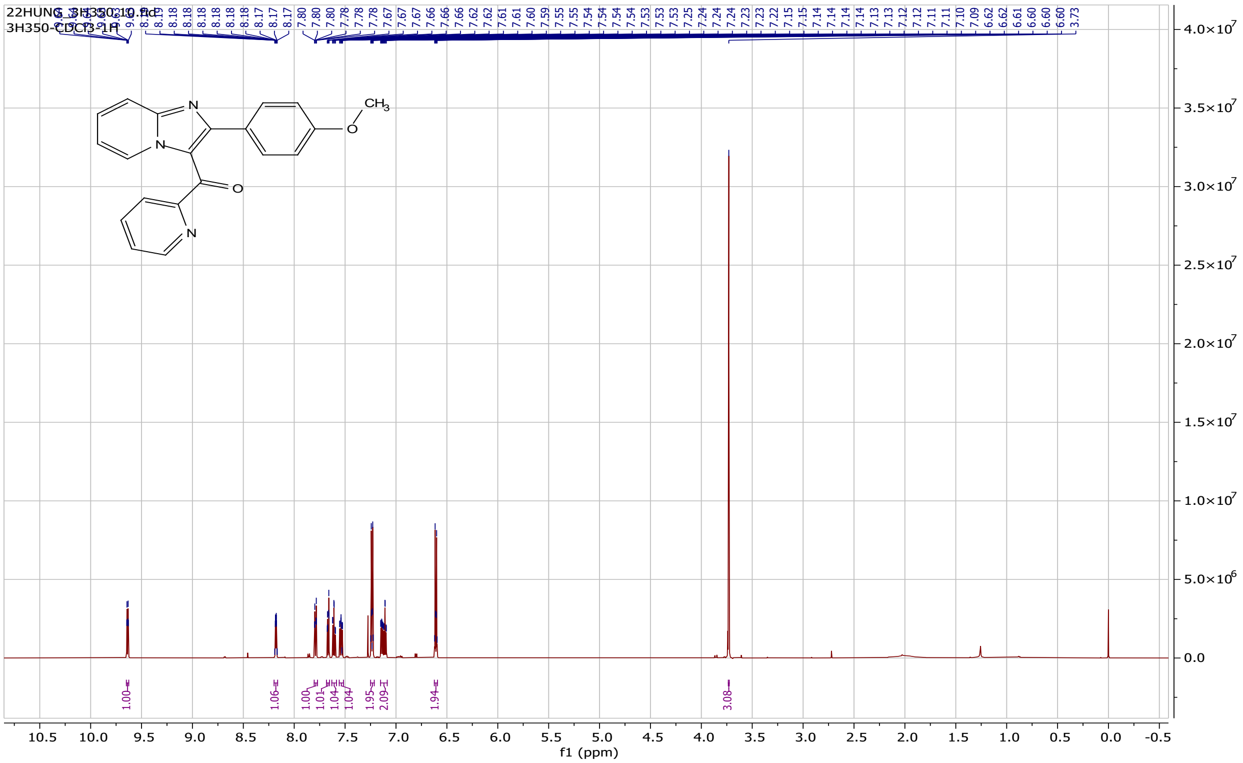

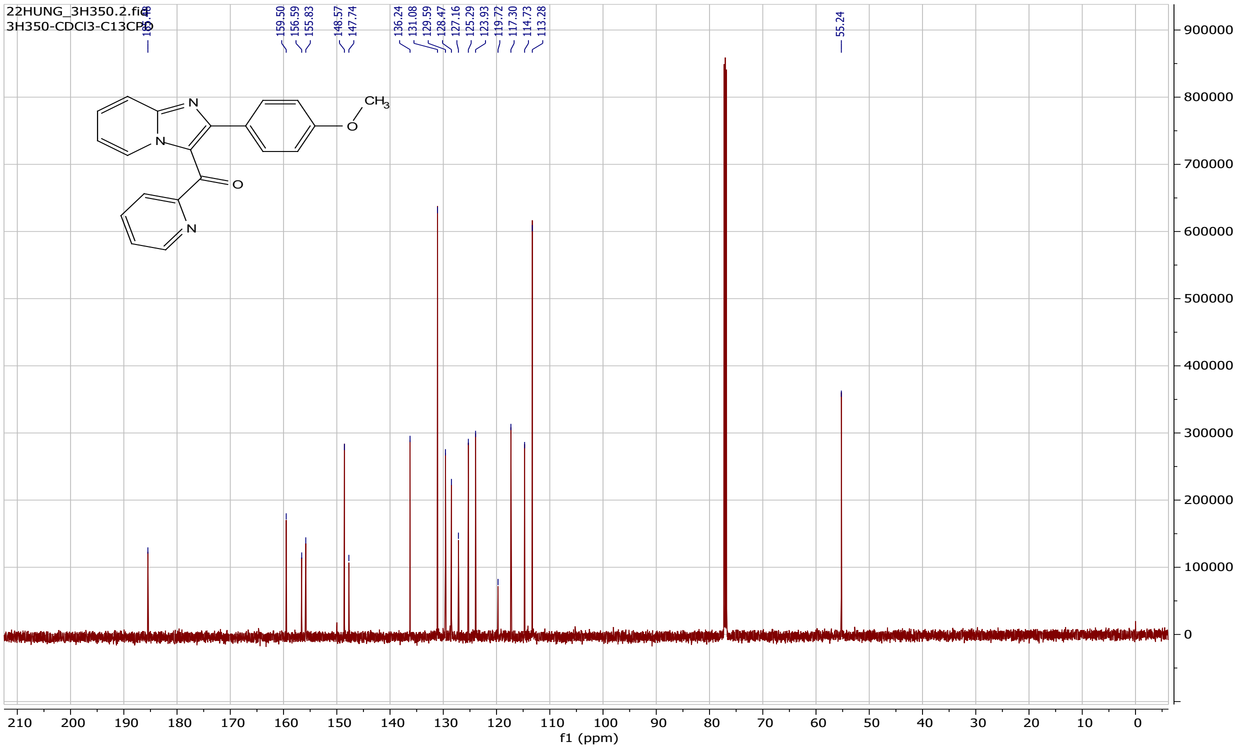

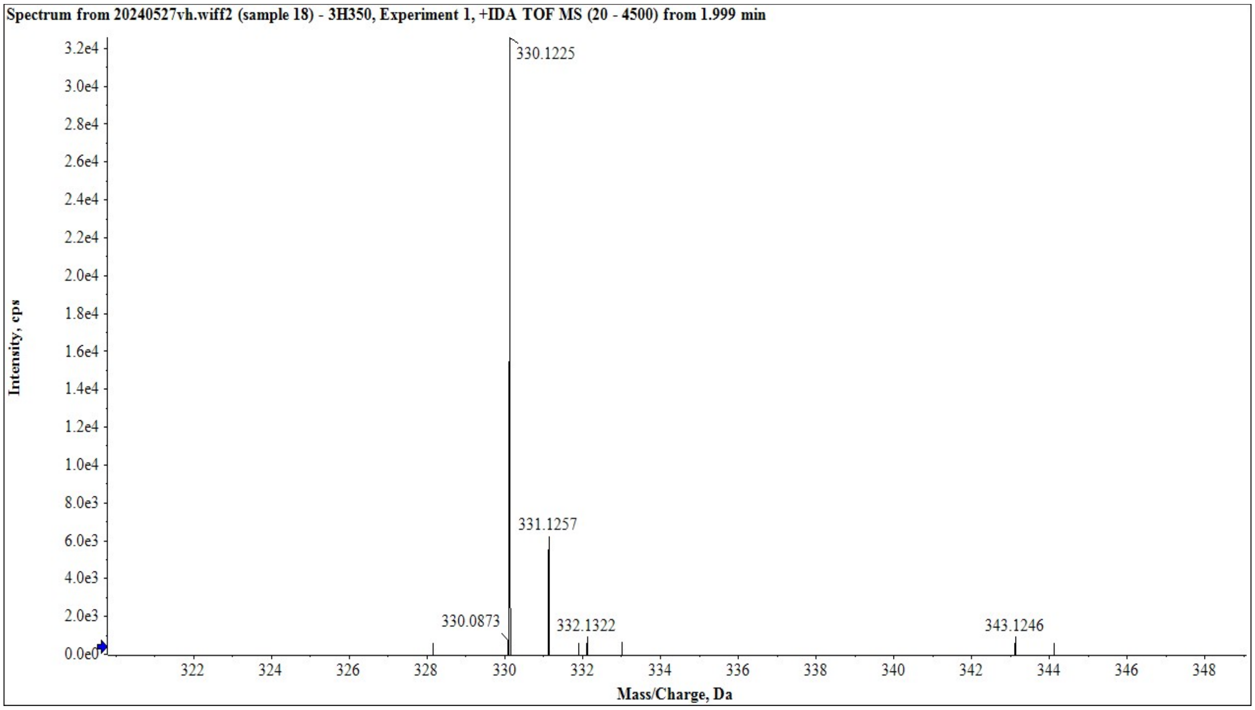

(2-(4-fluorophenyl)imidazo[1,2-a]pyridin-3-yl)(pyridin-2-yl)methanone 3e

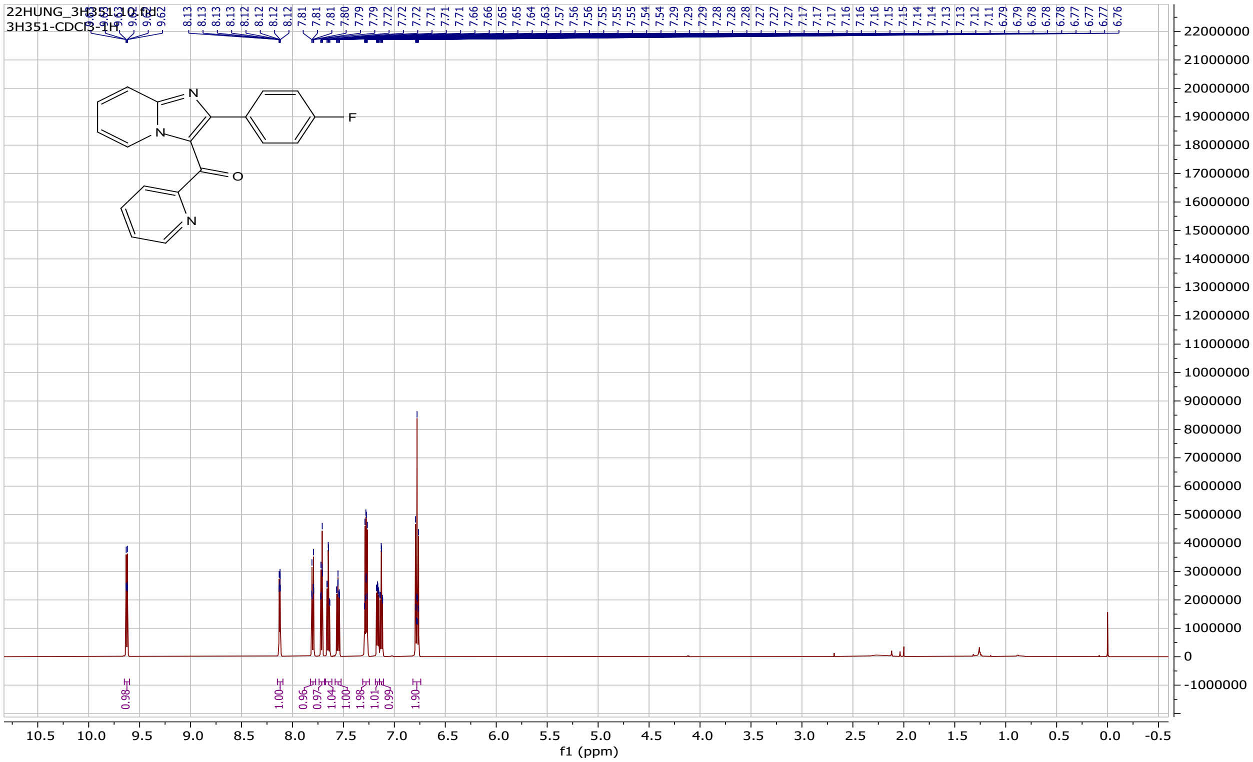

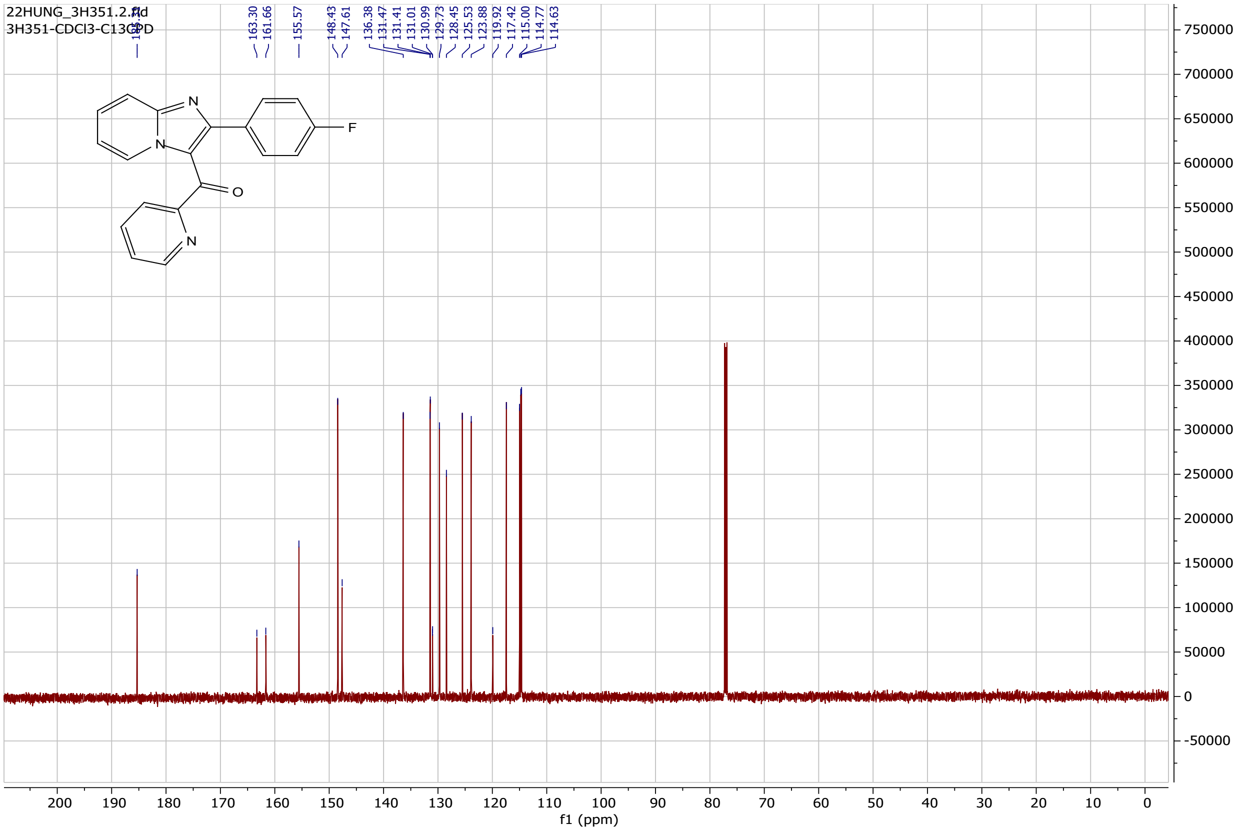
22HUNG_3H351.2.fid
3H351-CDCl3-C13CPD
163.30
161.66
155.57
148.43
147.61
136.38
131.47
131.41
131.01
130.99
129.73
128.45
125.53
123.88
119.92
117.42
115.00
114.77
114.63
N
N
F
O
N
f1 (ppm)

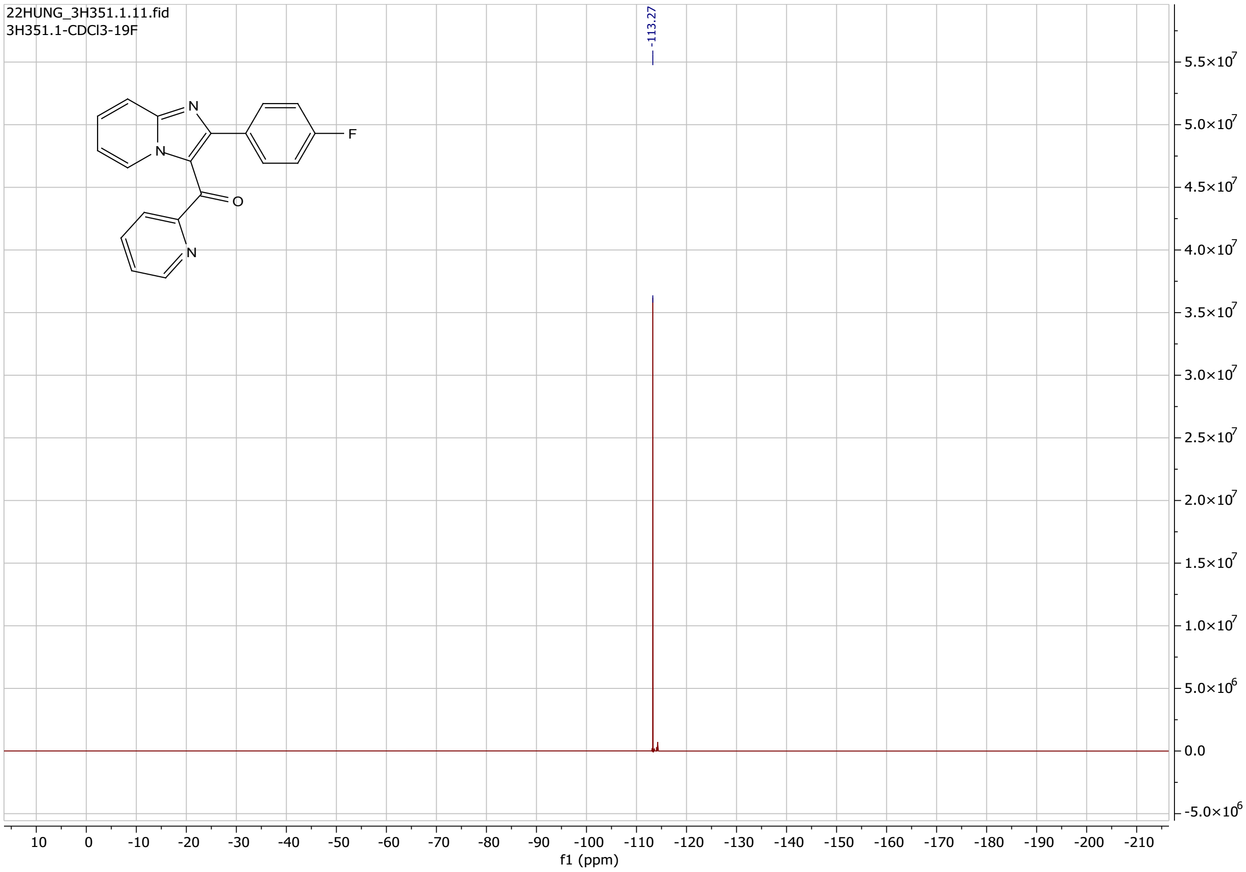
22HUNG_3H351.1.11.fid
3H351.1-CDCl3-19F
-113.27
N
N
F
O
N
f1 (ppm)

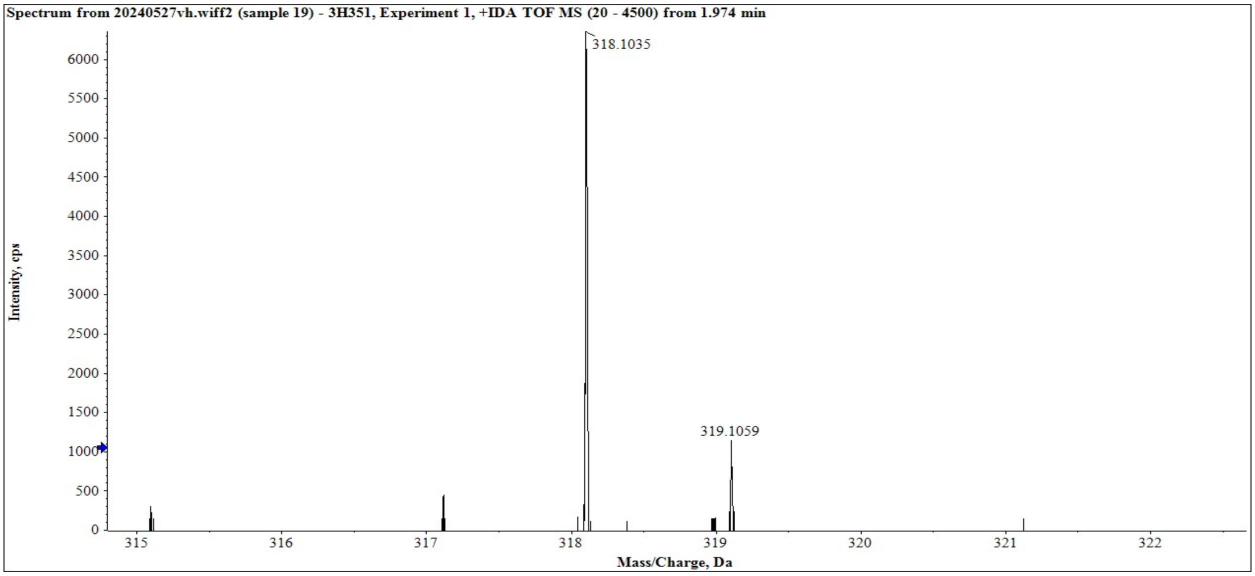

(2-(4-chlorophenyl)imidazo[1,2-a]pyridin-3-yl)(pyridin-2-yl)methanone 3f

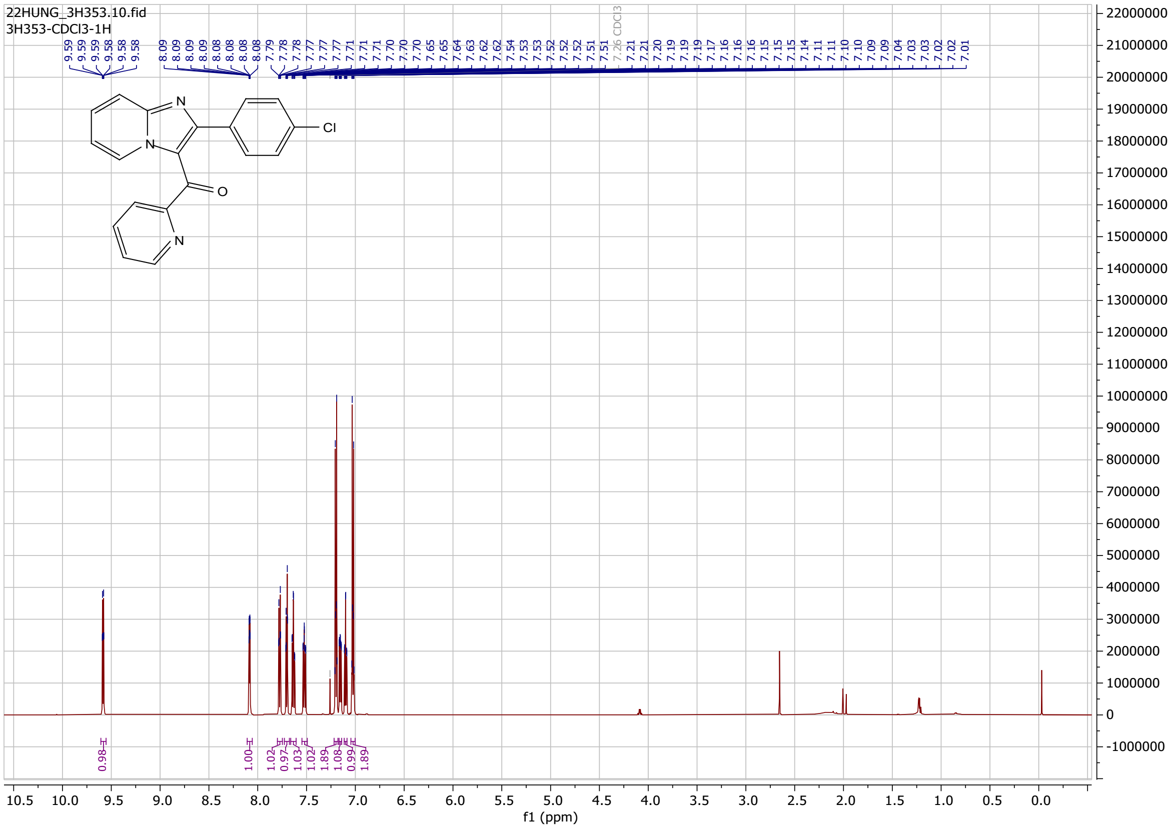

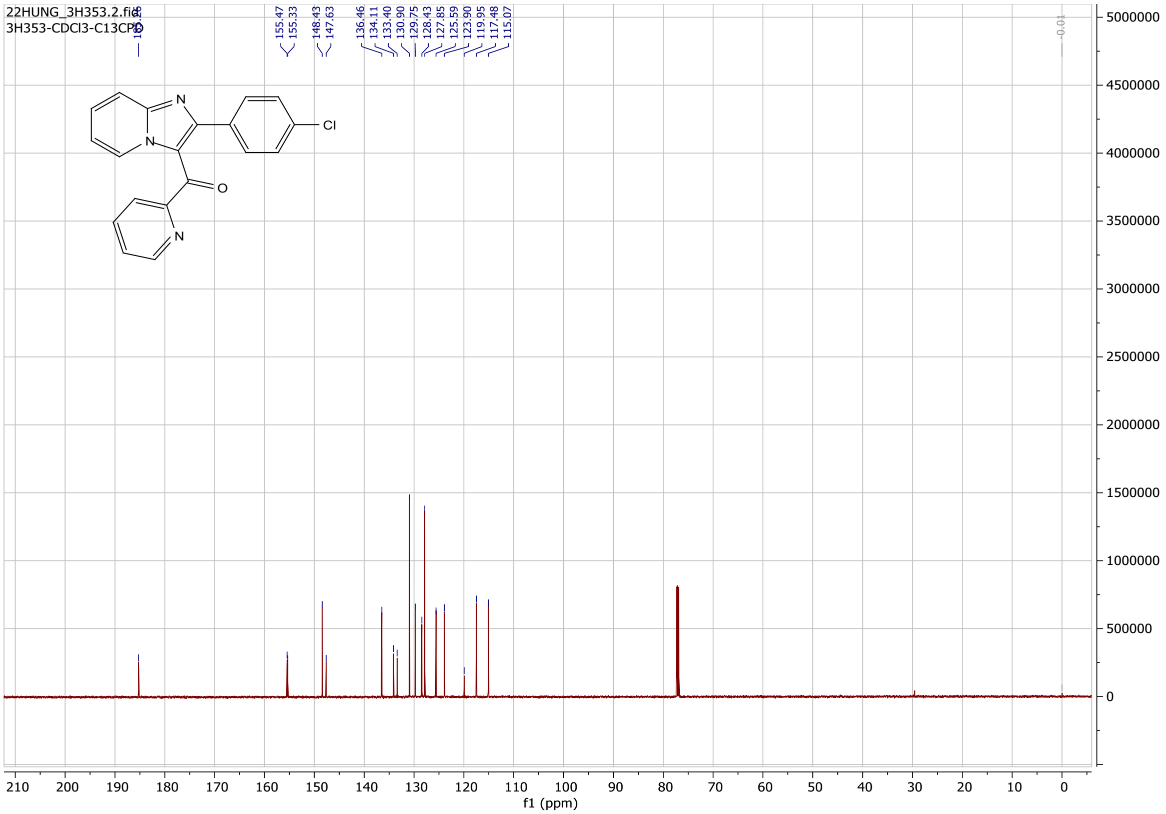

(2-phenylimidazo[1,2-a]pyridin-3-yl)(pyridin-4-yl)methanone 3g

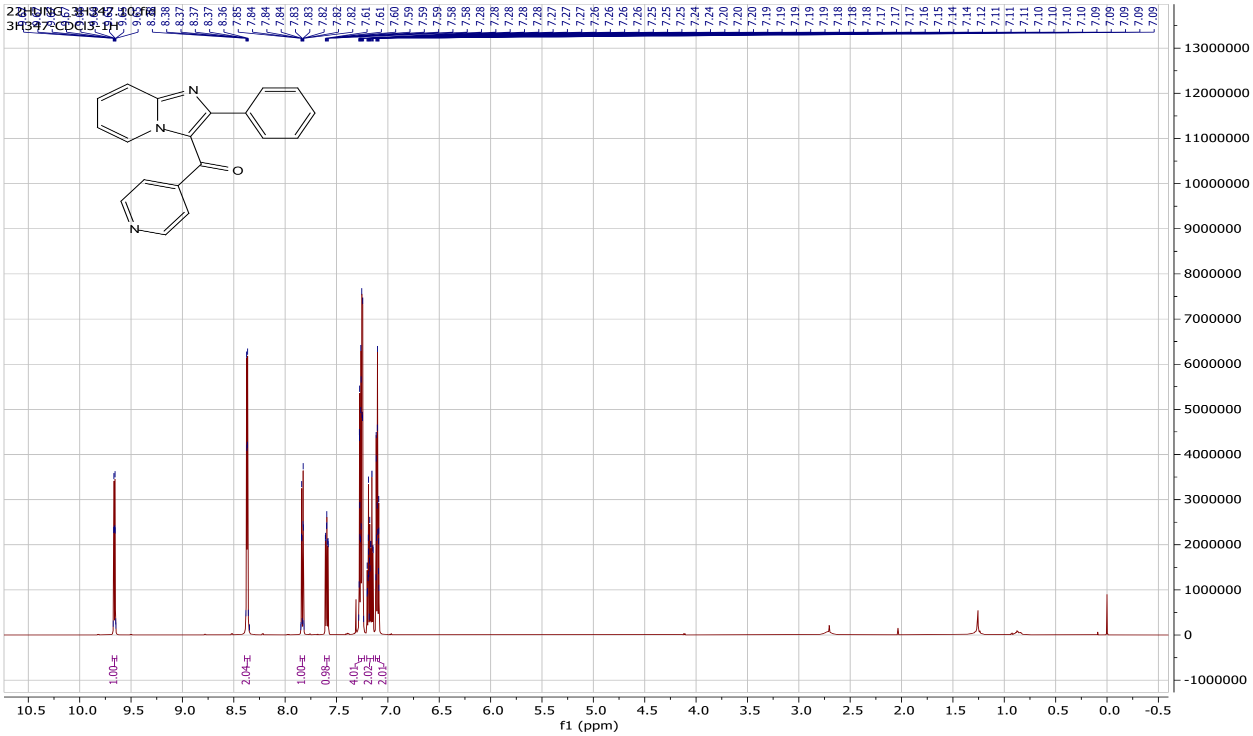

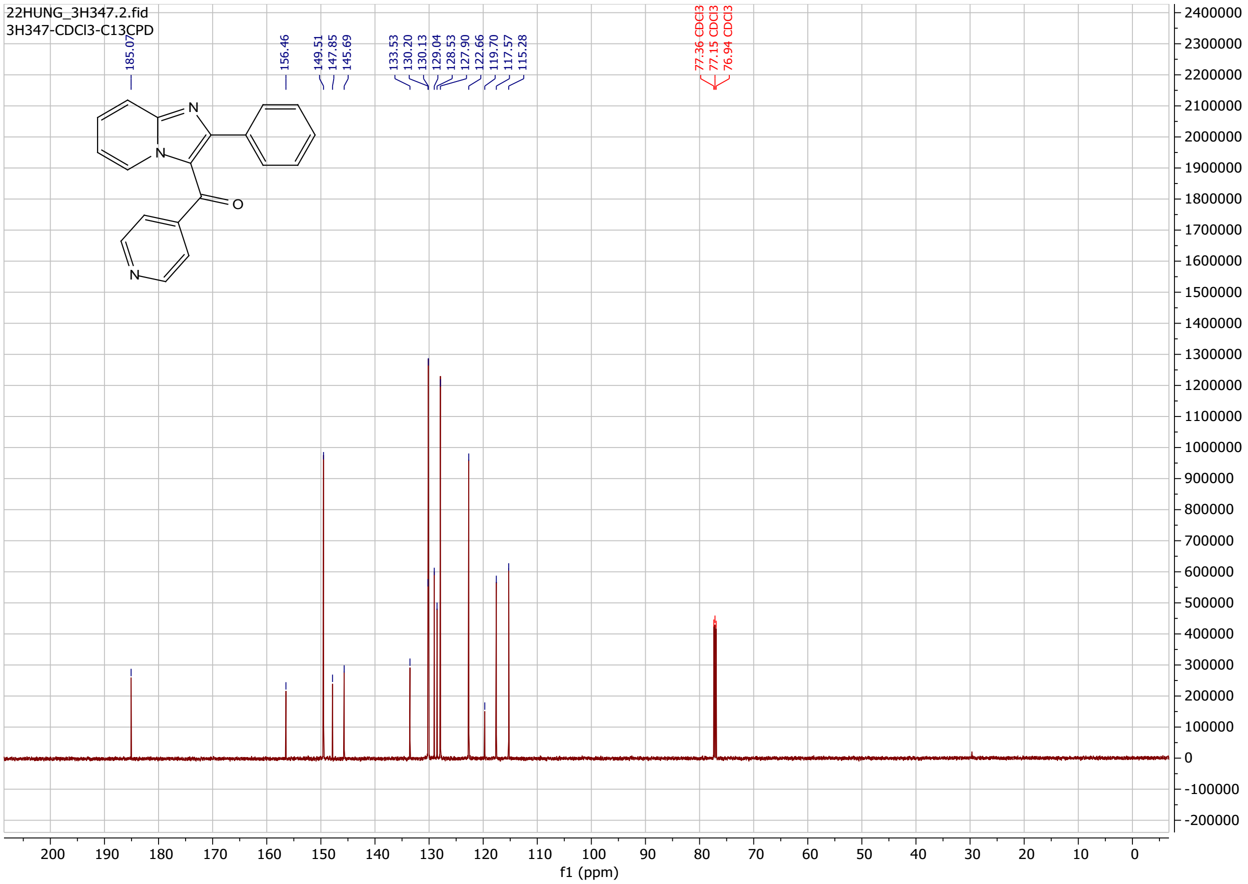
22HUNG_3H347.2.fid
3H347-CDCl3-C13CPD
185.07
156.46
149.51
147.85
145.69
133.53
130.20
130.13
129.04
128.53
127.90
122.66
119.70
117.57
115.28
77.36 CDCl3
77.15 CDCl3
76.94 CDCl3
N
N
O
N
f1 (ppm)

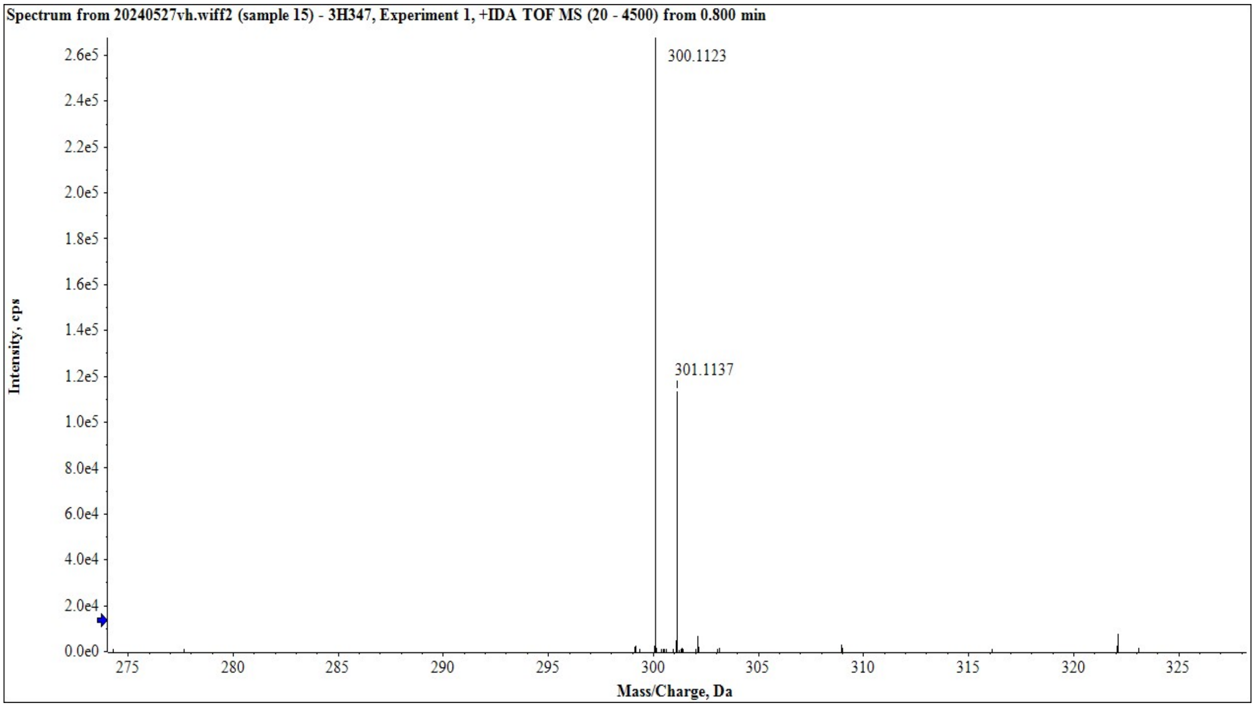
Spectrum from 20240527vh.wiff2 (sample 15) - 3H347, Experiment 1, +IDA TOF MS (20 - 4500) from 0.800 min
300.1123
301.1137
Intensity, cps
Mass/Charge, Da

pyridin-4-yl(2-(p-tolyl)imidazo[1,2-a]pyridin-3-yl)methanone 3h

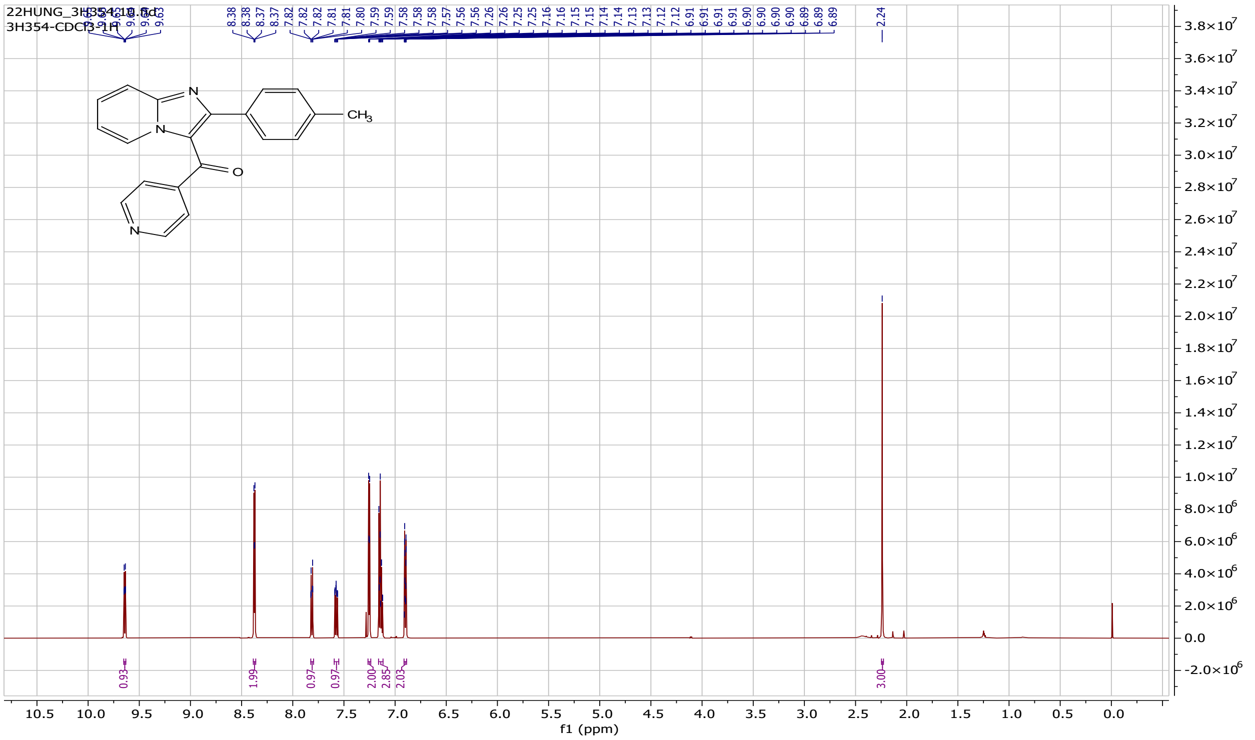

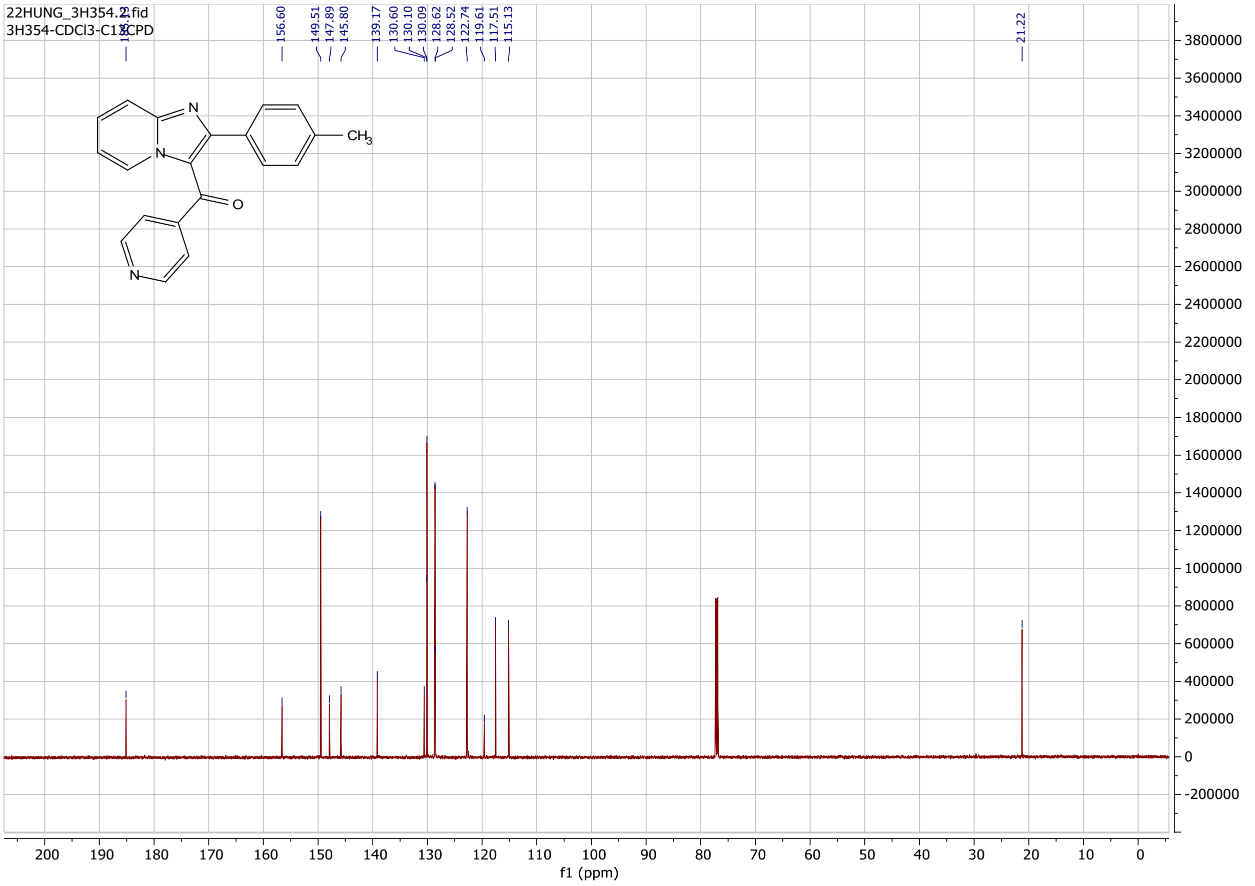

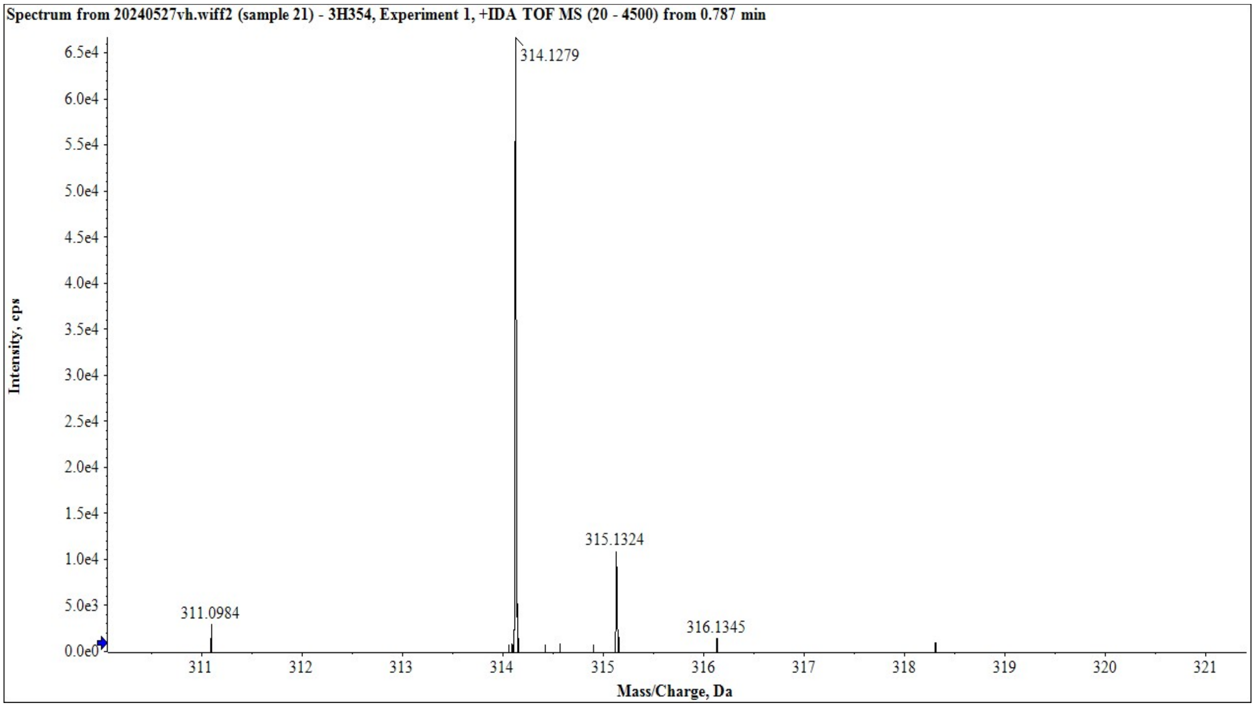

(2-phenylimidazo[1,2-a]pyridin-3-yl)(quinolin-4-yl)methanone 3i

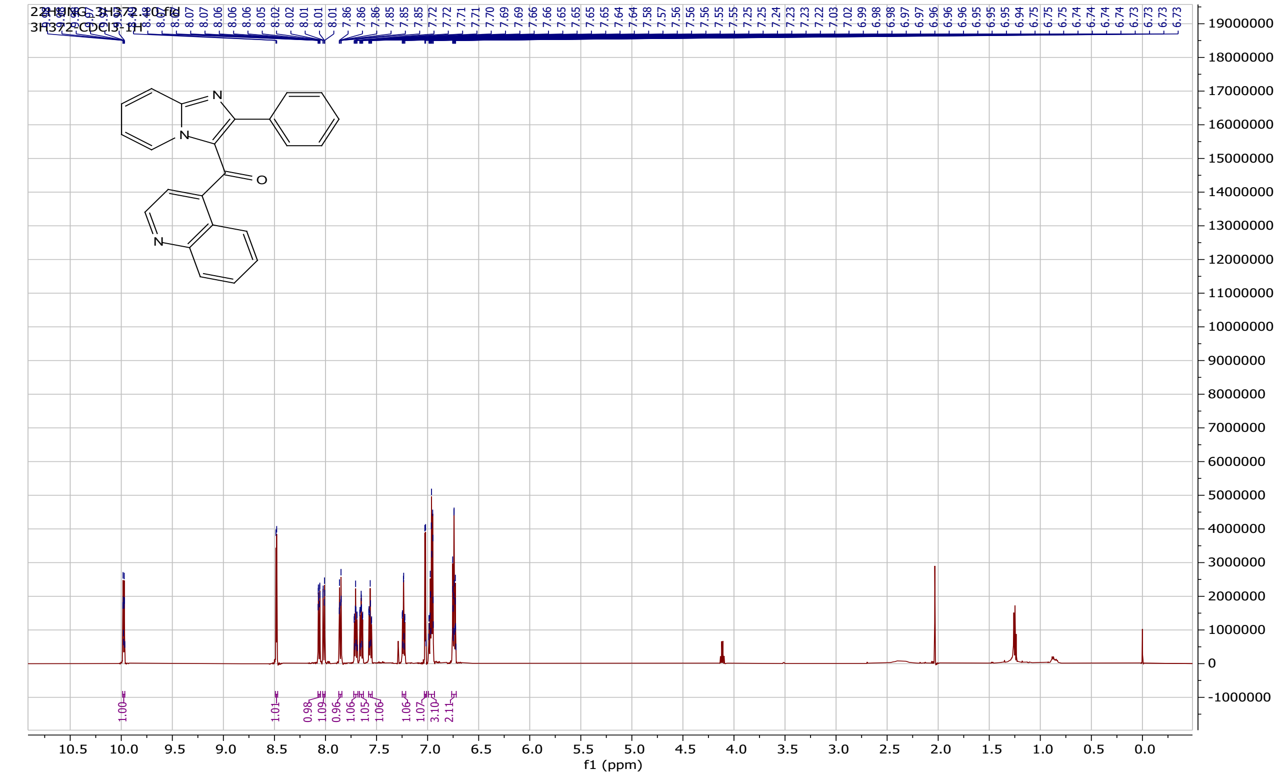

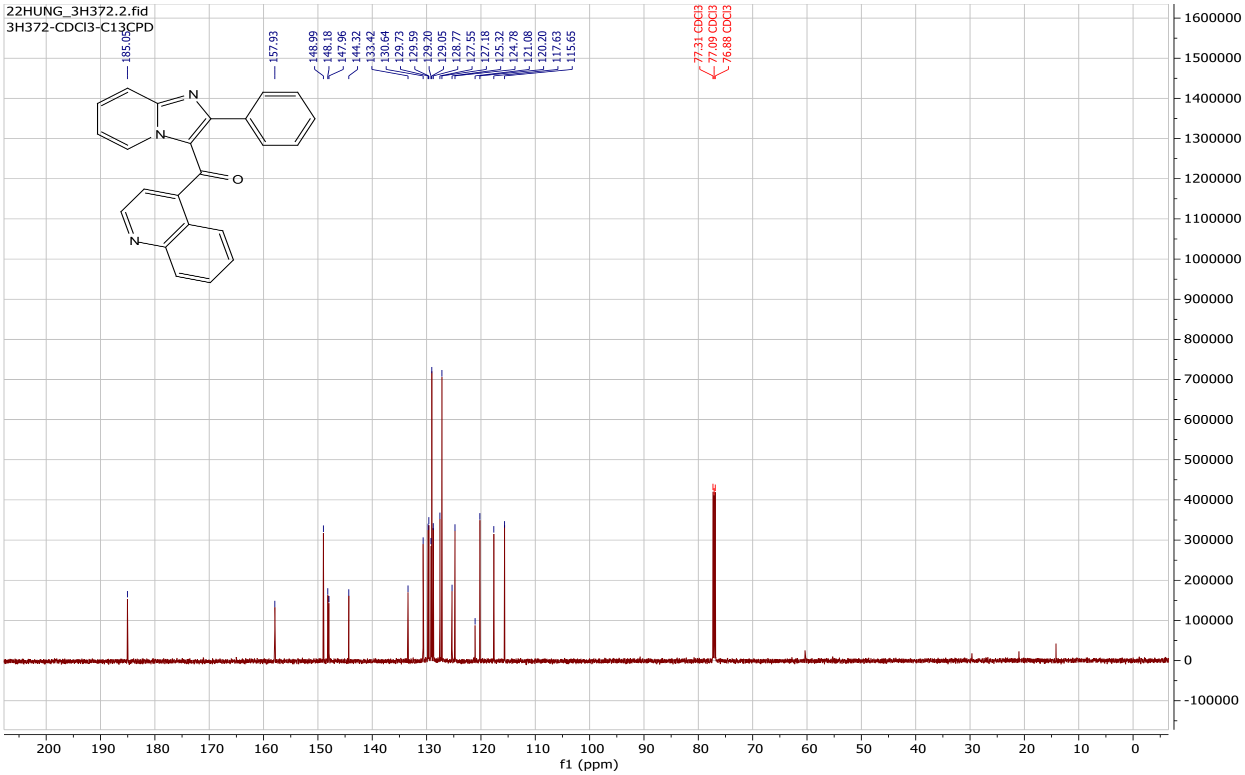
22HUNG_3H372.2.fid
3H372-CDCl3-C13CPD
185.05
157.93
148.99
148.18
147.96
144.32
133.42
130.64
129.73
129.59
129.20
129.05
128.77
127.55
127.18
125.32
124.78
121.08
120.20
117.63
115.65
77.31 CDCl3
77.09 CDCl3
76.88 CDCl3
N
N
O
N
f1 (ppm)

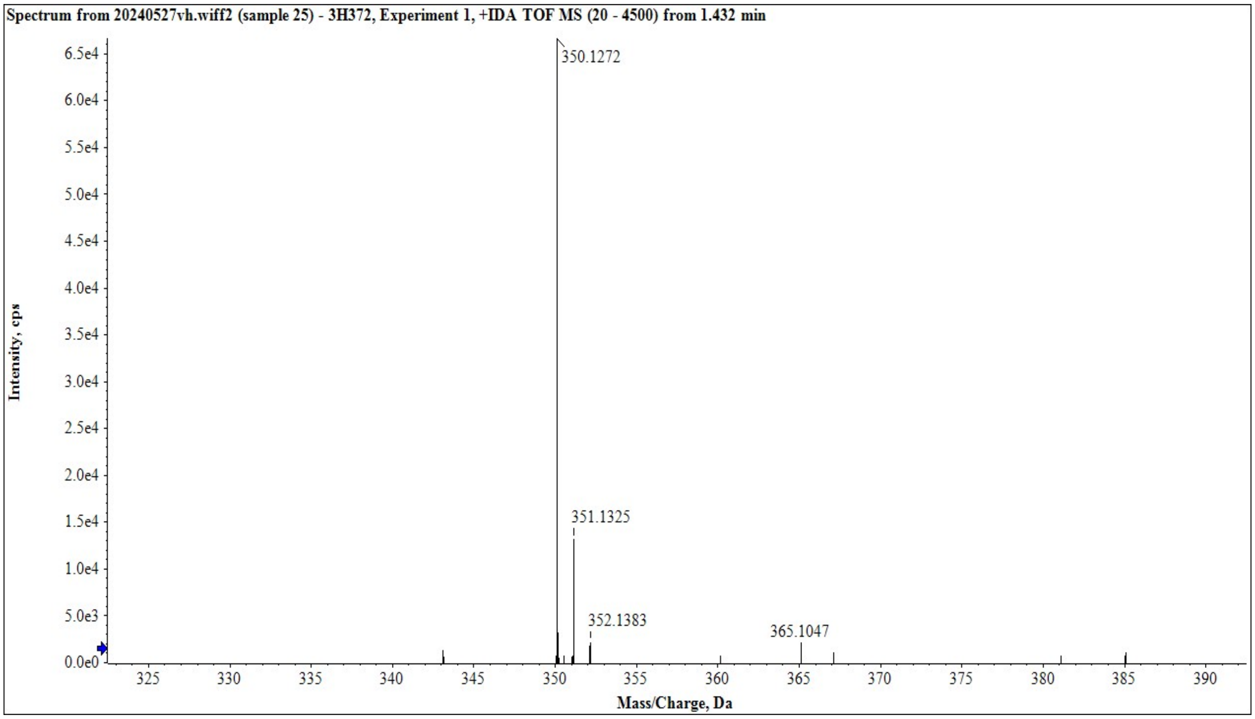
Spectrum from 20240527vh.wiff2 (sample 25) - 3H372, Experiment 1, +IDA TOF MS (20 - 4500) from 1.432 min
350.1272
351.1325
352.1383
365.1047
Intensity, cps
Mass/Charge, Da

phenyl(2-phenylimidazo[1,2-a]pyridin-3-yl)methanone 3k

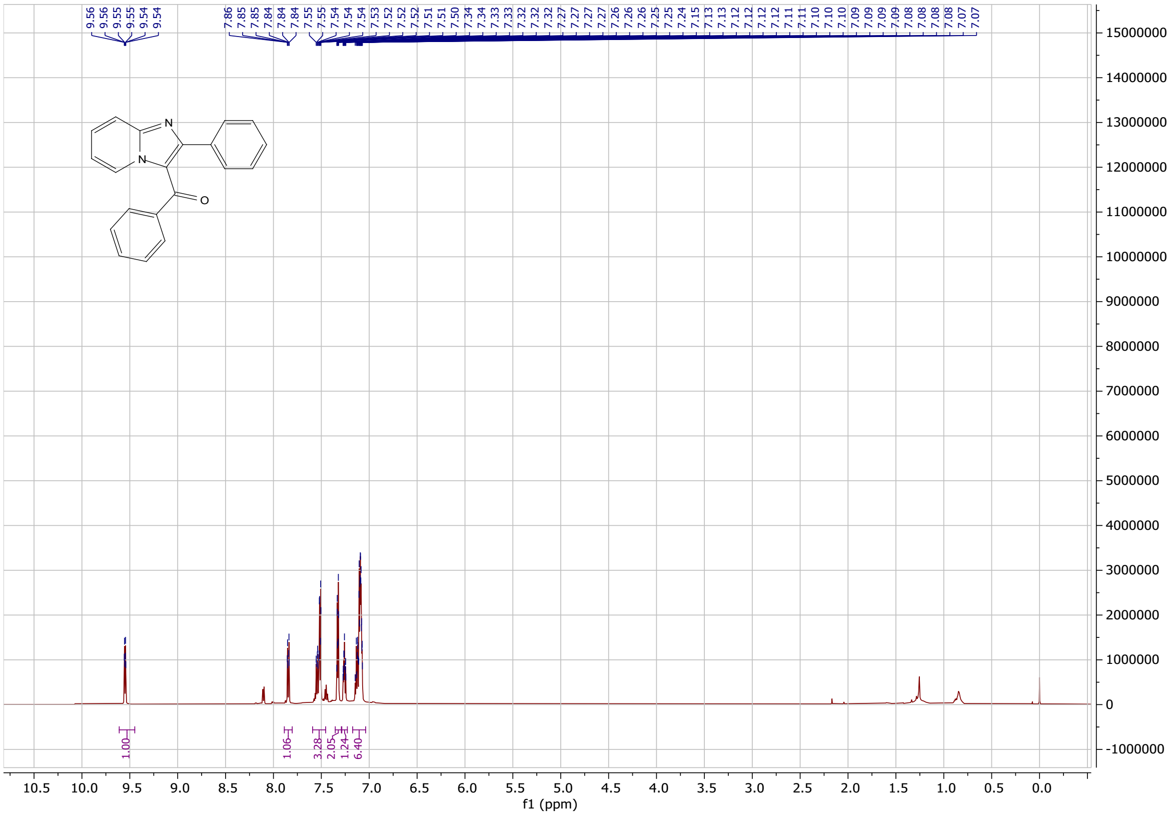

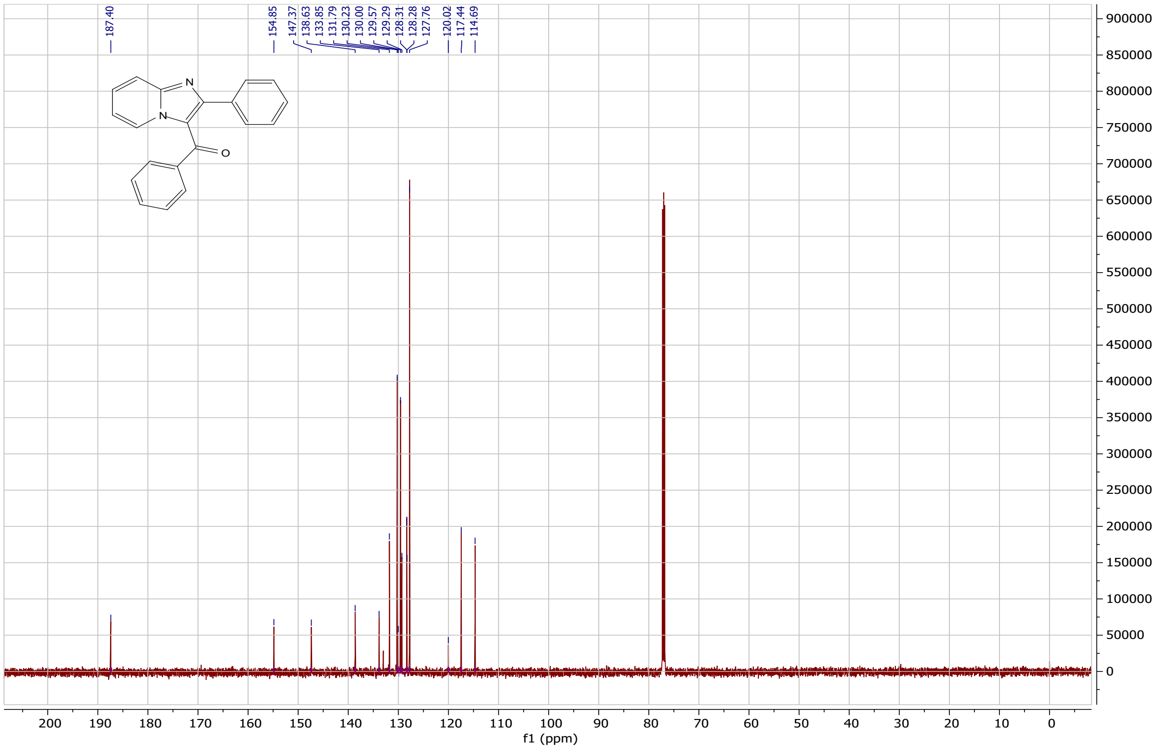

(3-bromophenyl)(2-phenylimidazo[1,2-a]pyridin-3-yl)methanone 3l

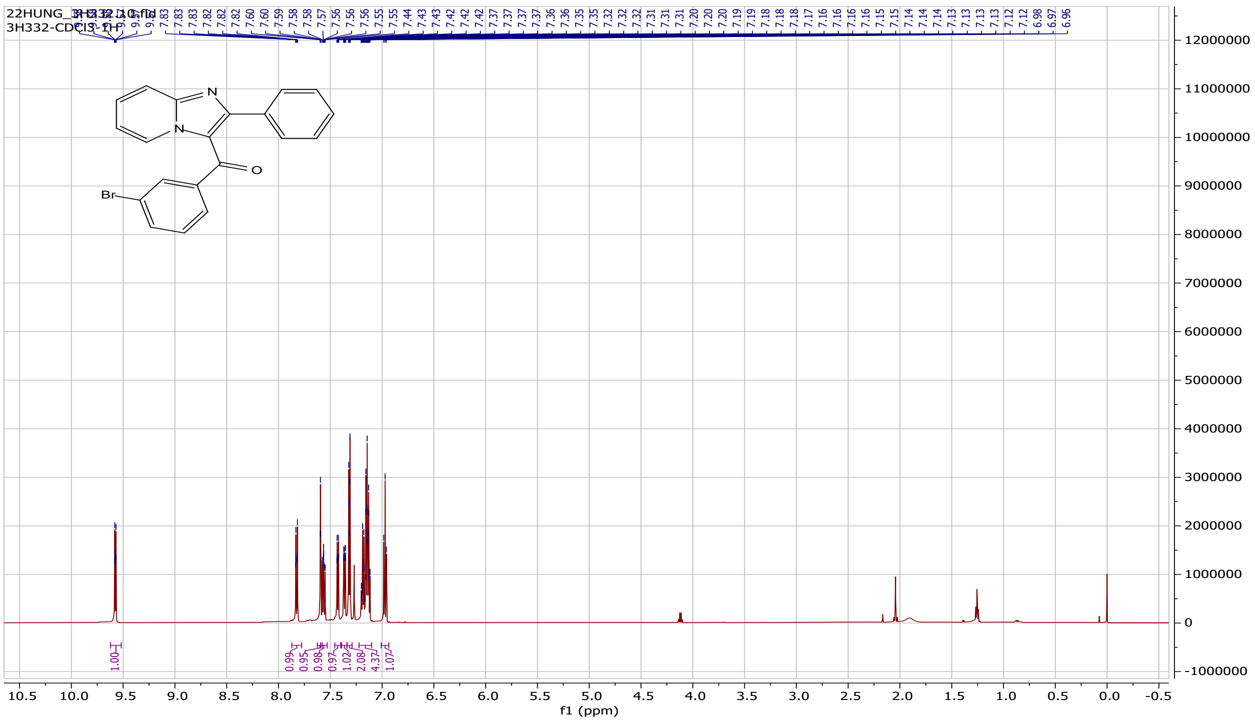

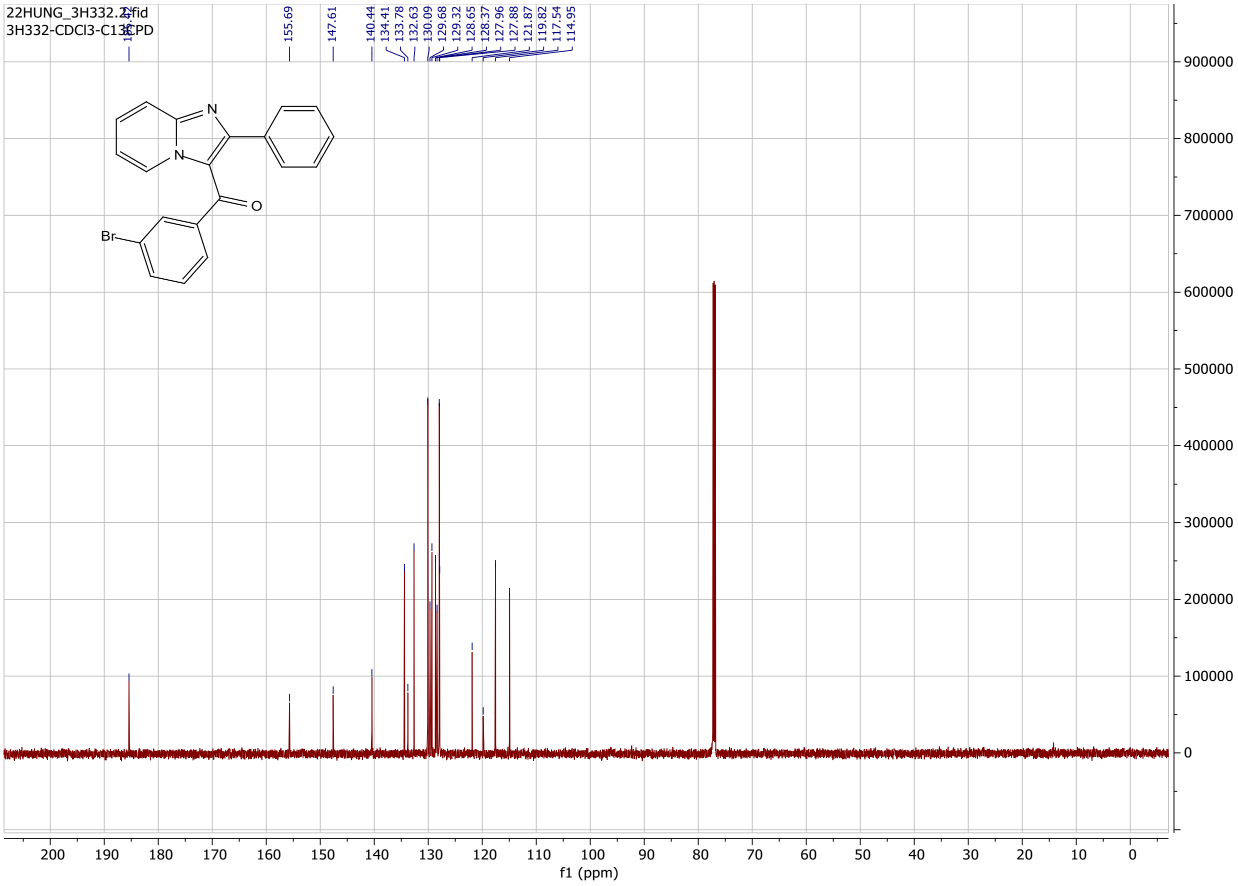

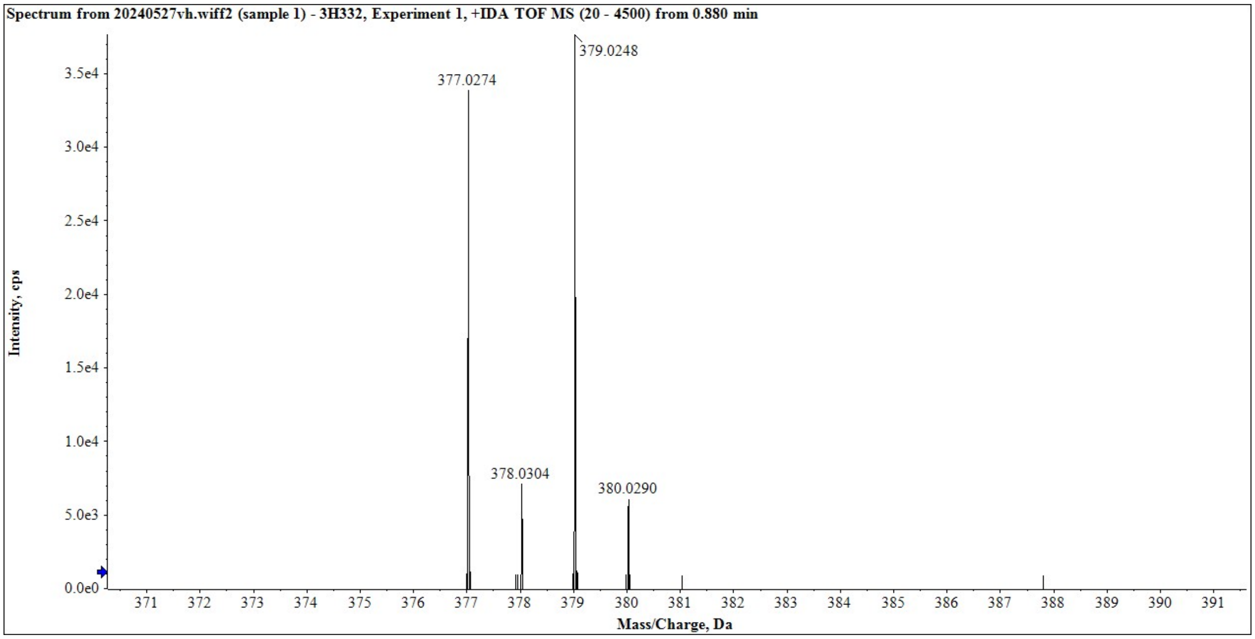

(4-chlorophenyl)(2-phenylimidazo[1,2-a]pyridin-3-yl)methanone 3m

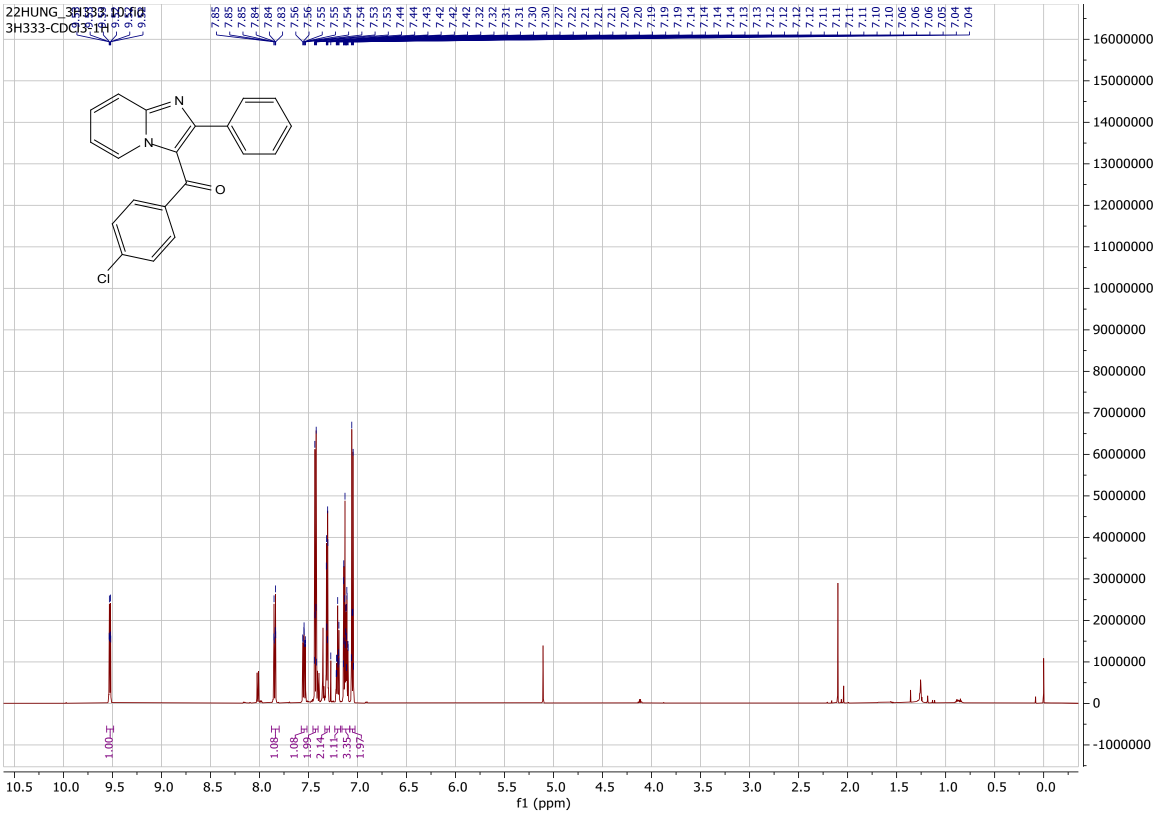

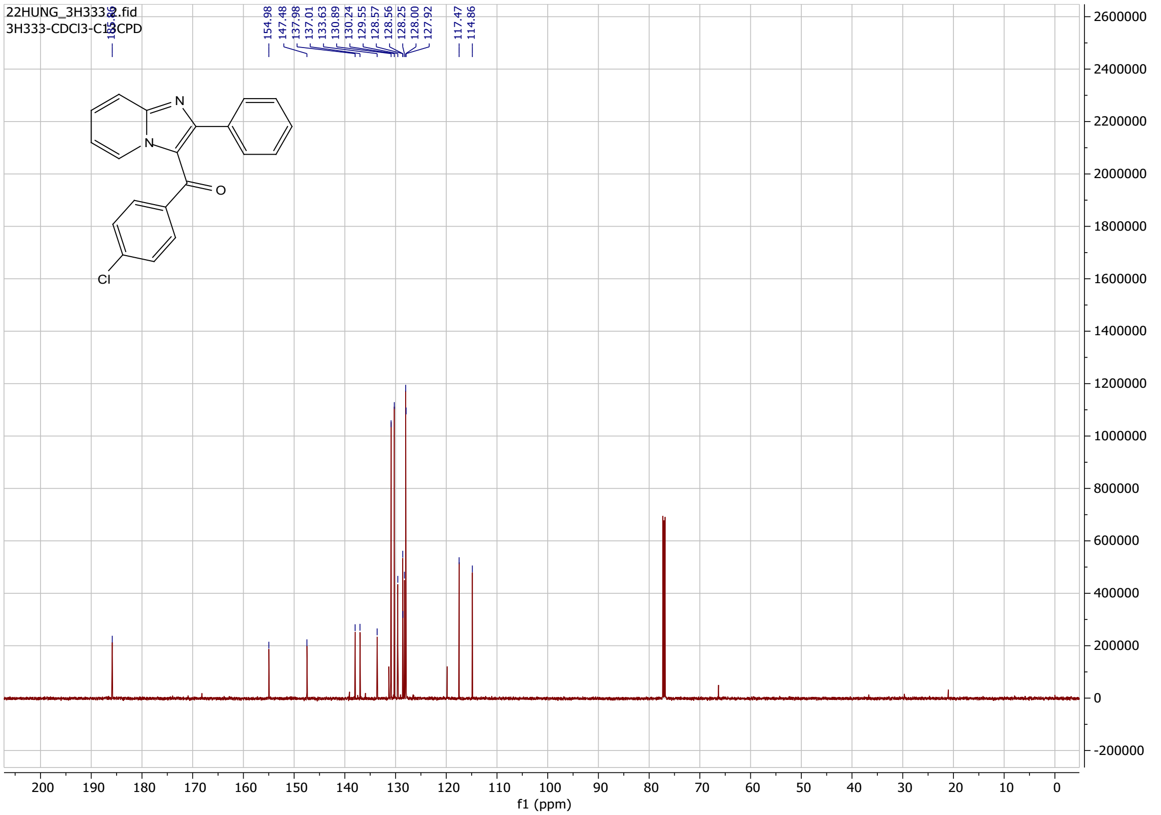

(3-fluorophenyl)(2-phenylimidazo[1,2-a]pyridin-3-yl)methanone 3n

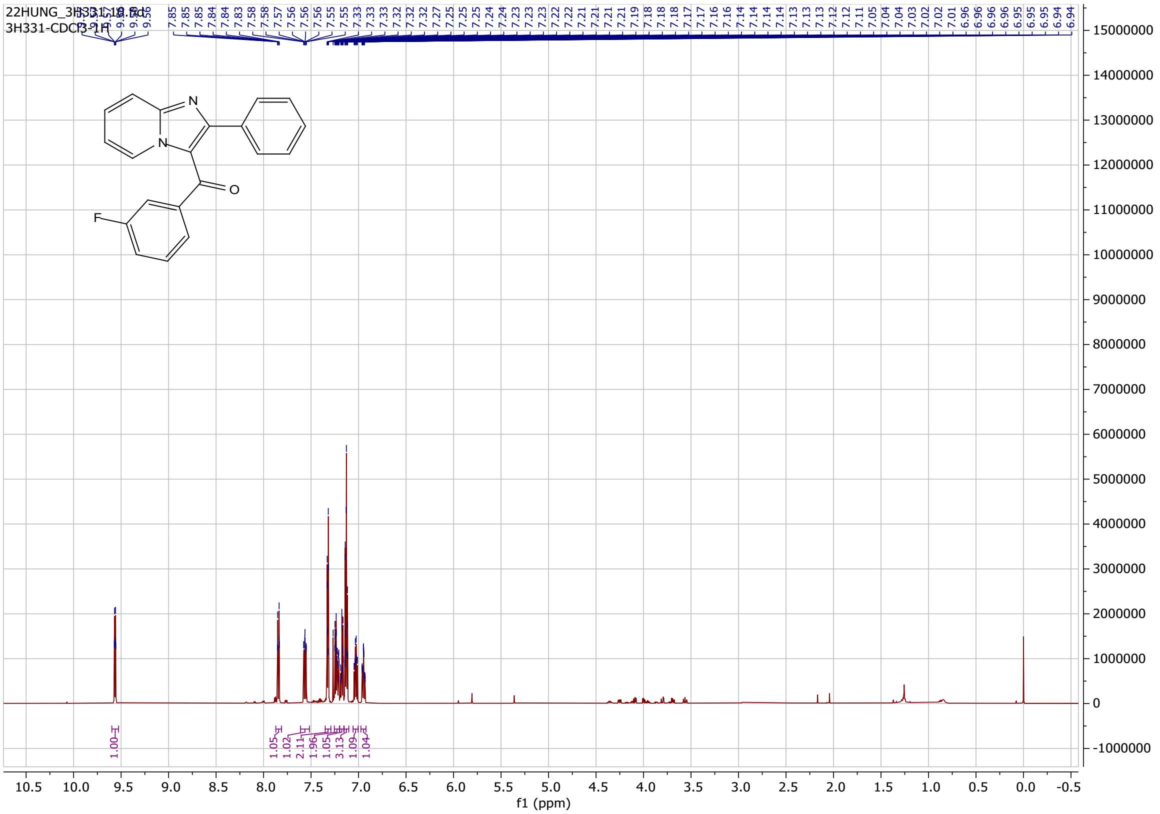
22HUNG_3H331.10.fid
3H331-CDCl3-1H
7.85 7.85 7.85 7.84 7.84 7.83 7.58 7.58 7.57 7.56 7.56 7.56 7.55 7.55 7.33 7.33 7.33 7.32 7.32 7.32 7.32 7.27 7.25 7.25 7.24 7.24 7.23 7.23 7.23 7.23 7.22 7.22 7.21 7.21 7.21 7.21 7.19 7.18 7.18 7.18 7.17 7.17 7.16 7.14 7.14 7.14 7.14 7.13 7.13 7.13 7.13 7.12 7.11 7.05 7.04 7.04 7.03 7.02 7.02 7.01 6.96 6.96 6.96 6.96 6.95 6.95 6.95 6.94 6.94
N
N
O
F
1.00
1.05
1.02
2.11
1.96
1.05
3.13
1.09
1.04
f1 (ppm)

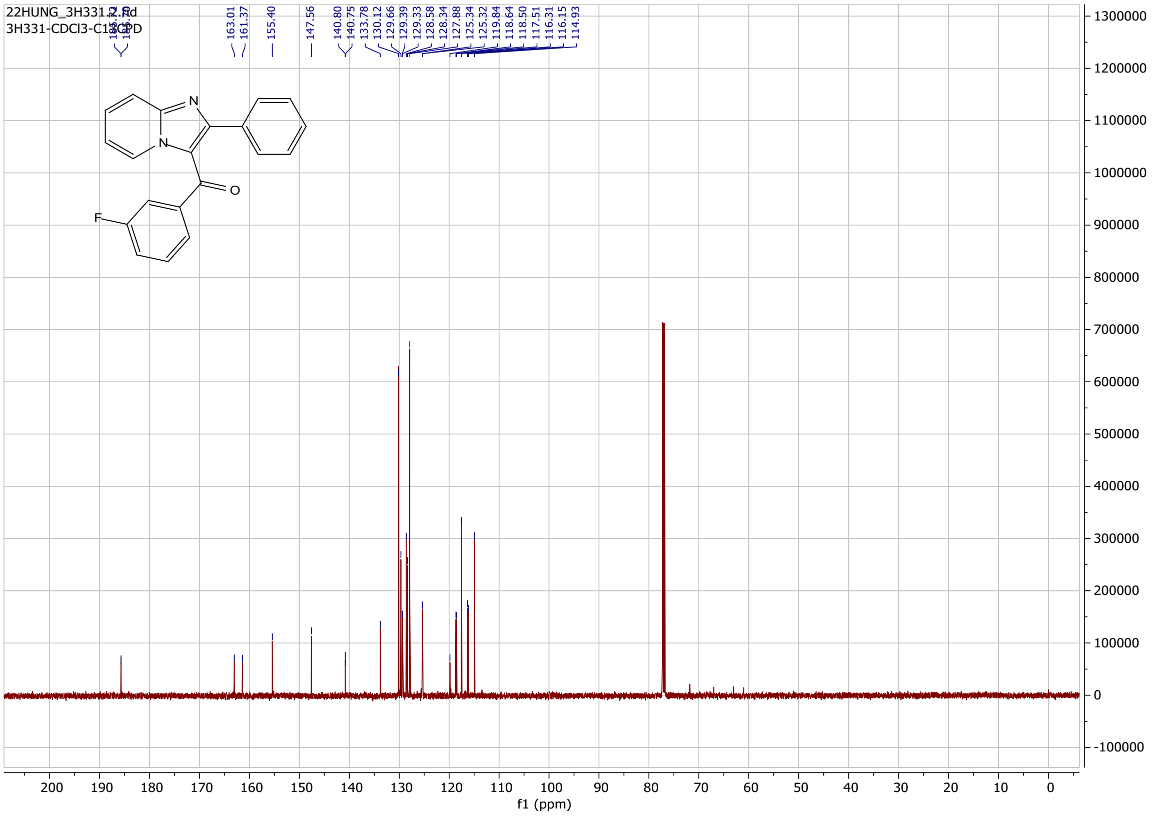
22HUNG_3H331.2.fid
3H331-CDCl3-C13CPD
163.01
161.37
155.40
147.56
140.80
140.75
133.78
130.12
129.66
129.39
129.33
128.58
128.34
127.88
125.34
125.32
119.84
118.64
118.50
117.51
116.31
116.15
114.93
N
N
O
F
f1 (ppm)

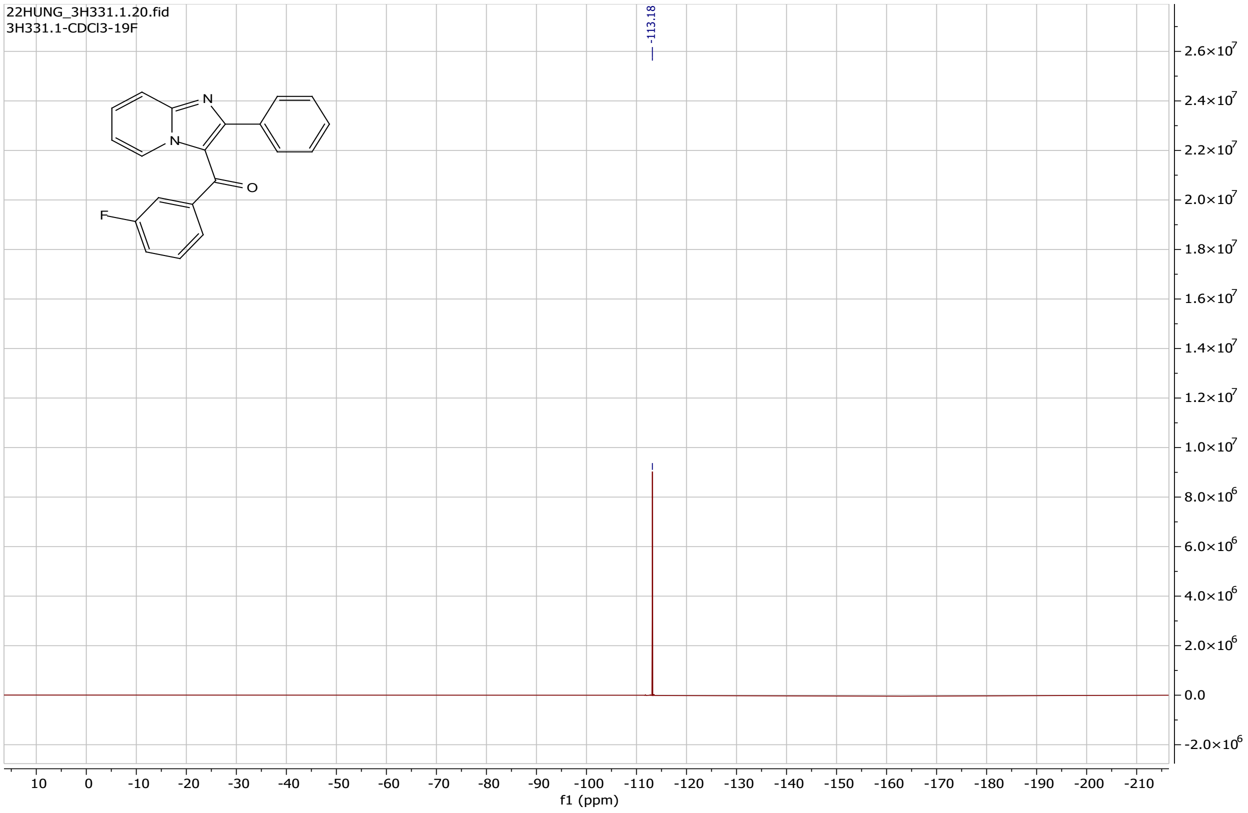

(6-methyl-2-phenylimidazo[1,2-a]pyridin-3-yl)(pyridin-2-yl)methanone 3o

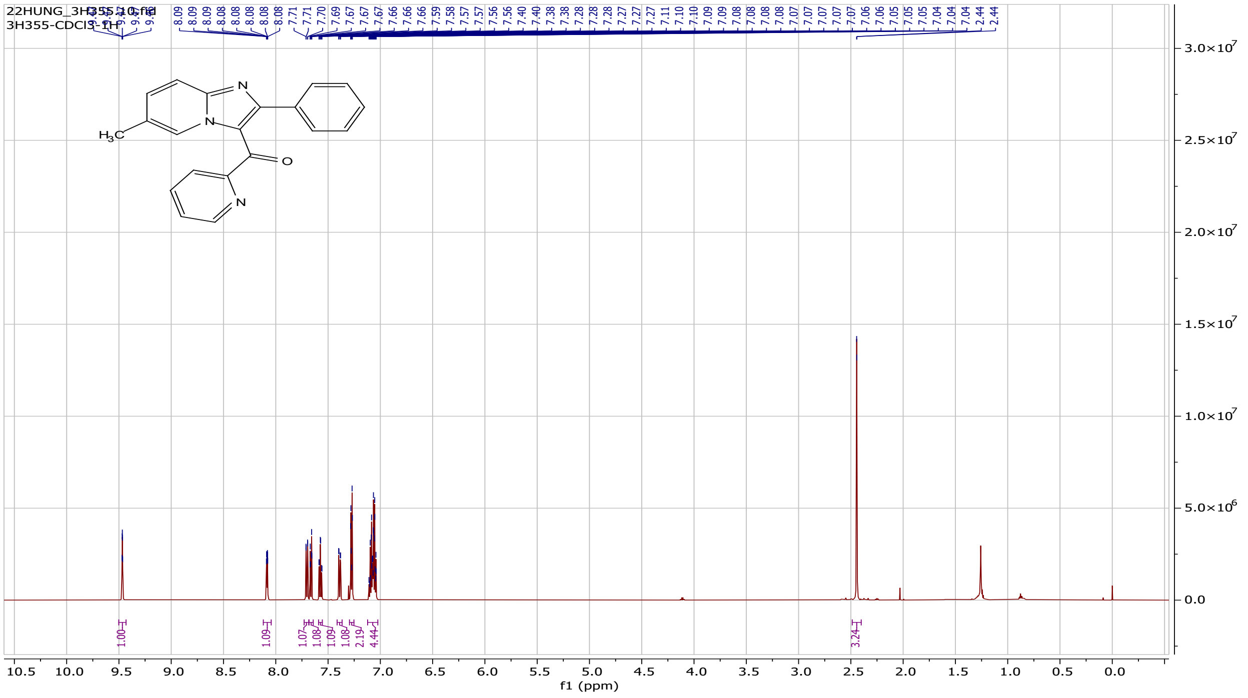

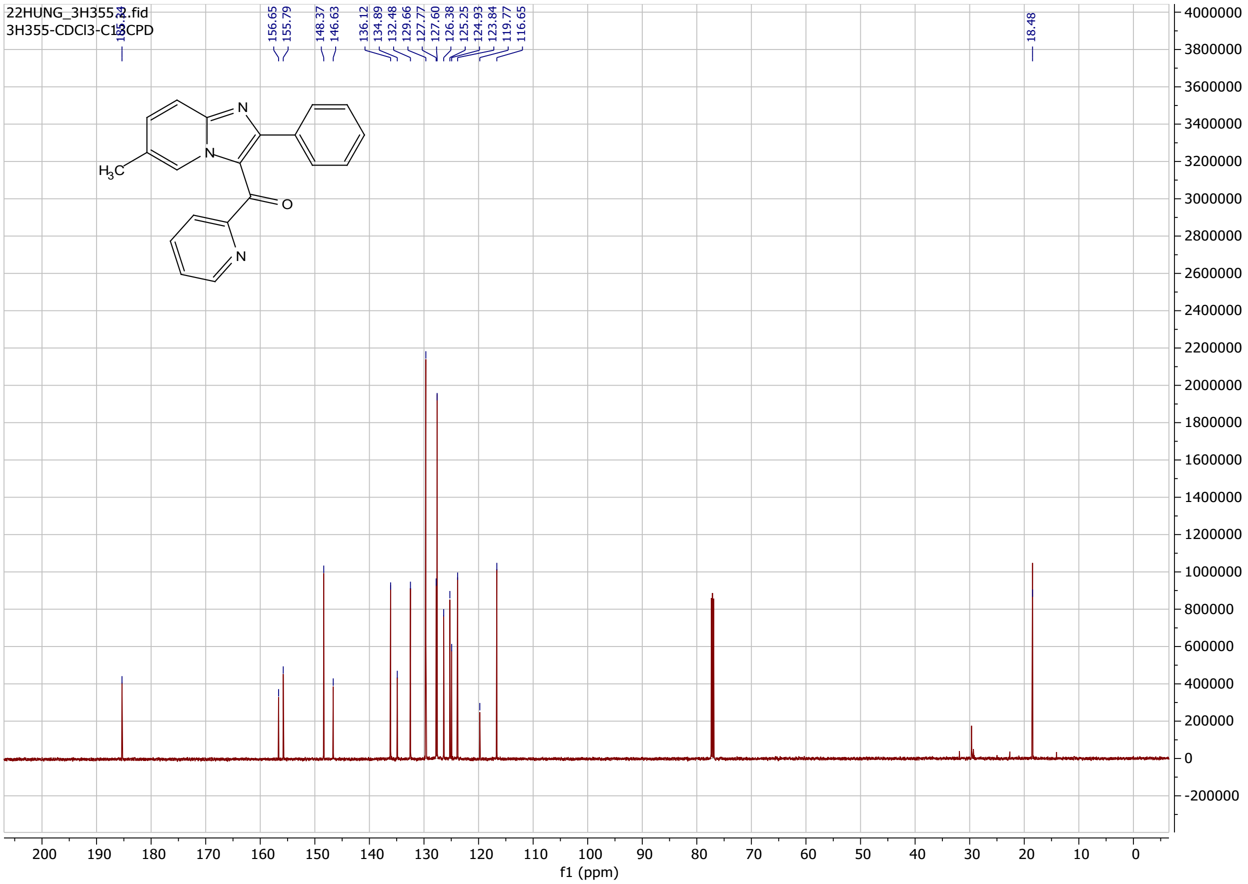

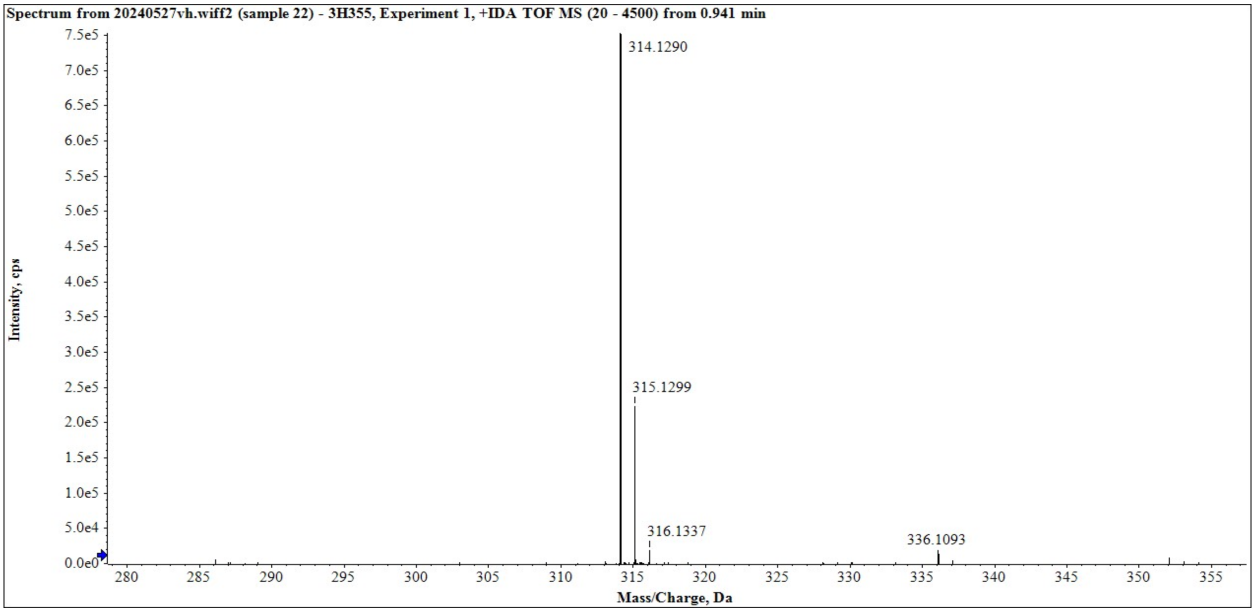

(7-methyl-2-phenylimidazo[1,2-a]pyridin-3-yl)(pyridin-2-yl)methanone 3p

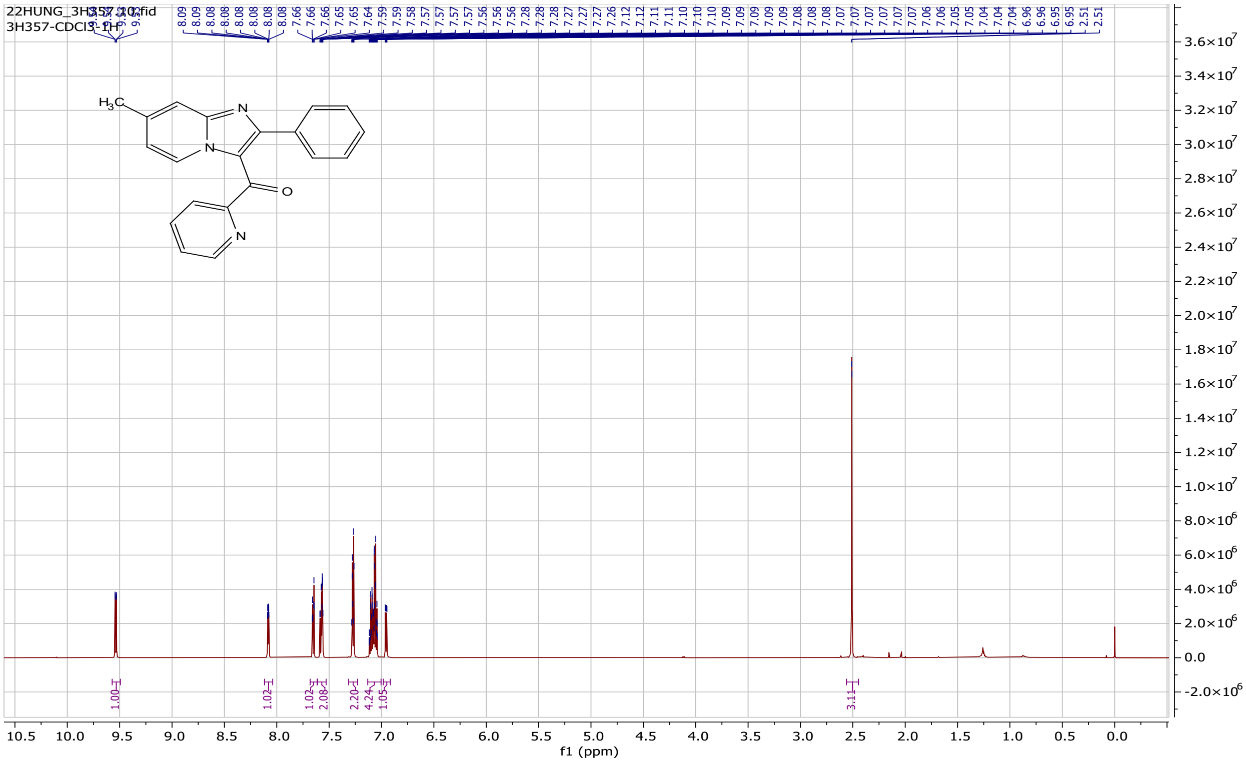
22HUNG_3H357.10.fid
3H357-CDCl3-1H
H_3C
N
O
f1 (ppm)

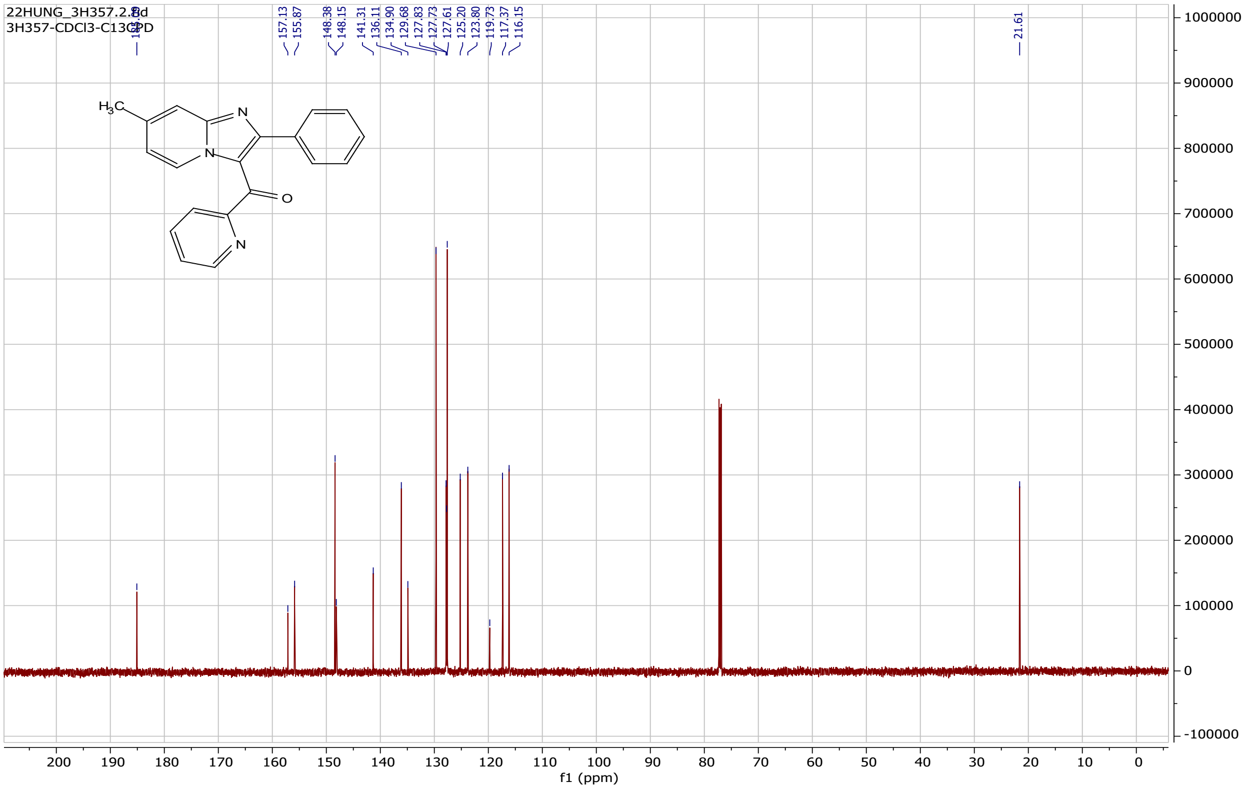
3H357-CDCl3-C13CPD
157.13
155.87
148.38
148.15
141.31
136.11
134.90
129.68
127.83
127.73
127.61
125.20
123.80
119.73
117.37
116.15
21.61
H_3C
N
O
f1 (ppm)

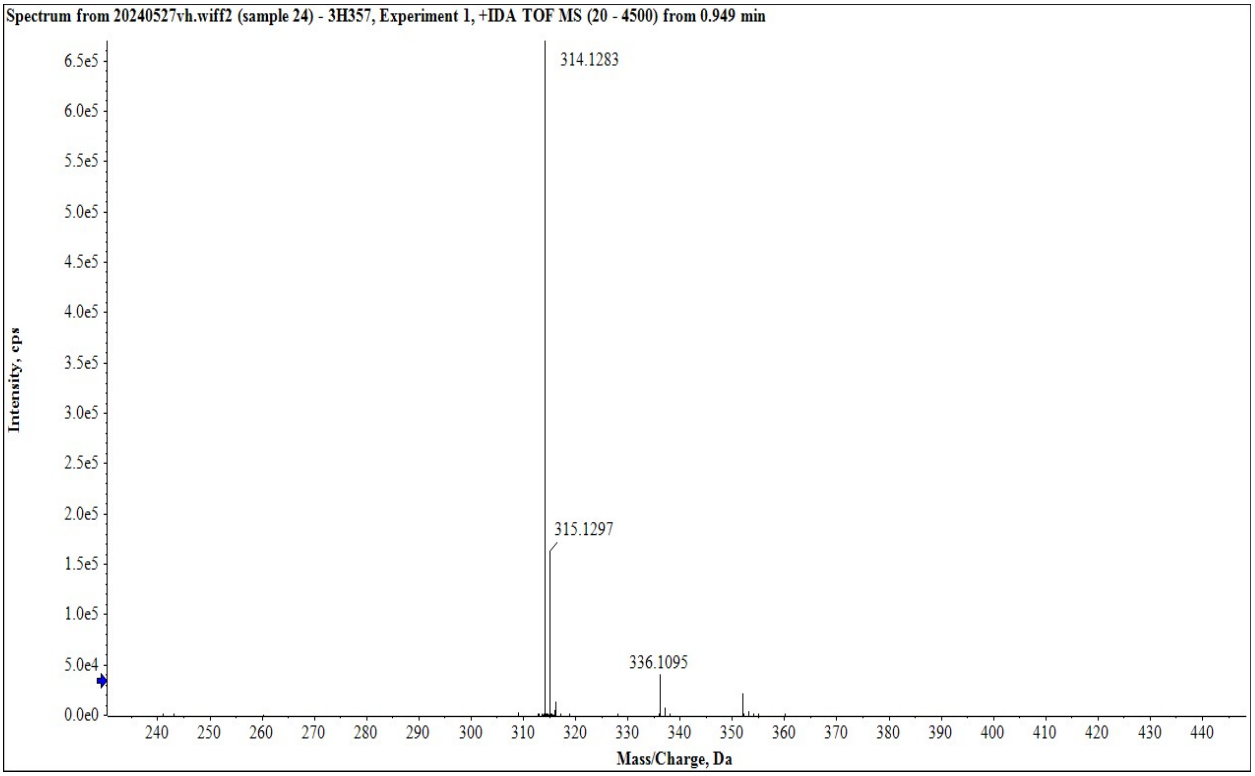

(6-methyl-2-(p-tolyl)imidazo[1,2-a]pyridin-3-yl)(pyridin-2-yl)methanone 3q

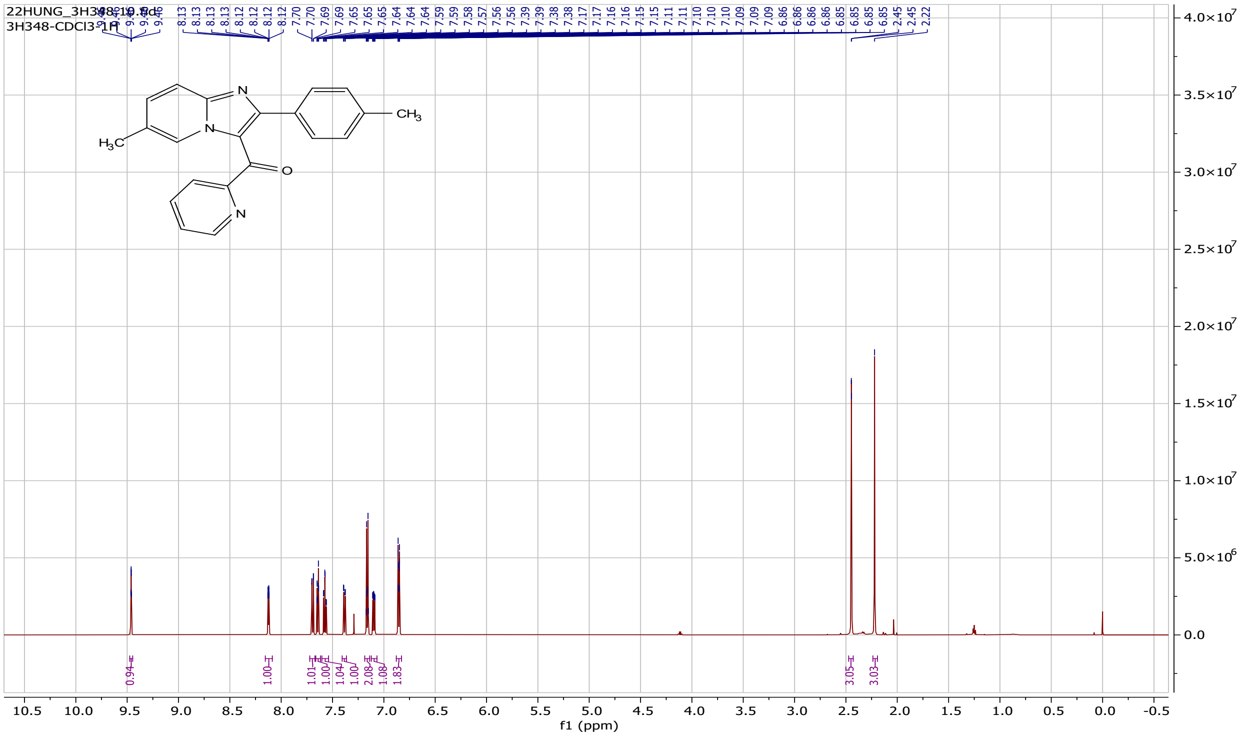

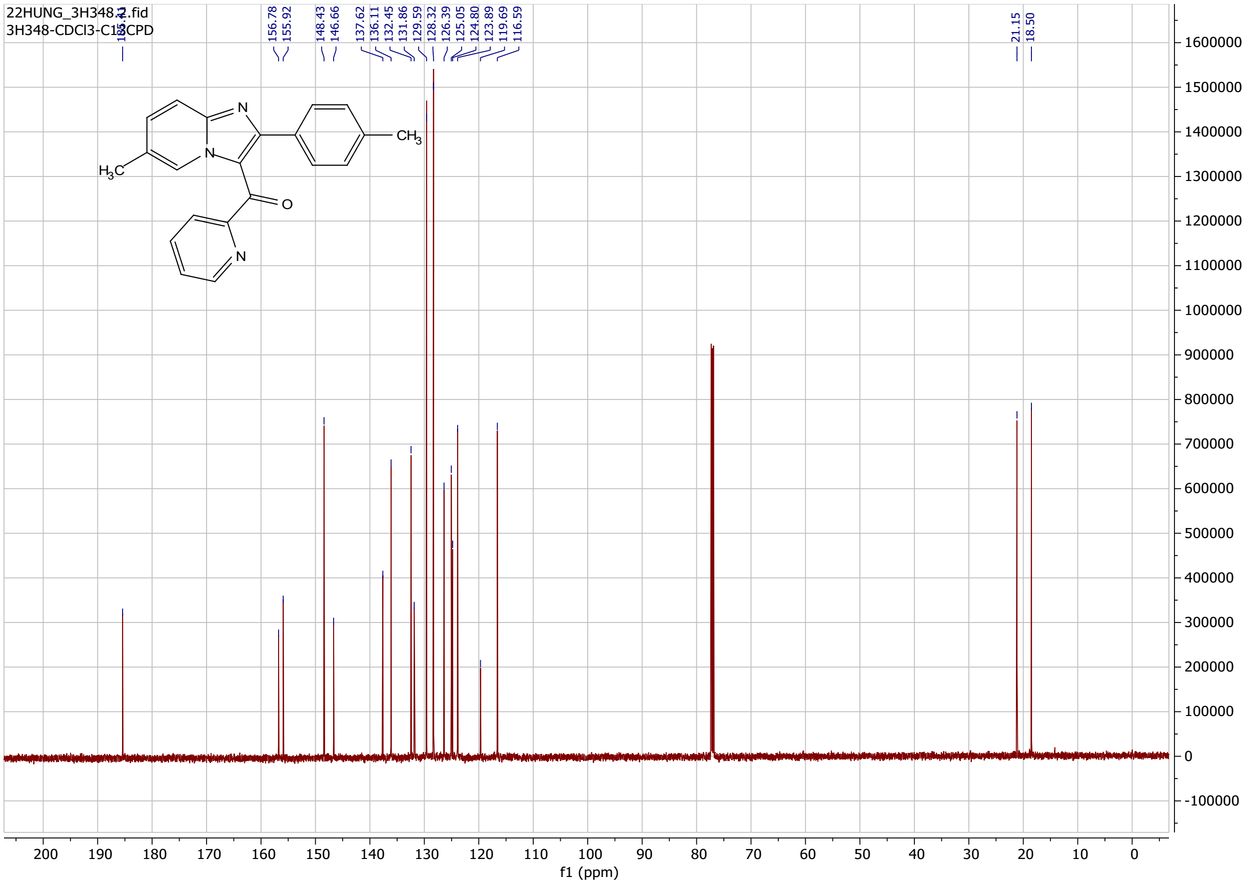
22HUNG_3H348.2.fid
3H348-CDCl3-C13CPD
156.78
155.92
148.43
146.66
137.62
136.11
132.45
131.86
129.59
128.32
126.39
125.05
124.80
123.89
119.69
116.59
21.15
18.50
f1 (ppm)

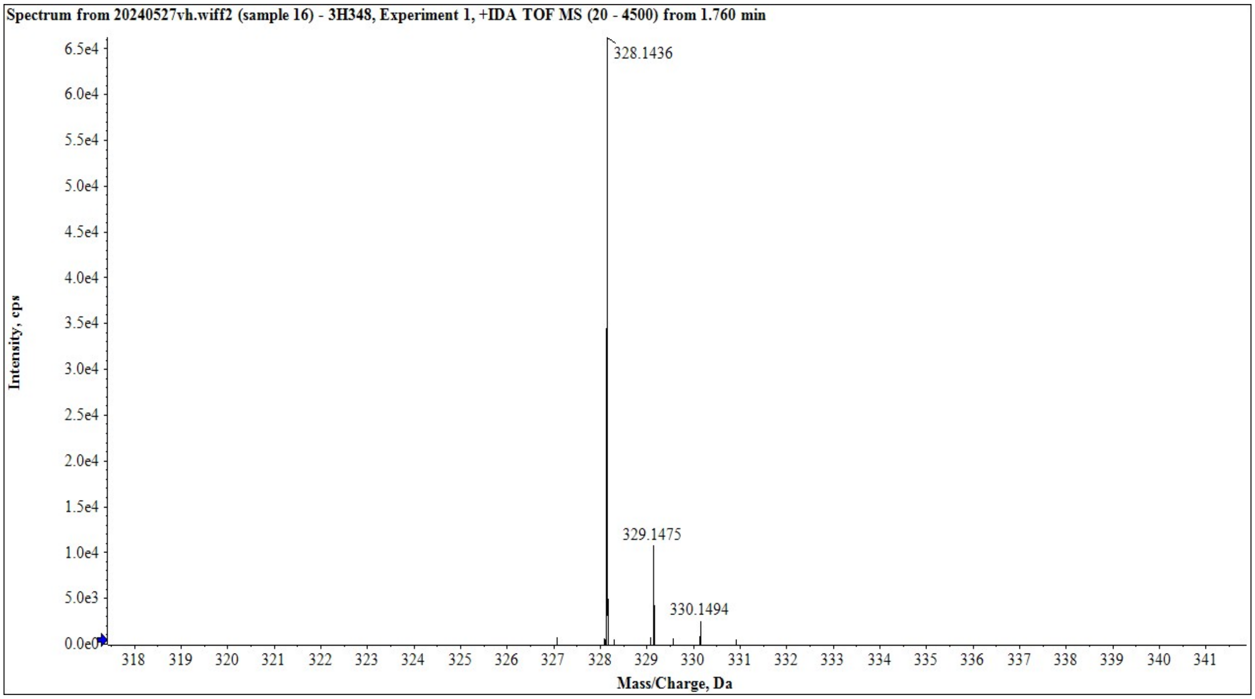
Spectrum from 20240527vh.wiff2 (sample 16) - 3H348, Experiment 1, +IDA TOF MS (20 - 4500) from 1.760 min
Intensity, cps
328.1436
329.1475
330.1494
Mass/Charge, Da

(7-chloro-2-phenylimidazo[1,2-a]pyridin-3-yl)(pyridin-2-yl)methanone 3r

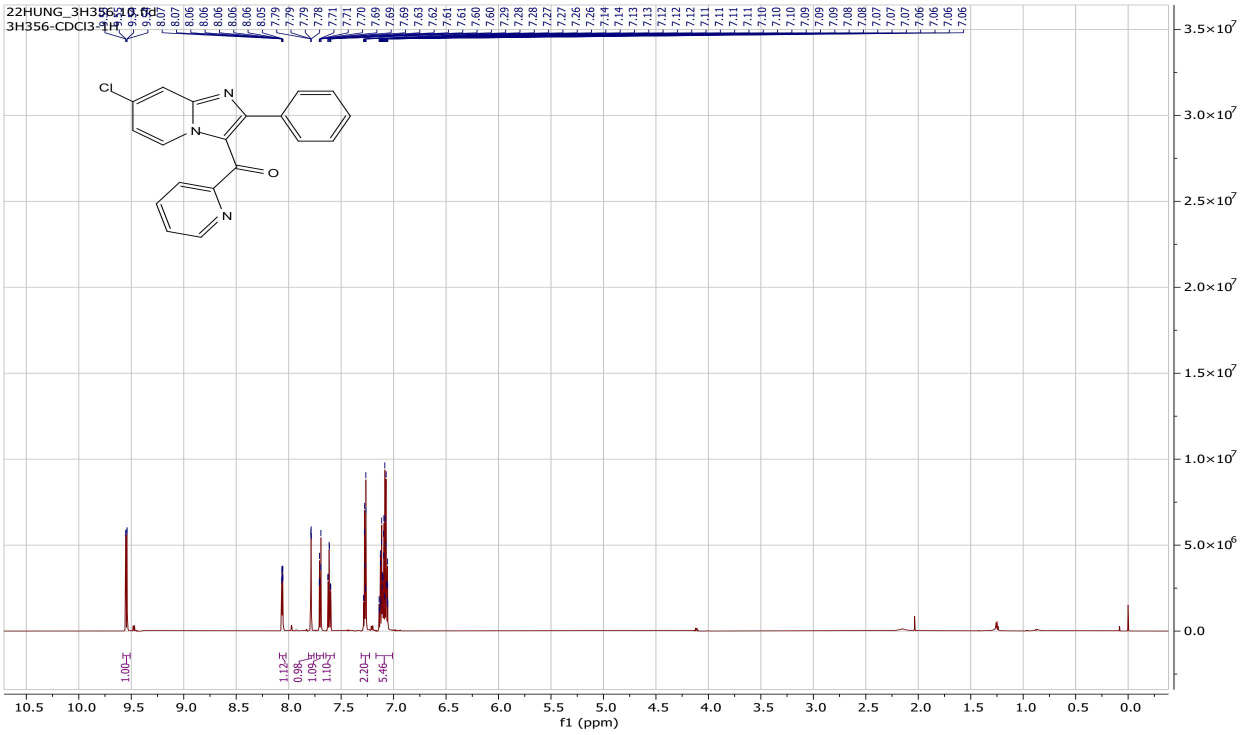

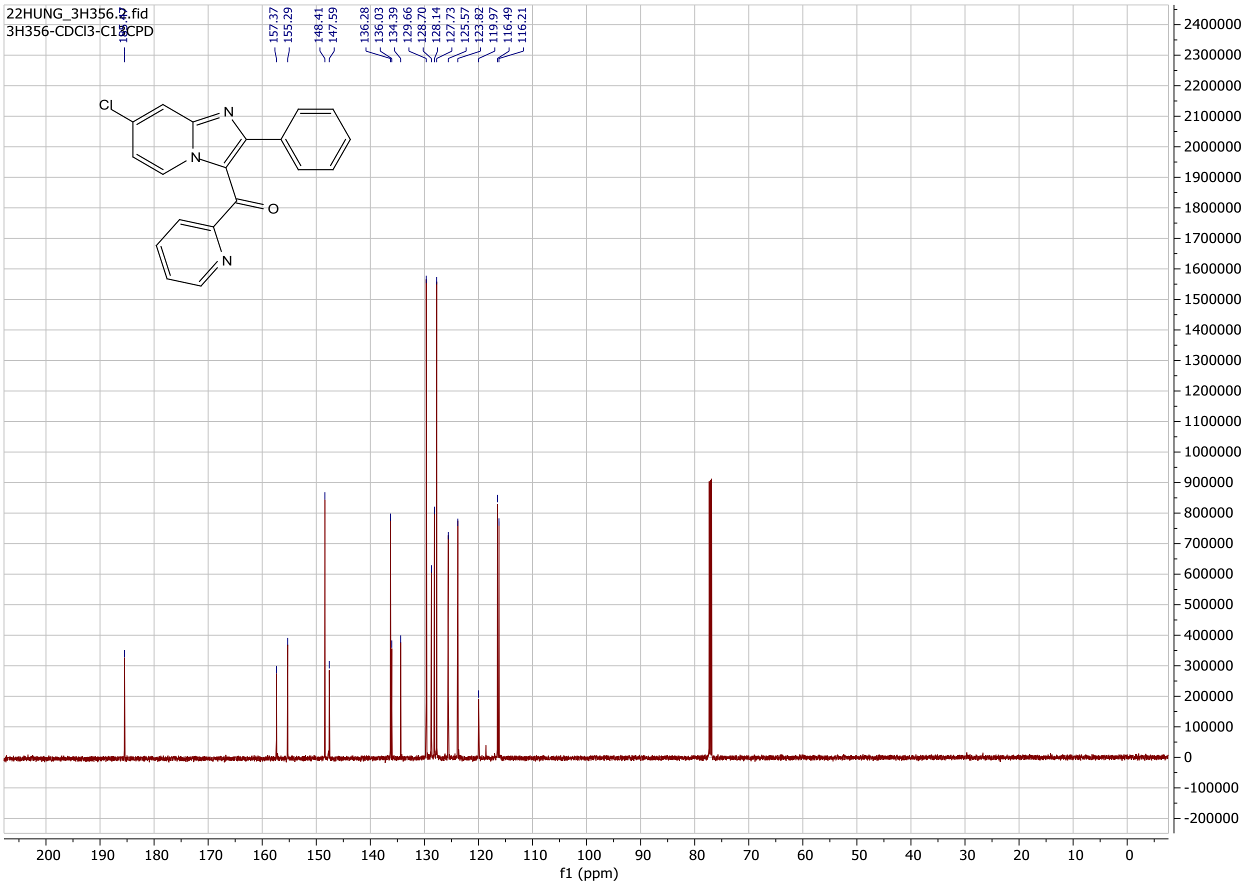

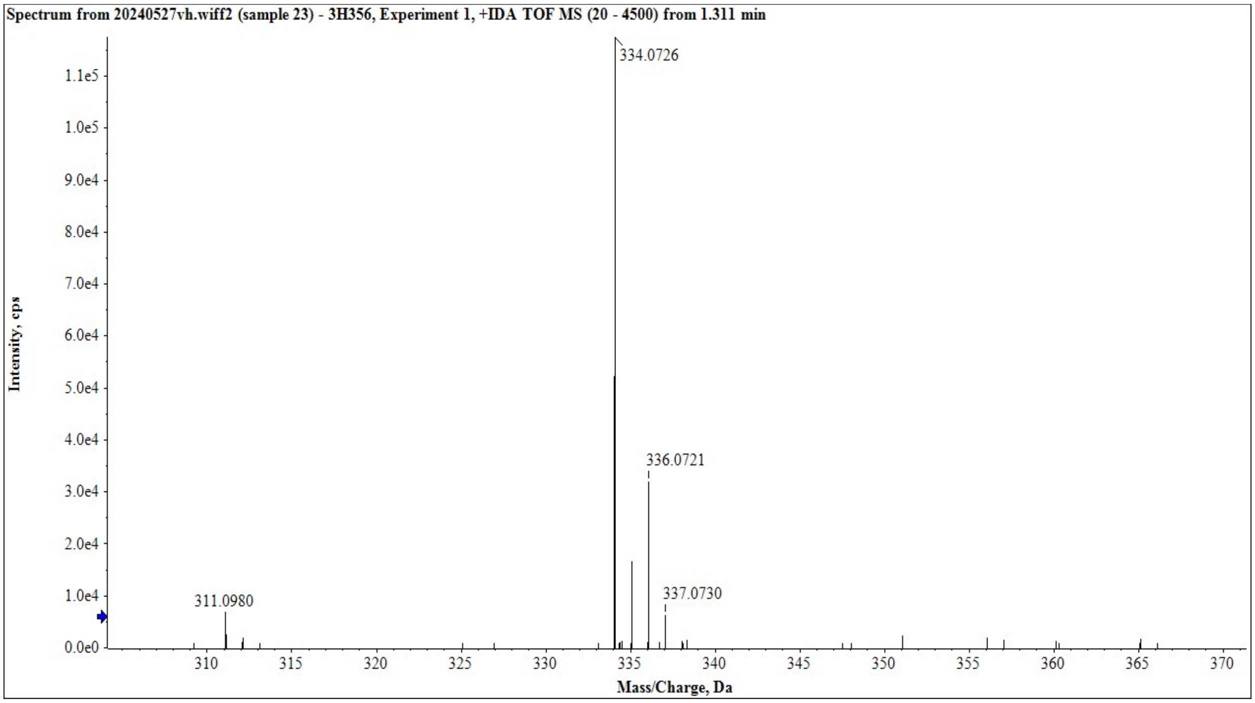

3,3'-(phenylmethylene)bis(2-phenylimidazo[1,2-a]pyridine 4a

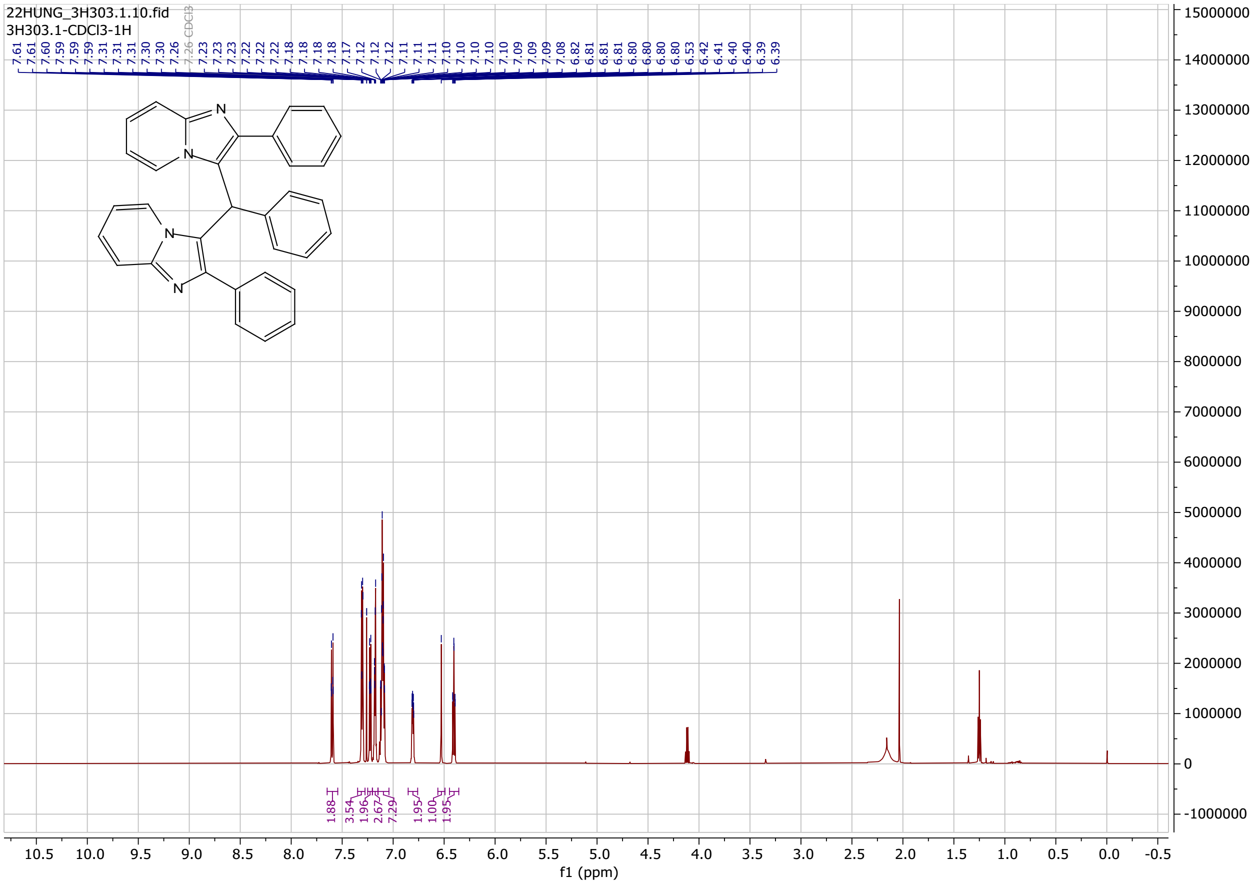

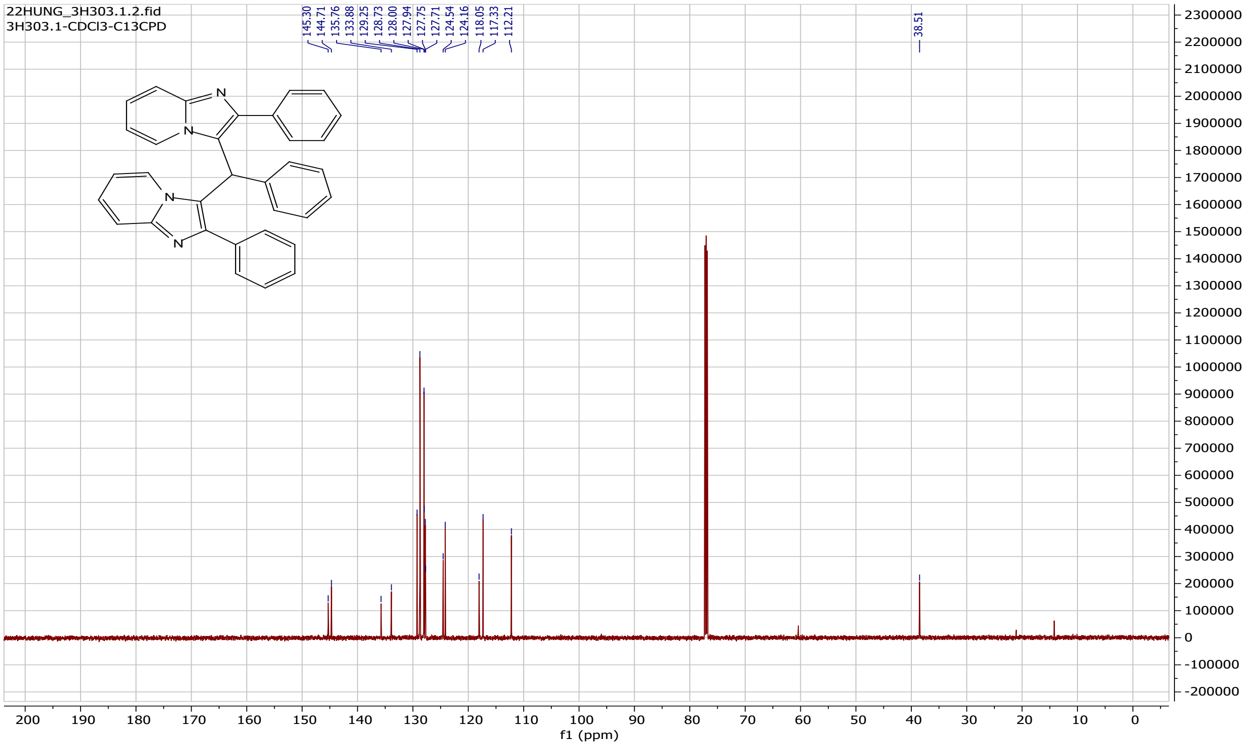

3,3'-(p-tolylmethylene)bis(2-phenylimidazo[1,2-a]pyridine) 4b

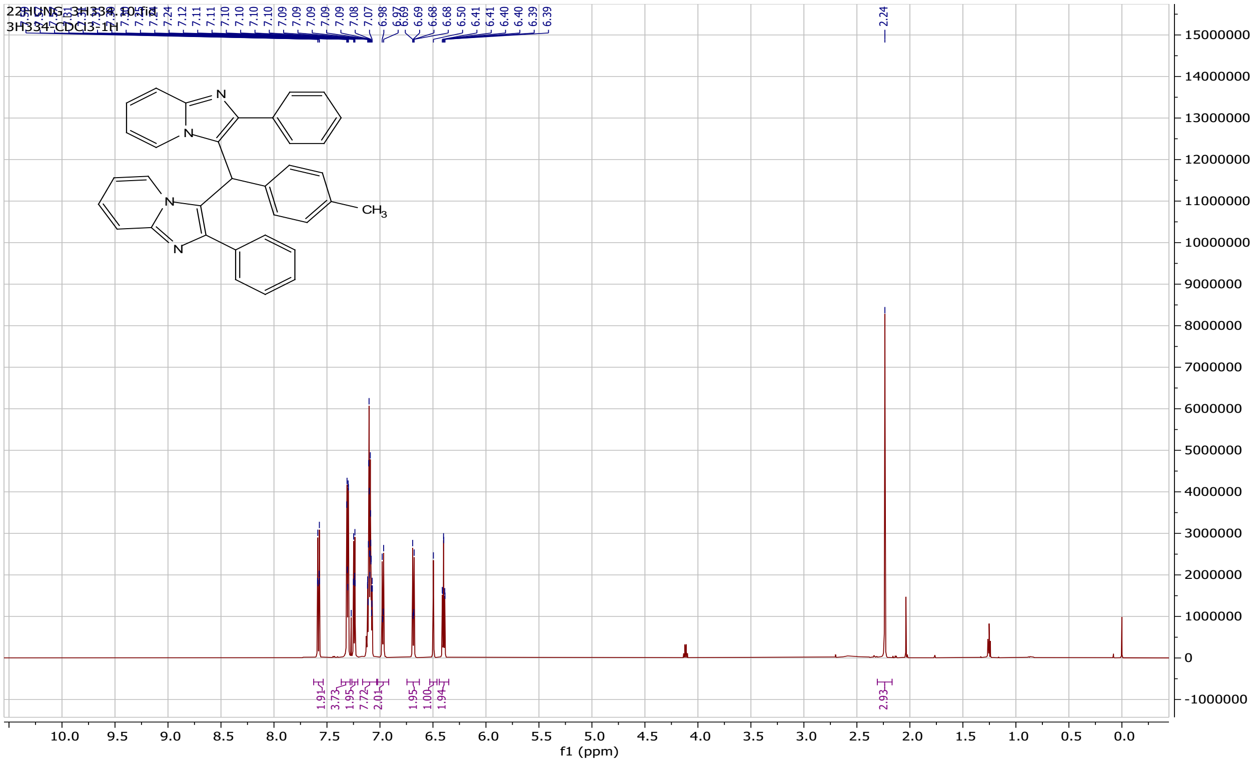
3H334-CDCl3-1H
2.24
1.91
3.73
1.95
7.72
2.01
1.95
1.00
1.94
2.93
f1 (ppm)

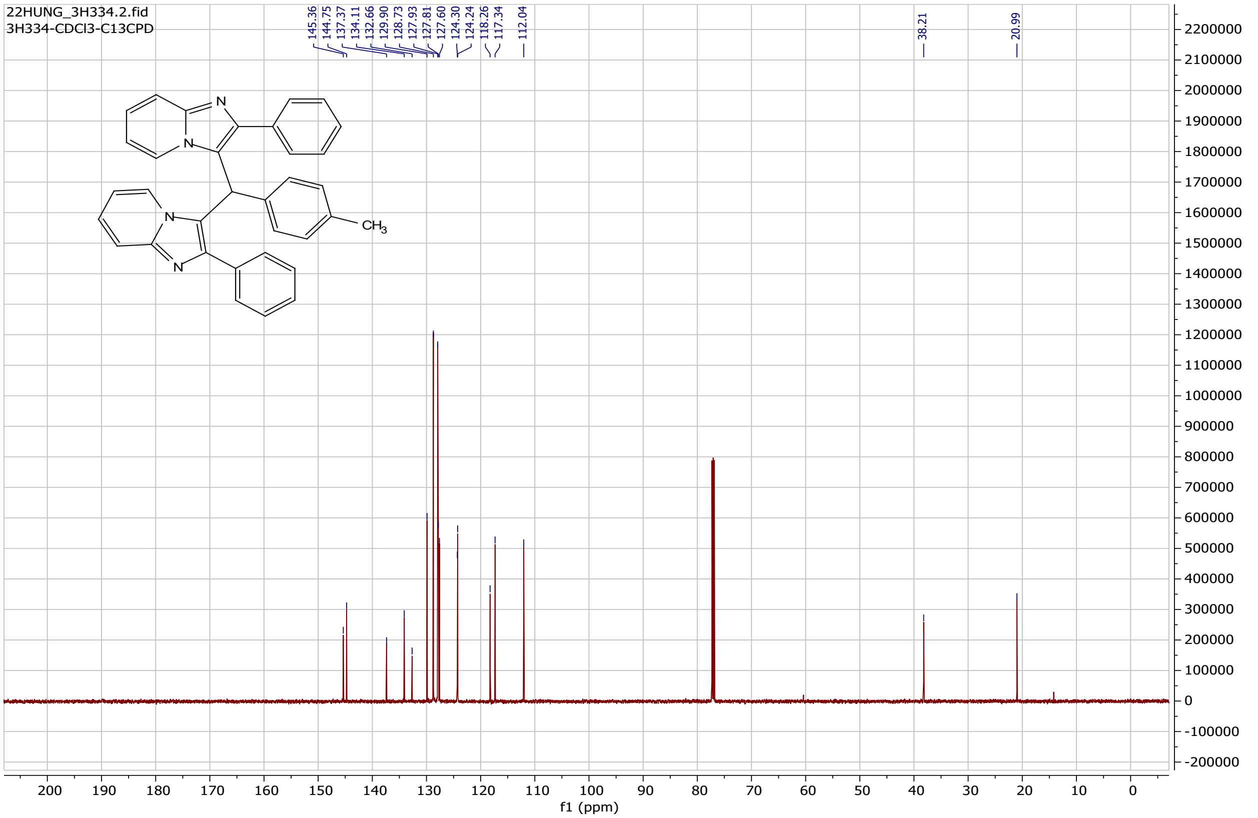
22HUNG_3H334.2.fid
3H334-CDCl3-C13CPD
145.36
144.75
137.37
134.11
132.66
129.90
128.73
127.93
127.81
127.60
124.30
124.24
118.26
117.34
112.04
38.21
20.99
CH3
f1 (ppm)

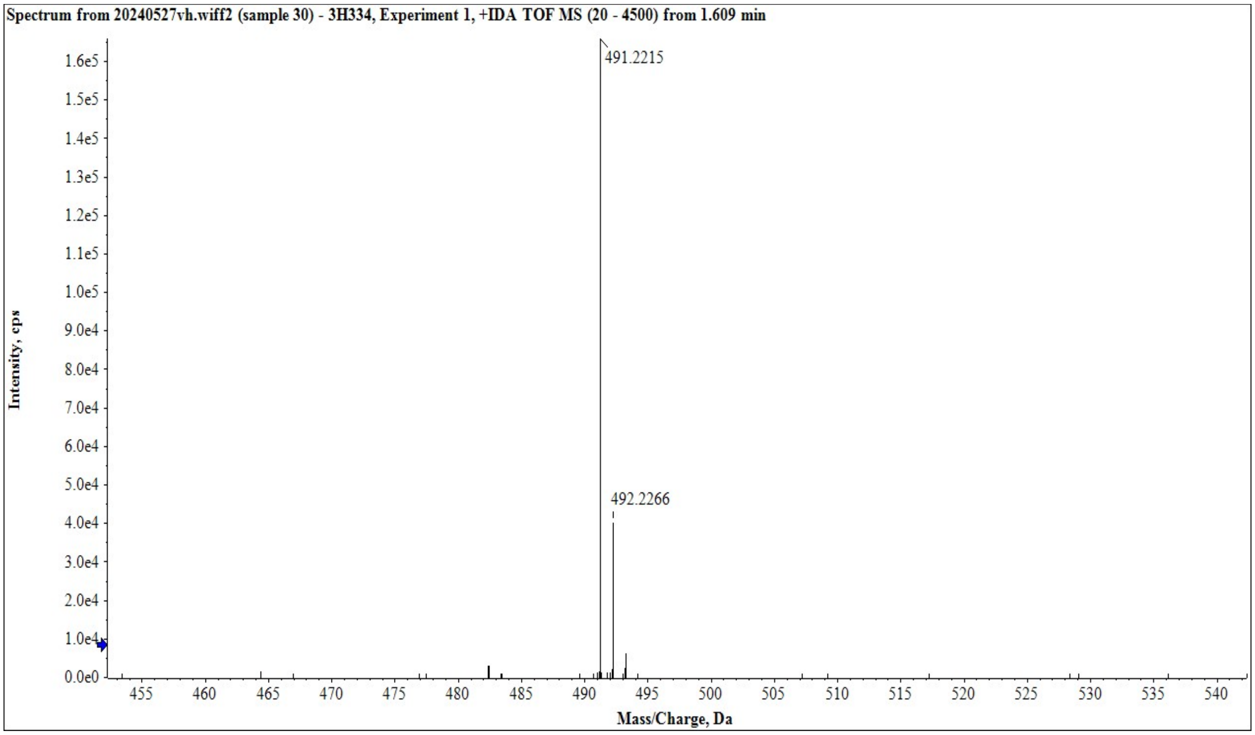

3,3'-((4-methoxyphenyl)methylene)bis(2-phenylimidazo[1,2-a]pyridine 4c

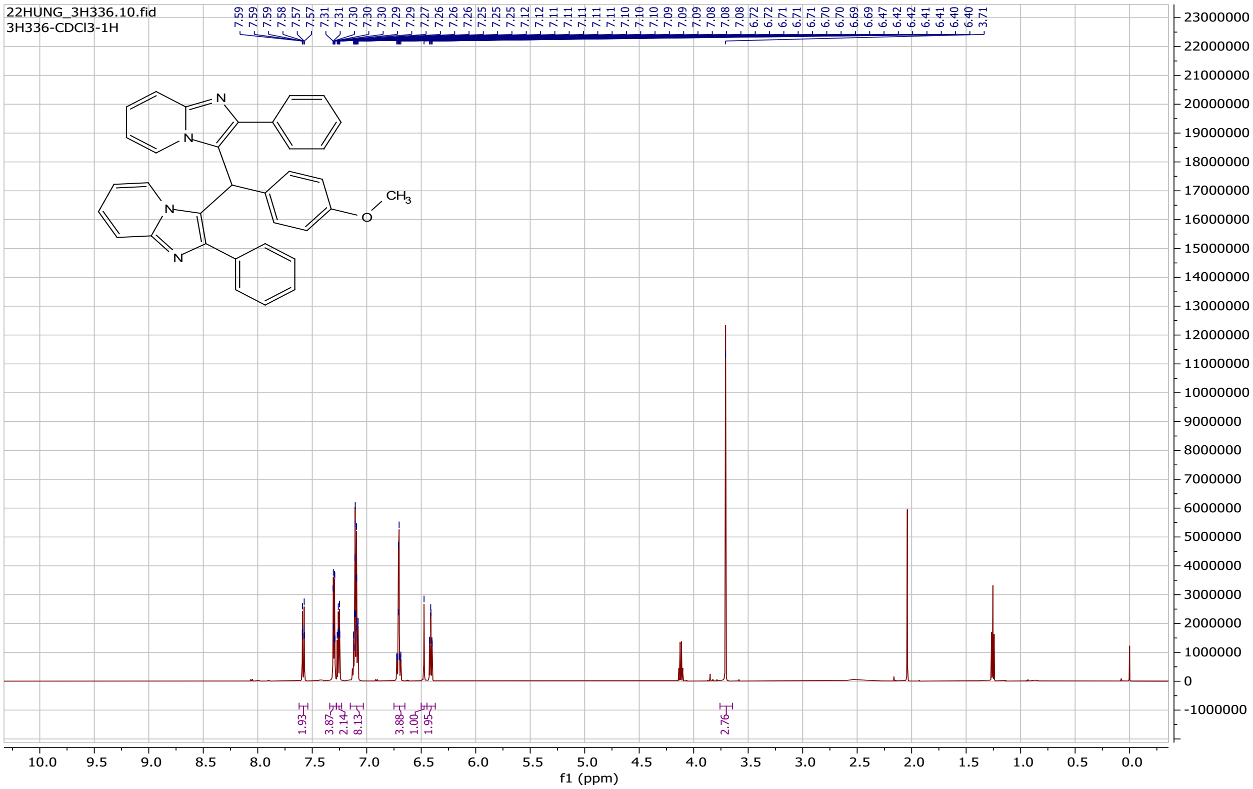

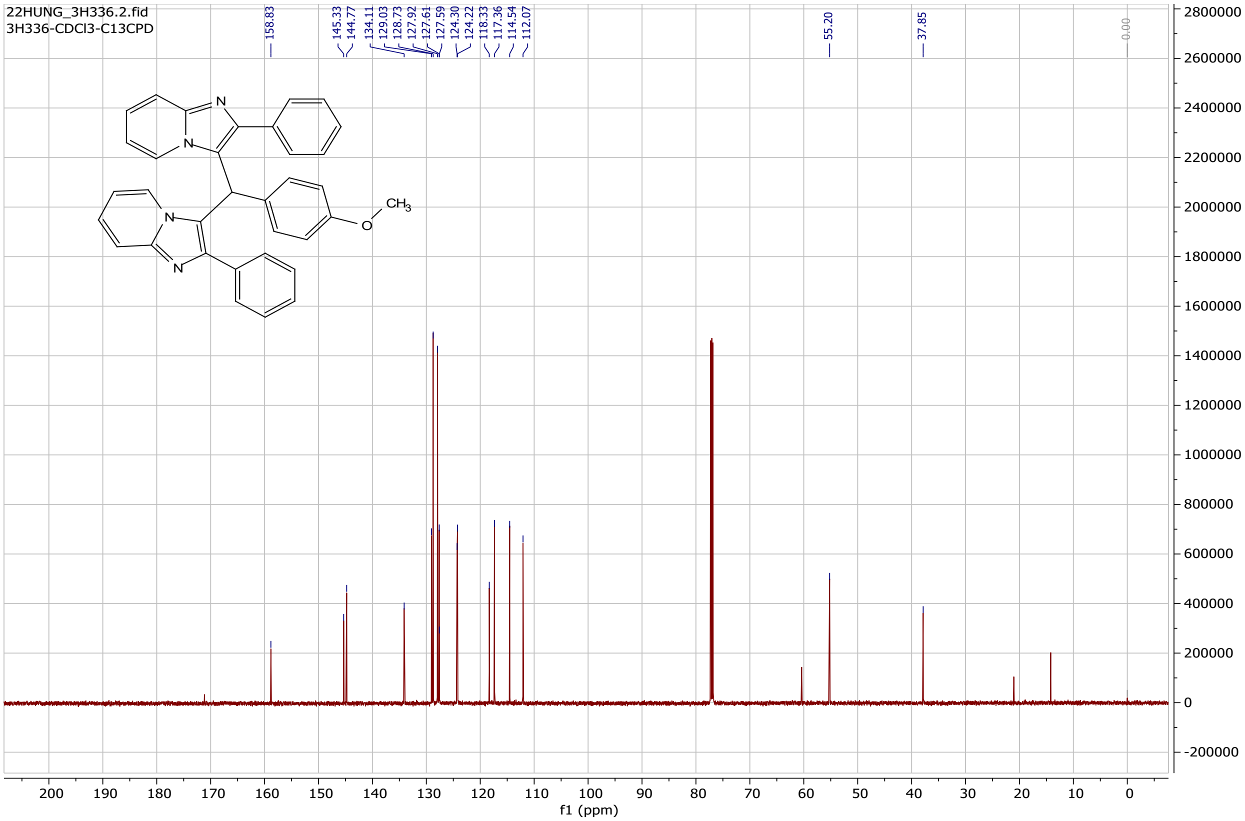
22HUNG_3H336.2.fid
3H336-CDCl3-C13CPD
158.83
145.33
144.77
134.11
129.03
128.73
127.92
127.61
127.59
124.30
124.22
118.33
117.36
114.54
112.07
55.20
37.85
0.00
N
N
N
N
O
CH3
f1 (ppm)

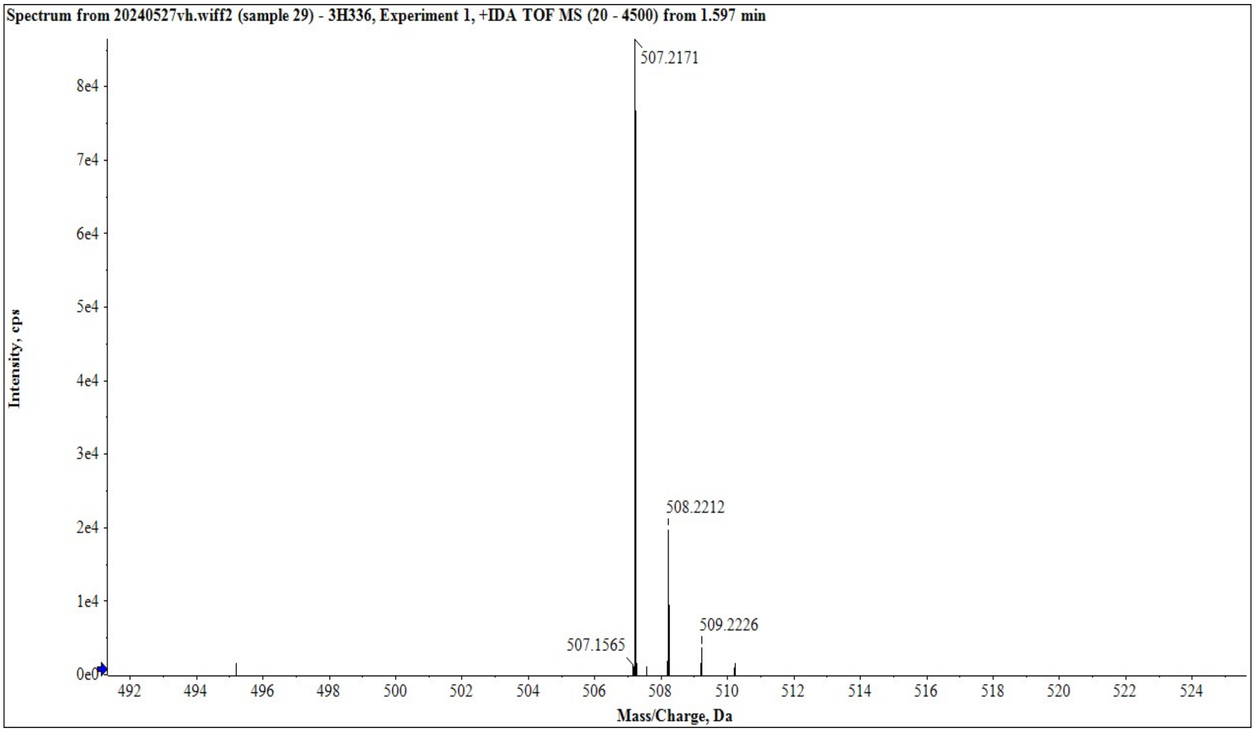
Spectrum from 20240527vh.wiff2 (sample 29) - 3H336, Experiment 1, +IDA TOF MS (20 - 4500) from 1.597 min
507.2171
508.2212
509.2226
507.1565
Intensity, cps
Mass/Charge, Da

3,3'-((4-chlorophenyl)methylene)bis(2-phenylimidazo[1,2-a]pyridine) 4d

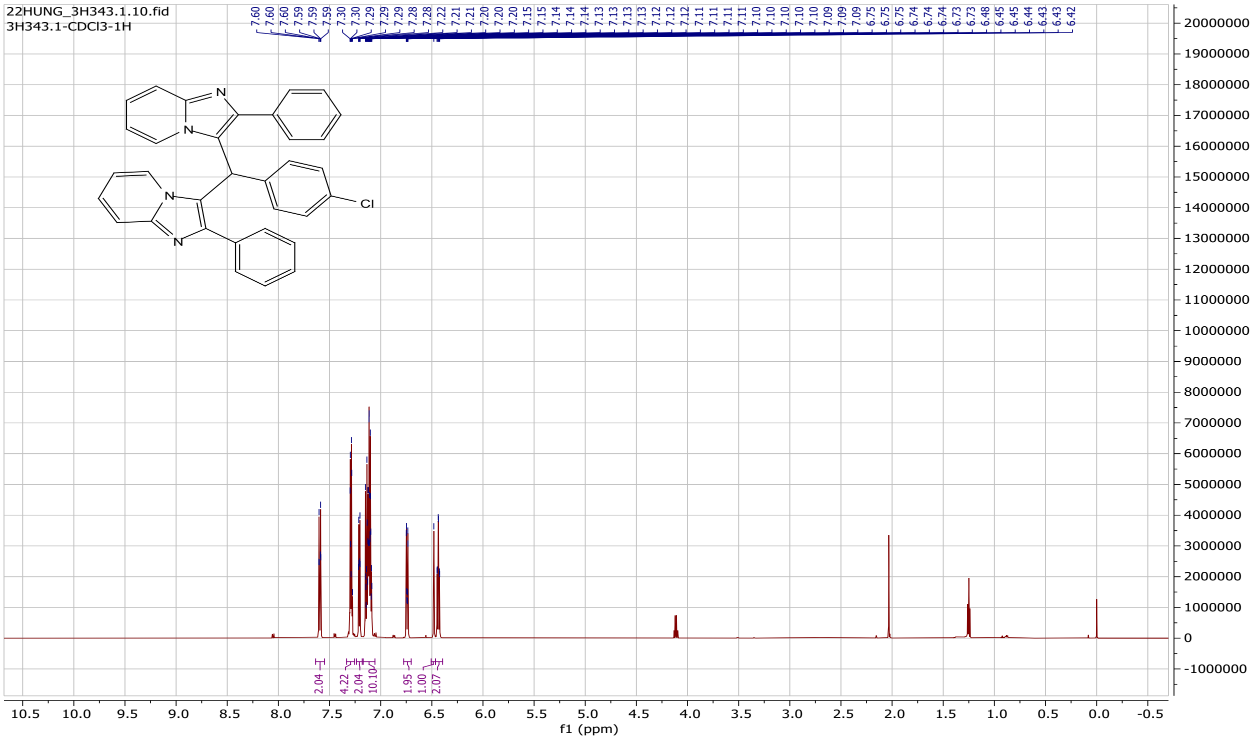

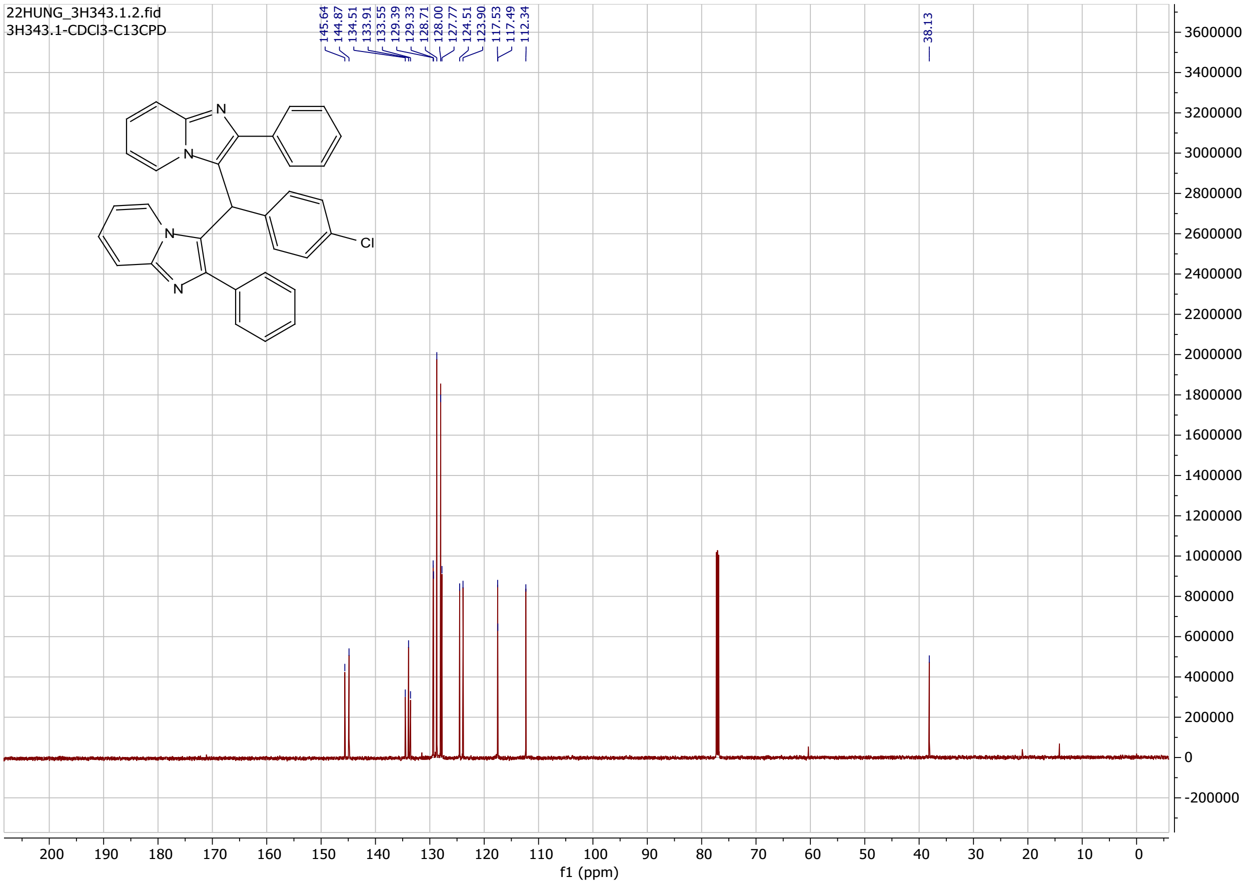

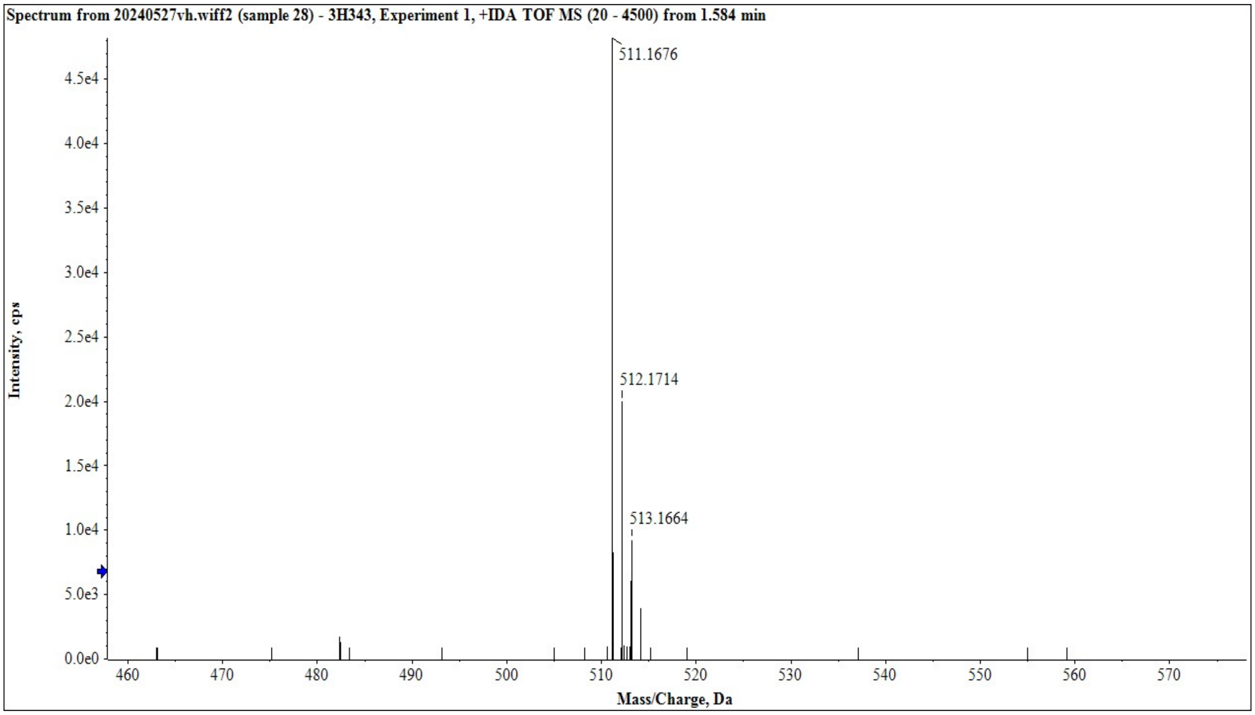

3,3'-((3-fluorophenyl)methylene)bis(2-phenylimidazo[1,2-a]pyridine 4e

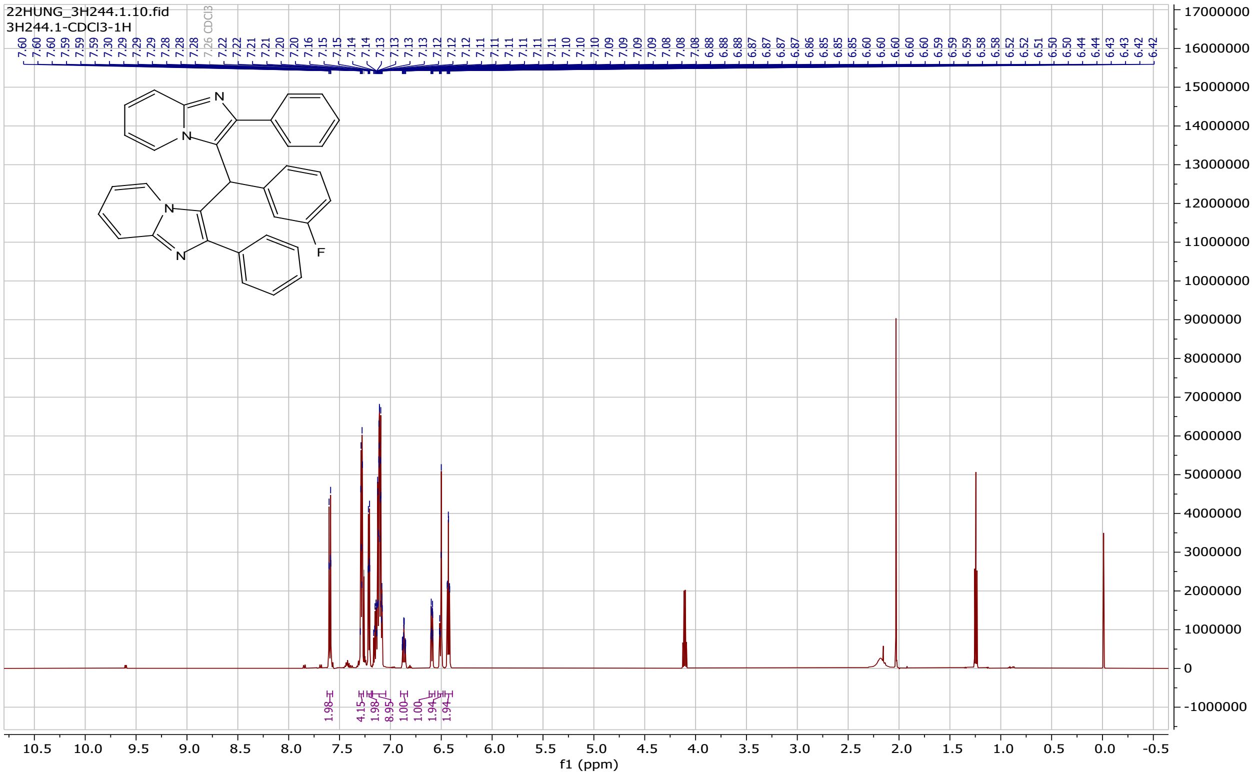

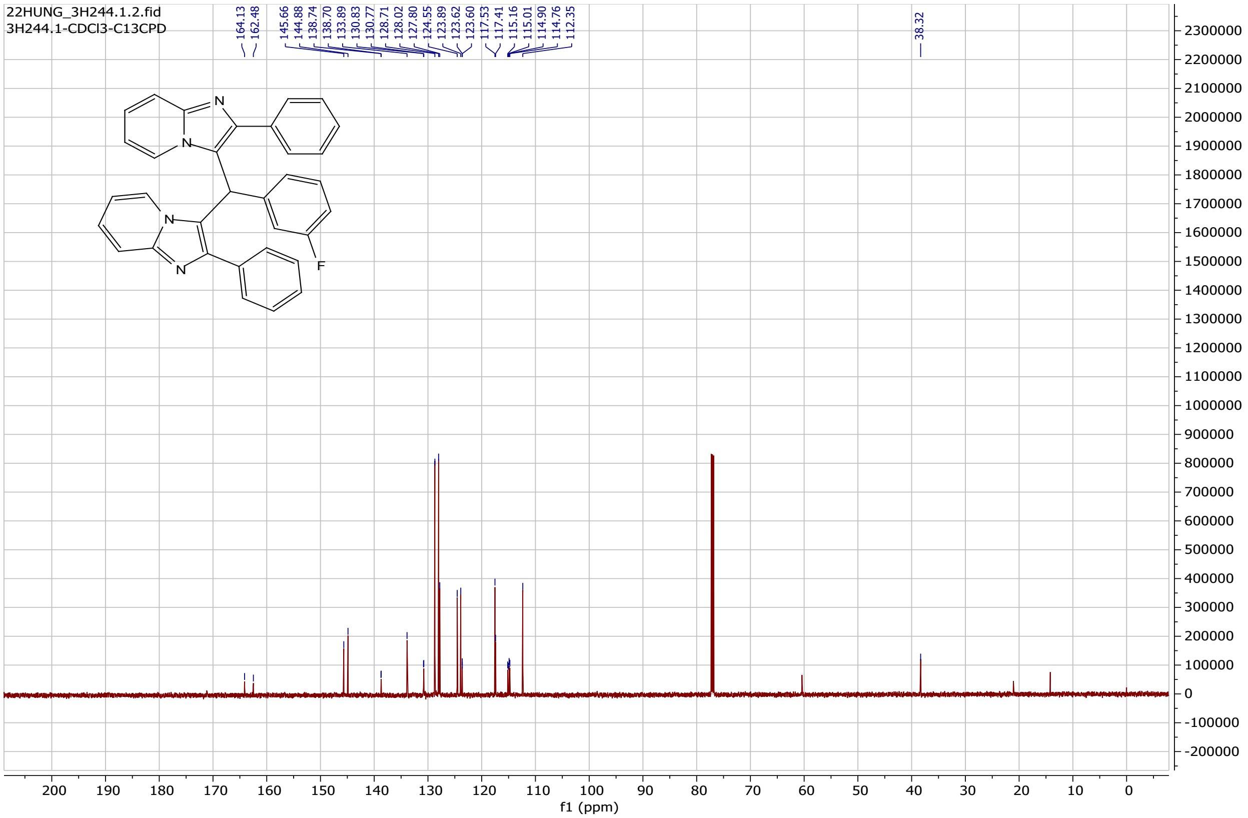
22HUNG_3H244.1.2.fid
3H244.1-CDCl3-C13CPD
164.13
162.48
145.66
144.88
138.74
138.70
133.89
130.83
130.77
128.71
128.02
127.80
124.55
123.89
123.62
123.60
117.53
117.41
115.16
115.01
114.90
114.76
112.35
38.32
N
N
N
N
F
f1 (ppm)

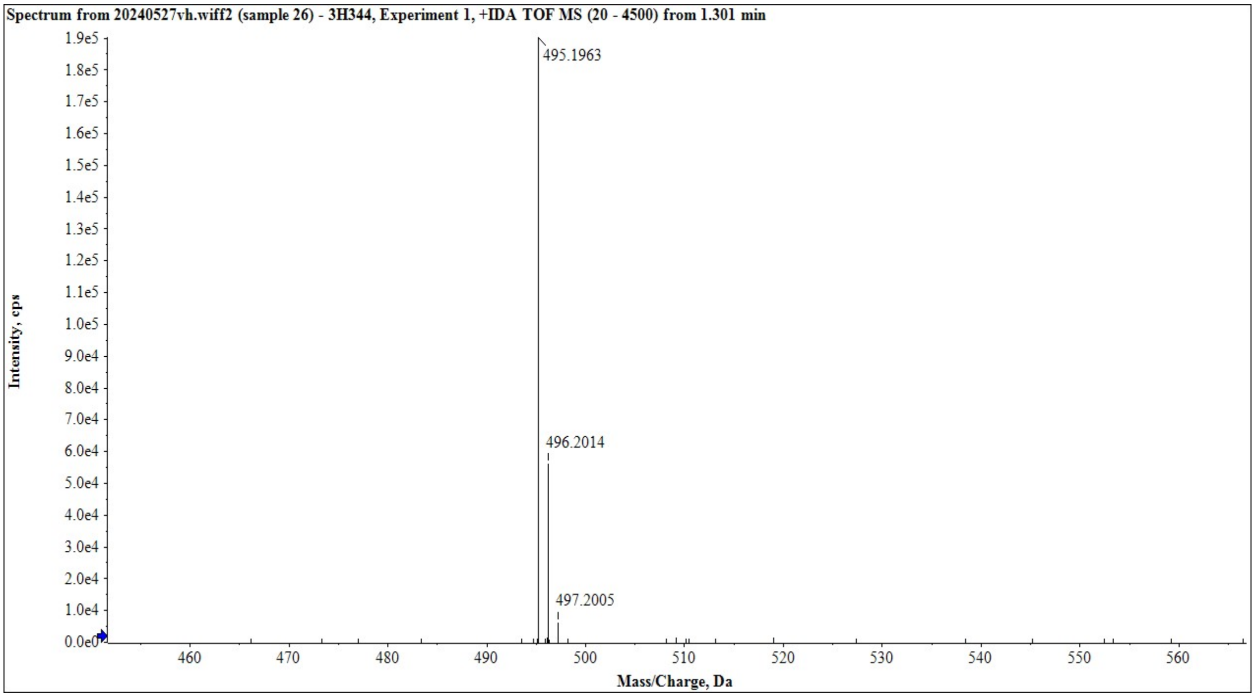
Spectrum from 20240527vh.wiff2 (sample 26) - 3H344, Experiment 1, +IDA TOF MS (20 - 4500) from 1.301 min
495.1963
496.2014
497.2005
Intensity, cps
Mass/Charge, Da

3,3'-(benzo[d][1,3]dioxol-5-ylmethylene)bis(2-phenylimidazo[1,2-a]pyridine 4f

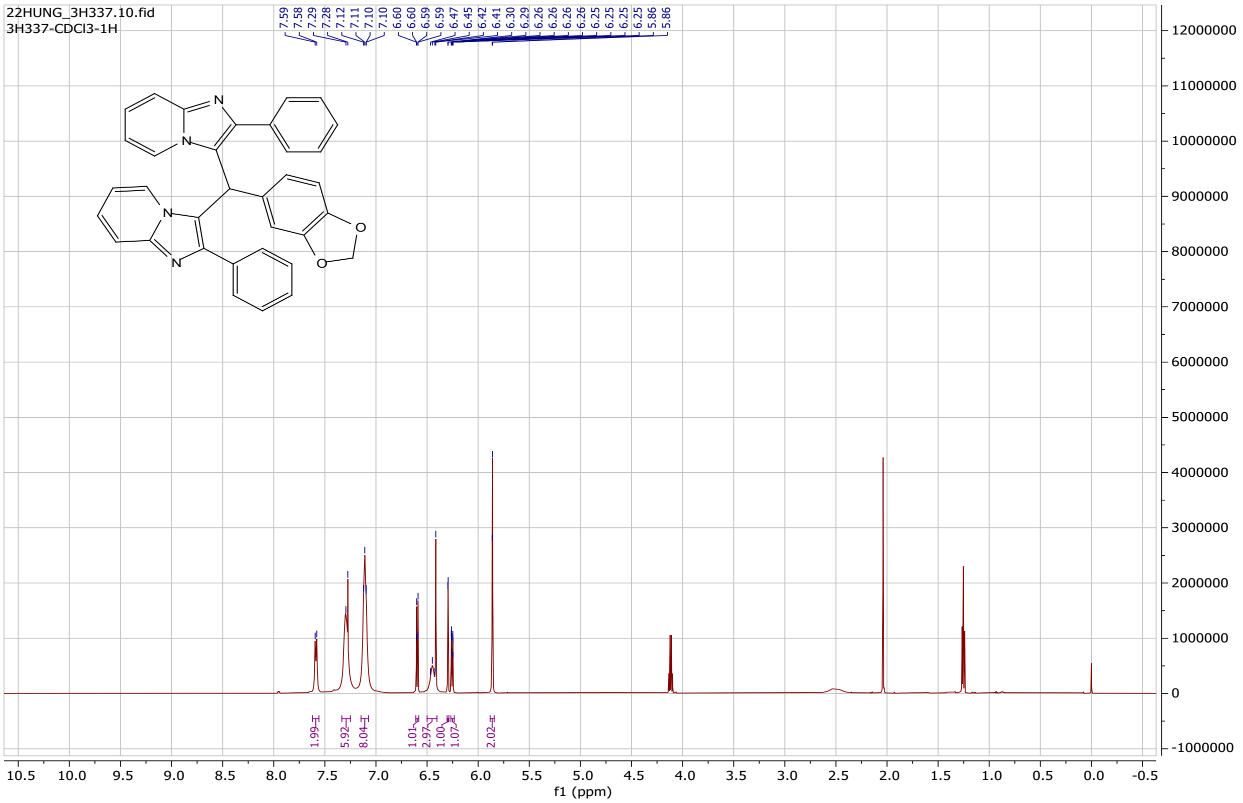

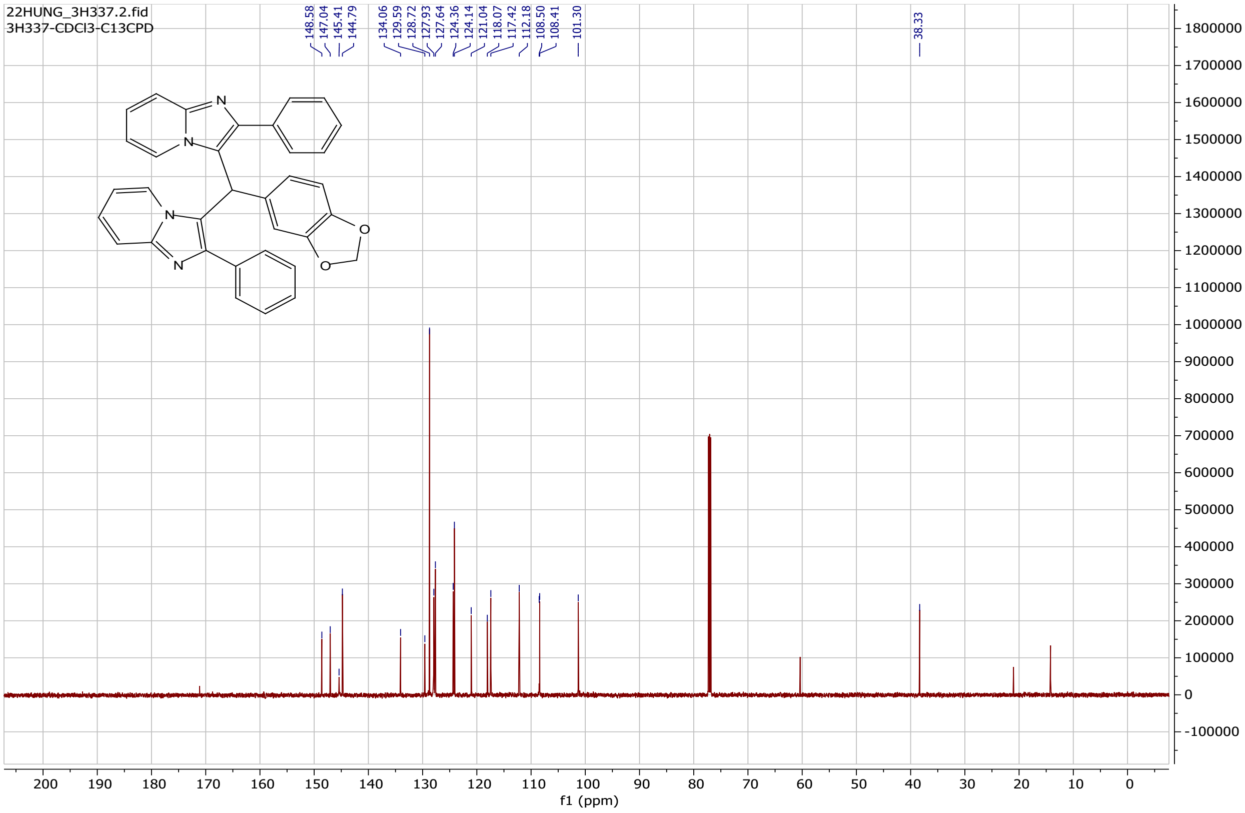

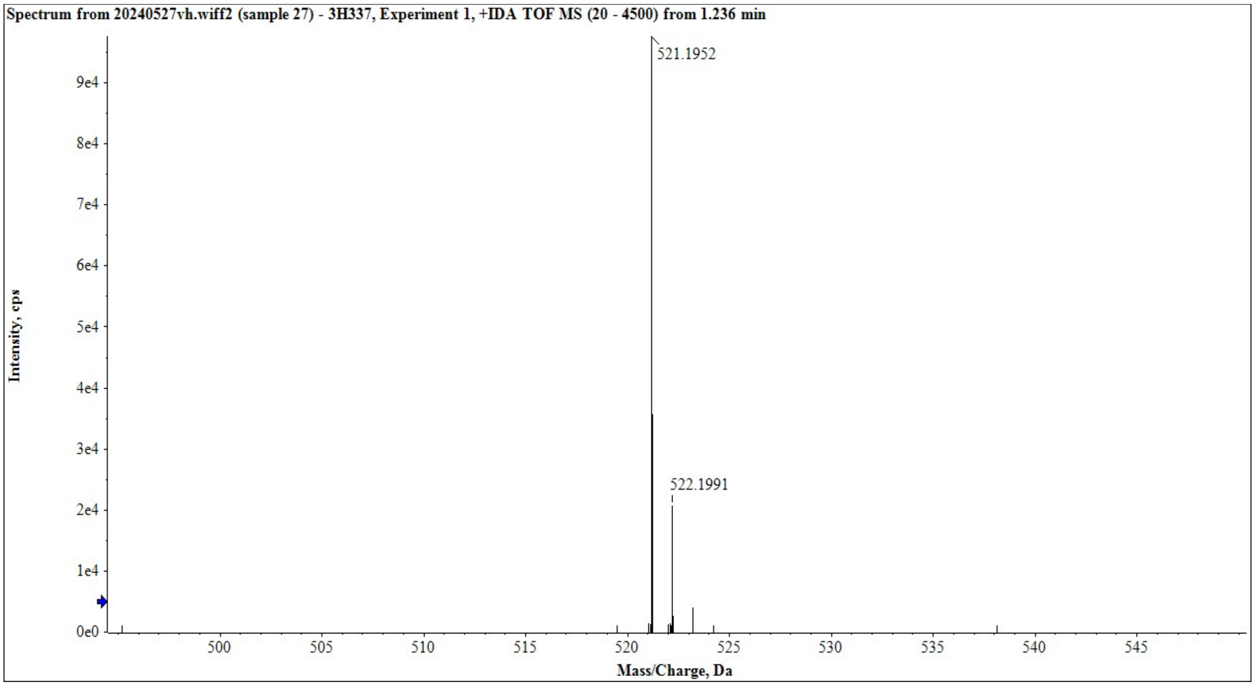

(*E*)3-(hex-1-en-1-yl)-2-phenylimidazo[1,2-*a*]pyridine 5a

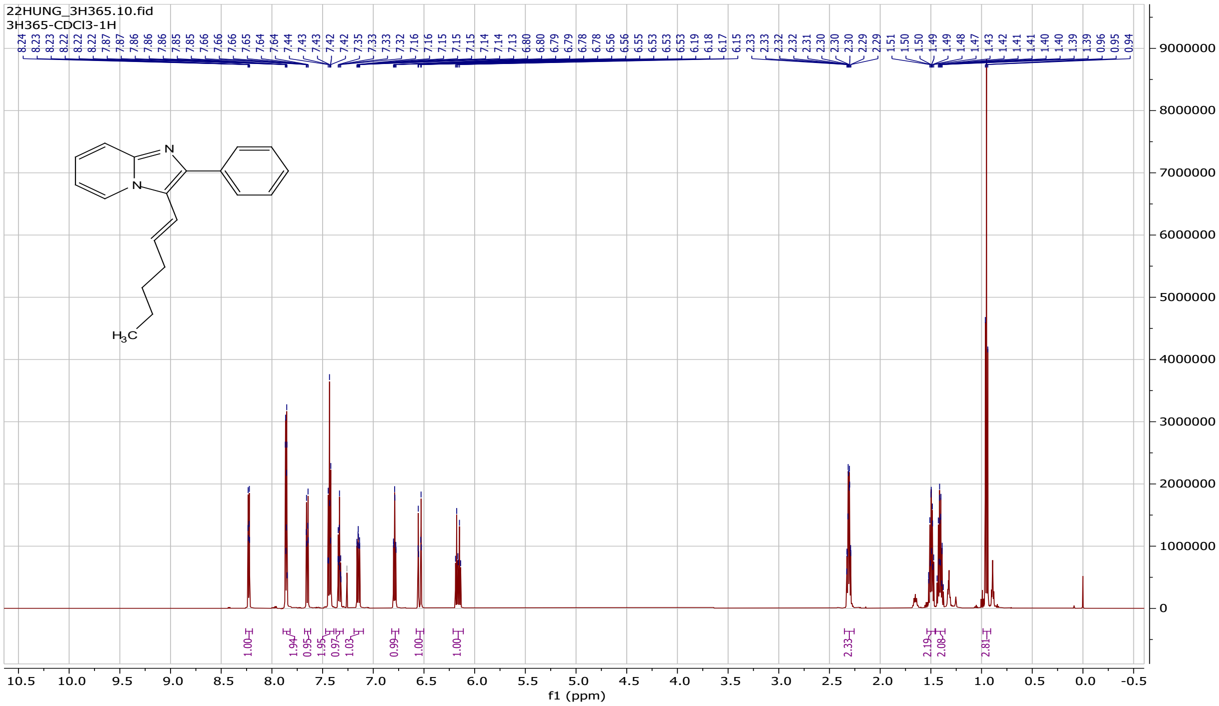

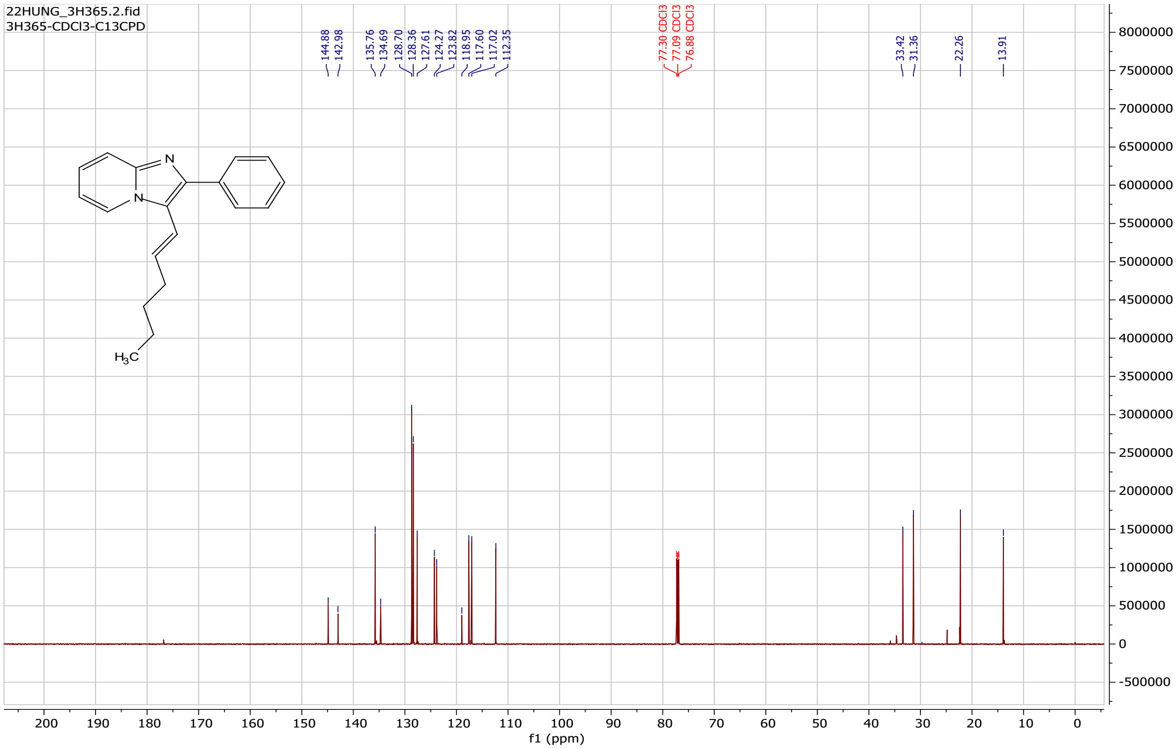
22HUNG_3H365.2.fid
3H365-CDCl3-C13CPD
144.88
142.98
135.76
134.69
128.70
128.36
127.61
124.27
123.82
118.95
117.60
117.02
112.35
77.30 CDCl3
77.09 CDCl3
76.88 CDCl3
33.42
31.36
22.26
13.91
f1 (ppm)

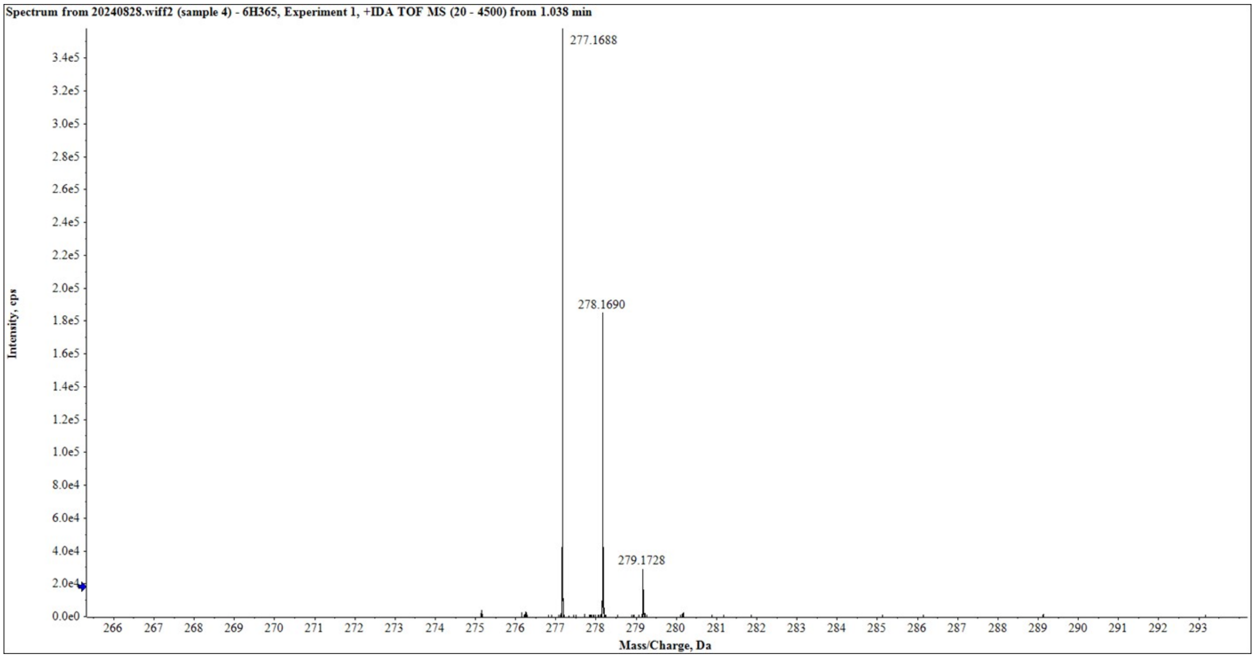
Spectrum from 20240828.wiff2 (sample 4) - 6H365, Experiment 1, +IDA TOF MS (20 - 4500) from 1.038 min
277.1688
278.1690
279.1728
Intensity, cps
Mass/Charge, Da

REFERENCES

1. Lee, J.C., Y.H. Bae, and S.-K. Chang, Efficient alpha-halogenation of carbonyl compounds by N-bromosuccinimide and N-chlorosuccinimde, *Bulletin of the Korean Chemical Society*, 2003, **24**(4), 407.
2. Adhikari, M.V. and S.D. Samant, Sonochemical bromination of acetophenones using p-toluenesulfonic acid–N-bromosuccinimide, *Ultrasonics Sonochemistry*, 2002, **9**(2), 107.
3. Tanis, S.P., et al., PPARγ-sparing thiazolidinediones as insulin sensitizers. Design, synthesis and selection of compounds for clinical development, *Bioorganic & Medicinal Chemistry*, 2018, **26**(22), 5870.
4. Zhao, R., et al., One-Pot Construction of Fluorinated Chiral Alcohols via Sequential Substitution/Asymmetric Transfer Hydrogenation of α-Bromoarylketones and Fluoroalcohols, *Advanced Synthesis & Catalysis*, 2023, **365**(14), 2410.
5. Takizawa, S.-y., et al., Synthesis and Characterization of Novel Iridium Complexes with Ligands of 2-Phenylimidazo[1,2-a]pyridine Derivatives and Application to Organic Light-emitting Diode, *Chemistry Letters*, 2005, **34**(9), 1222.
6. Das, D., et al., *A simple and efficient route to 2-arylimidazo[1,2-a]pyridines and zolimidine using automated grindstone chemistry.* Journal of Heterocyclic Chemistry, 2020. **57**(11): p. 4099-4107.
7. Kalari, S., A.U. Shinde, and H.B. Rode, Methylene-Tethered Arylsulfonation and Benzotriazolation of Aryl/Heteroaryl C–H Bonds with DMSO as a One-Carbon Surrogate, *The Journal of Organic Chemistry*, 2021, **86**(24), 17684.
8. Yang, J., et al., Continuous-Flow Photocatalysis for the Direct C-H Trifluoromethylation of Heterocycles with an Organic Photoredox Catalyst, *European Journal of Organic Chemistry,* 2023, **26**(2), e202201287.
9. Gao, J., et al., 1,1,1,3,3,3-Hexafluoro-2-Propanol-Promoted Friedel–Crafts Reaction: Metal-Free Synthesis of C3-Difluoromethyl Carbinol-Containing Imidazo[1,2-a]pyridines at Room Temperature, *Molecules*, 2023, **28**(22), 7522.
10. Sultana, F., et al., Transition-Metal-Free Oxidative Cross-Coupling of Methylhetarenes with Imidazoheterocycles towards Efficient C(sp2)−H Carbonylation, *Asian Journal of Organic Chemistry*, 2017, **6**(7), 890.
11. Kaswan, P., et al., Synthesis of 3-aroylimidazo[1,2-a]pyridines via CuCl2 catalyzed tandem dual carbon–nitrogen bonding, *Tetrahedron*, 2014, **70**(45), 8539.
12. Meng, X., et al., Copper supported on H+-modified manganese oxide octahedral molecular sieves (Cu/H-OMS-2) as a heterogeneous biomimetic catalyst for the synthesis of imidazo[1,2-a]-N-heterocycles, *Catalysis Science & Technology*, 2016, **6**(3), 890.
13. Jadhav, N.H., et al., A transition metal-free cascade reaction using heterogeneous tin(IV)oxide catalyzed and iodine promoted synthesis of 3-aroylimidazo[1,2-a]pyridines, *Tetrahedron Letters*, 2020, **61**(34), 152250.
14. Liu, P., et al., Synthesis of symmetrical methylene-bridged imidazoheterocycles using DMSO as methylene source under metal-free conditions, *Organic & Biomolecular Chemistry*, 2016 **14**(27), 6523.
